# Work‐hardening Photopolymer from Renewable Photoactive 3,3’‐(2,5‐Furandiyl)bisacrylic Acid

**DOI:** 10.1002/cssc.202000842

**Published:** 2020-07-29

**Authors:** Yann Lie, Alessandro Pellis, Ignacio Funes‐Ardoiz, Diego Sampedro, Duncan J. Macquarrie, Thomas J. Farmer

**Affiliations:** ^1^ The University of York Department of Chemistry Green Chemistry Centre of Excellence YO10 5DD Heslington York UK; ^2^ University of Natural Resources and Life Sciences Vienna Department of Agrobiotechnology Institute of Environmental Biotechnology Konrad Lorenz Strasse 20 3430 Tulln an der Donau Austria; ^3^ RWTH Aachen Institute of Organic Chemistry Landoltweg 1 52074 Aachen Germany; ^4^ Department of Chemistry Centro de Investigación en Síntesis Química (CISQ) Universidad de La Rioja Madre de Dios 53 E-26006 Logroño La Rioja Spain

**Keywords:** enzyme catalysis, furandiyl diacrylic esters, photochemistry, renewable polyester, density functional theory

## Abstract

The design of a photopolymer around a renewable furan‐derived chromophore is presented herein. An optimised semi‐continuous oxidation method using MnO_2_ affords 2,5‐diformylfuran from 5‐(hydroxymethyl)furfural in gram quantities, allowing the subsequent synthesis of 3,3’‐(2,5‐furandiyl)bisacrylic acid in good yield and excellent stereoselectivity. The photoactivity of the diester of this monomer is confirmed by reaction under UV irradiation, and the proposed [2+2] cycloaddition mechanism supported further by TD‐DFT calculations. Oligoesters of the photoreactive furan diacid with various aliphatic diols are prepared *via* chemo‐ and enzyme‐catalysed polycondensation. The latter enzyme‐catalysed (*Candida antarctica* lipase B) method results in the highest *M*_n_ (3.6 kDa), suggesting milder conditions employed with this protocol minimised unwanted side reactions, including untimely [2+2] cycloadditions, whilst preserving the monomer's photoactivity and stereoisomerism. The photoreactive polyester is solvent cast into a film where subsequent initiator‐free UV curing leads to an impressive increase in the material stiffness, with work‐hardening characteristics observed during tensile strength testing.

## Graphical abstract

**Light gives strength**: Synthesis of 3,3’‐(2,5‐furandiyl)bisacrylic acid from renewable 5‐(hydroxymethyl)furfural allows access to a family of new photopolymers. Enzymatic catalysis avoids undesired side reactions, whereas DFT calculations support the proposed [2+2] cycloaddition. Solvent‐cast films are mechanically tested before and after UV‐curing, showing a drastic increase in strength upon cure with work‐hardening behaviour.

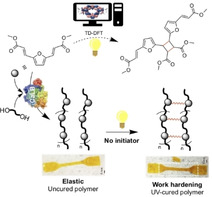

## Introduction

Manmade polymers such as poly(ethylene terephthalate) (PET), high‐density poly(ethylene) (HDPE) and poly(vinyl chloride) (PVC) surround our urban environment and are produced in multi‐ton quantities. Indeed, synthetic thermoplastic production volume is projected to grow from 350 Mt in 2017 to 590 Mt in 2050.^1^ These plastics are used for a wide array of applications ranging from packaging (36 %) and construction (16 %) to textiles (15 %) and others (33 %).^1^ Unfortunately, most plastics currently produced are derived from non‐renewable fossil‐based chemicals such as ethylene, propylene or benzene/toluene/xylenes (BTX). The depletion of crude oil and natural gas has pushed industries to find polymers derived from renewable resources with similar or better properties successfully exemplified by the development of poly(ethylene furanoate) (PEF).^2^ However, polymers are widely utilised in other applications than simple plastics, often incorporating reactive functional groups within the polymer backbone itself but are ultimately still derived from crude oil.

**Scheme 1:**
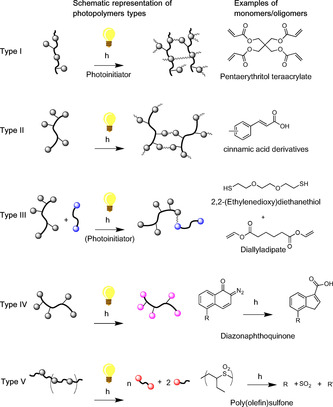
Different types of photopolymers as defined by Crivello and Reichmanis adapted from Crivello and Reichmanis.^5^ Type I: polymerisation/cross‐linking via multiple functional groups reacting but requiring the use of a separate initiator; Type II: intrinsically photoactive groups in monomer/polymer undergo polymerisation without the need for a separate initiator; Type III, two complementary moieties react following photoactivation of one of the moieties, possibly requiring an initiator; Type IV: Molecular structure of reactive site changes upon irradiation but polymer chain does not extend or break; Type V: photocleavage of UV‐active groups in polymer chain/network.

Photopolymers are a class of polymers that are particularly useful in specialised applications such as dentistry, microelectronics or 3D printing.^3^ Under direct or indirect interaction with light (usually in the UV range), the thermo‐physical properties of these polymers changes. Typically, photopolymers are distinguished according to their reactivity to light (Scheme 1). These reactions usually occur in the presence of a photoinitiator such as ketones (benzil or benzophenone, radical initiator), onium salts (iodonium, ammonium salt, cationic initiator) or nitriles (crystal violet leuconitrile, anionic initiator).^4^

In a 2014 review of the area, Crivello and Reichmanis proposed to define five types of photopolymers (Scheme 1).^5^ **Type I** are monomers or short oligomers bearing two or more functional groups that can undergo chain‐growth polymerisation after photoinitiation, leading to a potentially cross‐linked network between the polymer chains. These types of photopolymers, such as those based on pentaerythritol tetraacrylate (Scheme 1), are particularly useful in 3D printing.^4^, ^6^ Upon irradiation, polymerisation and/or cross‐linking occurs, increasing the rigidity of the polymer, which then forms the (stiff) base for another layer of polymer to be applied, making possible the printing of solid 3D objects. **Type II** photopolymers possess intrinsically photoactive moieties which, unlike Type Is, can undergo reactions without the need for a separate initiator. For instance, photopolymers with a constituting unit based on cinnamic acid derivatives (*e. g*. 2,3‐diacetoxycinnamic acid) may form a crosslinked network after irradiation without the need for a photoinitiator.^7^, ^8^ **Type III** photopolymers possess two complementary moieties susceptible to reacting with each other ‐ one photoactive and another not. After irradiation of the photo‐active group (in the eventual presence of a photoinitiator), bond formation occurs between the thus excited moiety and the other. For example, the photoinduced thiol‐ene chemistry, where a thiol group and an alkene undergo a coupling reaction after exposure to light in the presence of a suitable radical initiator can be used to produce Type III photopolymers.^9^ **Type IV** includes polymers containing photoreactive moieties which, under irradiation, convert to another structurally different species but that does not result in chain growth or crosslinking. This is useful in lithography where the solubility of a photoresist is altered upon irradiation allowing removal (positive photoresist) or retention (negative photoresist) of a specific layer of photopolymer after development with an appropriate solvent. A famous example of Type IV is the diazonaphthoquinone – Novolac resin combination (Scheme 1). Finally, photolysis occurs in the **Type V** photopolymers where the UV‐active sites in the polymer chain are cleaved after exposure to light. For example, the [2+2] cycloaddition between cinnamic acid derivatives may be reversed after irradiation at λ <260 nm making such photopolymers both type II and V. Polymers such as poly(olefin)sulfone are also examples of type V photopolymers and considered as potential candidates for extreme UV photolithography applications.^10^

Most monomers currently employed to form photopolymers are commonly derived from fossil resources. For example, acrylate and methacrylate derivatives (Scheme 1, pentaerythritol, diallyladipate) are formed from ethylene, a base chemical issued from cracking of naphtha or crude oil. Cinnamic acid and its derivatives, such as *para‐*hydroxycinnamic acid (*p*‐coumaric acid), are interesting monomers bearing a photoactive group (acrylic acid) capable of forming [2+2] cycloadducts.^11^ Although currently produced industrially from benzaldehyde *via* the Perkin reaction, they are also potentially obtained from lignin decomposition products or extracted from cinnamon, both renewable resources.^11^, ^12^ In 2015, Nguyen et al. developed a Sb_2_O_3_ route towards the use of *p‐*hydroxycinnamic acid derivatives (ferulic and coumaric acids) for the production of poly(ethylene ferulate) poly(ethylene coumarate).^13^ However, the photochemical properties of these were not investigated.

On the other hand, thermally induced cross‐linking of bio‐based compounds has been investigated in greater depth. Notably, a recent different approach by Sousa et al. consisted of the copolymerisation of 2,5‐furandicarboxylic acid with fumaric, succinic acid and 1,3‐propanediol, making use of the fumaric acid unsaturation for thermally induced crosslinking.^14^, ^15^ Unfortunately, only low molecular weight polymers could be obtained (*M*_n_ 1.6‐3.2 kDa) and this required the presence of a thermal initiator (2‐hydroxymethylmethacrylate). The photoactivity of these polymers was not studied.

**Scheme 2:**
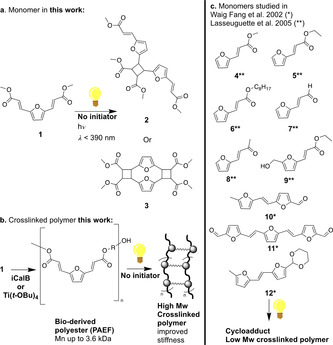
a) Monomer used for the enzymatically catalysed transesterification, **1**, and possible cycloadduct product. b) General polycondensation and crosslinking scheme. c) Previously reported UV‐active furan‐derived monomers from Lasseuguette et al. and Waig Fang et al.^16^, ^18^

Only a few examples in the literature make use of photo‐active bio‐derived molecules, such as furan derivatives, for the synthesis of photopolymers. In the early 2000s, Gandini's group synthesised a series of UV‐active furan derivatives aiming at using them as chromophores in photopolymers.^16^, ^17^, ^18^ Primarily, Waig Fang et al.^16^ realised the synthesis of dimers of furfural and 5‐methyl furfural (Scheme 2c, **10**–**12**). These compounds already displayed promising UV‐active properties as their [2+2] cycloadduct could be obtained after irradiation (1–25 h, ∼385 nm). The further grafting of these compounds on polyvinyl alcohol polymers by acetalization retained the UV‐active properties of the chromophores. The use of this photopolymer for lithographic applications was patented in 2002.^19^ Lasseuguette et al. modified furfural by Knoevenagel‐Doebner condensation (Scheme 2c, **4**–**6**) or aldol condensation with acetaldehyde or acetone (Scheme 2c, **7** and **8**) and 5‐(hydroxymethyl)furfural (HMF) *via* a Wittig reaction (HMFAE, Scheme 2c, **9**).^17^ The later was copolymerised with ethyl‐6‐hydroxy hexanoate and hydroxy ethyl esters forming a photopolymer containing up to 10 % **9**.^18^

Acceptable molecular weights between 3.8‐6.8 kDa and *M*_n_ between 1.6‐2.9 kDa were obtained using K_2_CO_3_, a mild catalyst employed to preserve the reactive unsaturated furan group.^18^ However, the use of co‐monomers resulted in low levels of cross‐linking during the cure. This is owing to the low chromophore content and excessive thickness of the film. Other attempts to use the UV‐active properties of unsaturated furan derivatives were described for the modification of cellulose for biocompatible polymer synthesis or kinetics study.^20^, ^21^ However, in the first case, the unsaturated furans moieties were only substituting pendants of the cellulosic polymer, and not a constitutive unit thereof.^20^ Additionally, the in‐depth kinetic report employed a methacrylate substituted furan for their study which deviates away from the potentially sustainable character of the monomer.^21^

We report the development of an alternative photopolymer system based around a renewable furan scaffold, (polyesters from furandiyl bisacrylic esters, PFAE). To increase the extent of cross‐linking between polymer chains we proposed to synthesise a highly UV‐active monomer, compound **1** (Scheme 2a, **1**), bearing two photo‐active sites (two exo‐furan C=C moieties). We also sought to incorporate a greater proportion of this monomer into the oligoesters by avoiding the use of photo‐inert co‐monomers. The known sensitivity of photoreactive monomers, such as **1**, led us to study milder polycondensation conditions, including the use of immobilised enzyme catalysts that effectively preserved the UV‐absorbing unit. In this way, we prepare an innovative Type II renewable photoactive polymer, where the use of an initiator could also be avoided. We also disclose mechanistic insights of the curing reaction pathway by studying the formation of a [2+2] cycloadduct direct from monomer **1**, and further support the [2+2] cycloaddition mechanism through TD‐DFT calculations.

## Results and Discussion

**Figure 1:**
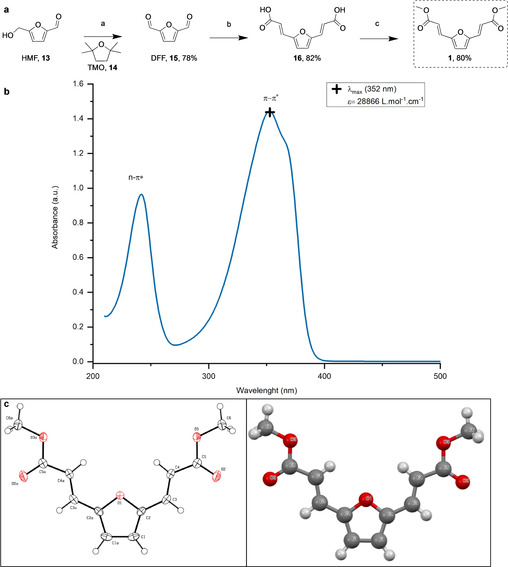
a) Synthetic route to unsaturated monomer **1** a. MnO2 (excess; e. g., 7.73 g), 1 % HMF solution in TMO (e. g., 1 g in 100 mL), 75 °C semi‐continuous 1 mL/min, residence time ∼3 min, total reaction time 100 min. b. malonic acid (4 equiv.), piperidine (0.2 equiv.), pyridine, 80 °C overnight then reflux 2 h. c: MeOH, H_2_SO_4_ (0.4 equiv.). b) UV/Vis spectrum recorded in EtOH at 50 *μ*M in 1 cm quartz cuvettes. c) single‐crystal XRD structure with 50 % thermal ellipsoid (left) and balls and stick representation (right)

**Monomer synthesis**: Monomer **1** was described as early as 1914 and was recently used in reactions with benzyne for the production of potential terephthalate replacement.^22^, ^23^ However, to the best of our knowledge, no details on **1**’s photoactive properties have thus far been presented. Synthesis of **1** begins with the oxidation of HMF to 2,5‐diformyfuran (DFF, Figure 1, **13** and **15** respectively). In its own right, DFF can be considered a renewable chemical with high potential for synthetic applications. The presence of two aldehydes groups offers many possibilities for further modification via Wittig reaction, Schiff bases formation, aldol condensation, etc.^24^, ^25^ Despite this promise, few reports make use of this compound partly owing to its current high retail price (123 £.g^−1^ Sigma Merck *accessed 10/3/20*) and instability upon prolonged storage. Additionally, the synthesis of DFF from HMF commonly requires molecular oxygen or expensive transition metal (co)‐catalyst (*e. g*. Ru) which further reduces the applicability of DFF‐derived reaction at a higher scale.^26^, ^27^, ^28^, ^29^

We conducted the oxidation of HMF in a semi‐continuous manner using a column packed with commercial electrolytically precipitated 88 % MnO_2_ (Supporting Information, Figure S1) and the toluene alternative 2,2,5,5‐tetramethyloxolane (TMO, Figure 1, **14**) as the solvent.^30^ The non‐peroxide forming TMO used in this reaction was recovered and reused after distillation. In addition, this system did not require the use of inert atmosphere, molecular oxygen or co‐catalyst as previously reported.^26^, ^31^, ^32^

The solution collected after one passage through the column was analysed by GC‐FID to assess the conversion of HMF. If the conversion was not complete, the solution was recirculated through the column. When no more HMF conversion was observed, the solution was evaporated under reduced pressure. In this manner, DFF could be obtained with very high selectivity and purity after the first passage through the column and was used in the next step without further purification. The first passage through the system yielded up to 78 % of pure DFF isolated as a white solid.

The type of commercial manganese dioxide used for the reaction was found to be crucial. Indeed, when using a 99 % pure MnO_2_ almost no conversion could be observed. Porosimetry analysis showed that the BET surface area of the 88 % pure MnO_2_ was 6 times higher than the 99 % pure MnO_2_ (Supporting Information, Table S1). Furthermore, as demonstrated by the DFT analysis conducted by Hayashi et al., the crystal structures of MnO_2_ catalysts are determinant for their activity owing to the difference in the vacancy formation energy (VFE) of the oxygen planar sites.^32^ Thus, the powder XRD analysis (Supporting Information, Figure S4) of both catalysts used revealed that the 99 % MnO_2_ had a crystalline structure similar to the *β*‐MnO_2_ synthetized by Hayashi et al. for which they computed a VFE of 3.25 eV. The domain frame (crystallite size) was calculated using the Scherrer equation which gave an average value of 28.8 nm. Contrarily, the 88 % MnO_2_ had a more amorphous structure, with a domain frame of 5.9 nm, explaining the higher surface area observed by porosimetry, and a crystal structure close to the ϵ ‐MnO_2_ described before. The smaller domain frame between the two types of MnO_2_ may also suggest that more active sites are available in the 88 % MnO_2_. No oxygen VFE was computed by Hayashi et al. for the ϵ ‐MnO_2_ owing to its less defined crystalline system, however, the related γ‐MnO_2_ had a VFE of 3.84 eV for type *α* oxygens and of 3.15 eV for type *β* oxygens. The presence of more *β* oxygens in the 88 % MnO_2_ may explain its higher oxidative power. Finally, EDX‐SEM analysis (Supporting Information Figures S5‐S8) of the catalysts showed a higher Mn/O ratio in the 99 % MnO_2_ (84.5 : 14.6 by weight) corresponding to an empirical formula of MnO_1.64_ compared to the 88 % (74.6 : 23.7 by weight) corresponding to a MnO_1.1_ at the surface. Traces of precursors KMnO_4_ may explain both the higher oxidative properties of the 88 % MnO_2_ and the Mn/O ratio (together with the presence of MnOOH or MnO species) observed in the samples. Finally, SEM images did confirm the more amorphous structure as suggested by powder XRD. Overall, these observations may explain the much higher oxidative property of 88 % MnO_2_ compared to the 99 % MnO_2_.

Our system for the synthesis of DFF allows facile multiple reuses of the MnO_2_‐packed column. Indeed, after the first passage of a first 1 %wt HMF solution, the column was flushed with TMO, and another fresh HMF solution passed through the system. For this second solution, the first passage through the column allowed for a conversion of 53 % of HMF to DFF which reached 87 % after 7 passages (measured by GC‐FID, Supporting Information Table S2). This loss of activity was initially thought to be owing to the formation of the inactive species MnOOH as suggested previously.^26^, ^31^ Hence, another system was designed where pure O_2_ was bubbled continuously in the HMF solution before the column to favor the re‐oxidation of the partially reduced MnO_2–d_ to MnO_2_ and avoid the formation of the red‐ox inactive species. Unfortunately, no change in the recyclability of the catalyst was observed, possibly owing to the low solubility of O_2_ in TMO.

However, calcination under static air at 300 °C for 1 h allowed for some recovery in activity and a one‐pass conversion of 30 % of HMF to DFF could be reached (Supporting Information Table S3). The EDX‐SEM showed a slight reduction in the %O content (Mn/O post calcination, post‐reaction 53.8 : 44.1 by weight) as well as more crystalline structures after calcination than before (Mn/O pre calcination, post‐reaction 48.1 : 50.9 by weight). Additionally, porosimetry analysis showed an increase in the average pore width of the mesopores, from 8.8 nm to 11.6 nm after calcination (Supporting Information, Table S1) which together with the EDX‐SEM analysis may account for the regained activity of the 88 % MnO_2_ catalyst.

Previous reports made use of the aldehyde manifold present in furfural or HMF for extending the conjugation of these compounds by incorporating an unsaturated moiety via Knoevenagel condensation.^17^, ^23^ DFF was thus considered to be an excellent candidate for the double Knoevenagel‐Doebner condensation reaction and afforded the *E‐E* intermediate **16** (Figure 1, **16**) in 82 % isolated yield with 100 % stereoselectivity. Although pyridine was used as solvent in this Knoevenagel condensation, encouraging results have been reported using ionic liquids (up to 62 % yield) or solvent‐free systems (99 % using malononitrile).^33^, ^34^, ^35^, ^36^ Microwave or ball‐milling have also been used with success for the Knoevenagel reaction, thus offering scope for further improvements to the protocol.^33^, ^37^ Subsequent esterification of **16** afforded the monomer **1** (Figure 1) in 80 % yield, giving an overall yield from HMF of 51 %. Interestingly, only the *E‐E* isomer of **16** was obtained as confirmed by the high J coupling constant of the alkene protons (δ=7.34 & 6.34 ppm, J_c=c_= 15.8 Hz, Supporting Information Figure S11). The higher stability of the *E‐E* isomer, owing to reduced electronic interaction between the two carboxylic acids, and to a lesser extent a reduced steric effect may explain the high stereoselectivity of the Knoevenagel‐Doebner reaction. The obtained single‐crystal XRD structure (Figure 1c) of the esterified product **1** further confirmed the *E‐E* configuration, while the ease of crystallisation of **1** as shiny pale brown flakes likely indicated the rigid planar structure this compound sits in.

Finally, the strong UV absorbance (Figure 1b, ϵ =28866 L.mol^−1^.cm^−1^) of **1**, was attributed to the π‐π* transition of this compound allowed by symmetry as later confirmed by TD‐DFT calculation (see below). Absorption in the UV−A region, likely results from a reduction in the HOMO‐LUMO orbitals energy gap owing to the extended conjugation of **1**. This confirmed the possibility to use this monomer as the UV‐active section of a polyester for further UV‐curing, assuming the extended conjugation could be preserved from the prior polycondensation reaction.

***Synthesis of polyesters of furandiyl diacrylic esters (PFAE) from 1***: First, **1** was combined with the aliphatic diol 1,8‐octanediol (ODO) and the chemo‐catalysed (K_2_CO_3_, Zn^II^ acetate, Zr^IV^ *iso*‐propoxide or Ti^IV^ *tert*‐butoxide) synthesis of aromatic‐aliphatic polyesters was carried out under mild two‐stage polycondensation conditions (95 °C under inert atmosphere Ar or N_2_ for 24 h with excess (2 equiv.) of ODO, 5 mol% catalyst, then 4 h under high vacuum, <1 mbar). Lower temperatures were employed owing to the potentially sensitive nature of the acrylic ester units. Unfortunately, with most catalysts no polymeric product was isolated, only fractions of short oligomers (for the THF‐CHCl_3_ soluble fraction see Supporting Information, Figure S17), residual monomer **1** (specifically Ti^IV^, Zr^IV^ and Zn^II^ Supporting Information Figure S17) and THF‐CHCl_3_ insoluble cross‐linked material. Synthesis carried out using Ti^IV^ *tert*‐butoxide yielded polymers with the highest molecular masses via chemo‐catalysis (*M*_n_ of 1.2 kDa and *M*_w_ of 2.4 kDa) (see Supporting Information Table S4 for additional data on the chemo‐catalytic polymerizations). In 2005 Lasseuguette et al. reported similar issues while attempting to polymerize 2‐hydroxymethyl‐5‐furanacrylic acid ethyl ester (HMFAE, Scheme 2, **9**), noting that the Ti(*t*BuO)_4_ catalyst tended to complex with furan rings reducing its catalytic activity.^18^ This could explain the low molecular weights obtained in our chemo‐catalysed polycondensation. The presence of the two *exo*‐furan C=C groups may also increase the ability of **1** to complex with certain metal salts explaining the low catalytic activity of usually very active salts such as Zn(OAc)_2_ or Zr isopropoxide. In their work, Gandini's group elegantly improved the polycondensation reaction by co‐polymerizing HMFAE with ethyl‐6‐hydroxyhexanoate using milder K_2_CO_3_ as the catalyst and temperatures ranging between 95 and 120 °C obtaining polymers of *M*_n_ 1.6–2.9 kDa and *M*_w_ 3.8–6.8 kDa and yields of 60–85 %.^18^ In our hands, this catalyst only led to short oligomers (Supporting Information, Table S4) despite the good conversion observed by ^1^H‐NMR (Supporting Information, Figure S17). Furthermore, a previous study by Krhouf et al. on bifuranic polyesters suggested that the presence of labile hydrogens α to the two furan rings lead to radical side reactions, branching and cross‐linking.^38^ The unsaturation (C=C) on both sides of monomer **1** likely reacted in a similar, radical fashion, leading to the insoluble material described above. The higher molecular weight obtained with Ti(IV) might result from its ability to act as a free radical initiator, thus favouring branching and increasing chain length but eventually leading to gelation.^39^, ^40^

**Figure 2:**
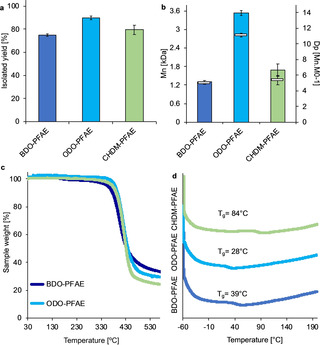
iCaLB‐catalysed synthesis of PFAE in DPE as the organic medium at 85 °C and 20 mbar. a) isolated yield after MeOH precipitation and three washing steps, b) number average molecular weight (*M*_n_) calculated via CHCl_3_ GPC and degree of polymerization (DP), c) thermogravimetric analysis (TGA) and d) differential scanning calorimetry analysis (DSC). Colours legend: blue=polymers containing 1,4‐butanediol (BDO, C4) as the aliphatic, linear diol, red=polymers containing 1,8‐octanediol (ODO, C8) as the aliphatic, linear diol and green=polymers containing 1,4‐cyclohexanedimethanol (CHDM, Cc8) as the cyclic, rigid diol.

Polycondensation reactions using an enzymatically‐catalysed transesterification reaction in organic media can be performed at low temperature and avoid metal salts catalysts. This could prevent previously unwanted side reactions to occur.^41^ Based on a recently developed protocol, we selected an immobilized preparation of *Candida antarctica* lipase B (iCaLB) as catalyst and diphenyl ether (DPE) as the solvent.^42^, ^43^, ^44^ Results from the enzyme‐catalysed synthesis were encouraging, with molecular masses ranging from 1.3–3.6 kDa for *M*_n_ and 3.3–26 kDa for *M*_w_ depending on the used diol. Part of the very high *M*_w_ may be attributed to branching that may have started to form before the GPC analysis. Nevertheless, isolated yields above 74 % were obtained for all synthesised polymers (Figure 2a). As can be observed in Figure 2b, the polyesters prepared from ODO as the aliphatic diol were optimum both in terms of molecular masses and isolated yields (see Supporting Information Table S7 for additional data on the enzymatic synthesis in DPE). The temporary disruption of **1**’s extended conjugation (Supporting Information, Figure S36) owing to serine hydrolase's mechanism may explain the low *M*_n_. The continuous removal of volatile by‐products (methanol) and the long reaction time (4 days) may explain the unusually good yields obtained for this unsaturated aromatic monomer. Moreover, under other carefully optimised conditions (*e. g*. diol choice, solvent‐free, vacuum), CaLB was shown to perform well for the (trans)esterification of other unsaturated aromatics such as ferulic acid.^45^

From thermal analysis (Figure 2c) all synthesized polymers possessed a similar T_d50_ between 430 and 438 °C (see Supporting Information Table S5 and Figures S23–25 for additional TGA data). The T_g_ from the 2^nd^ heating decreases from 39 °C to 28 °C when increasing the aliphatic diol chain length from C4 (1,4‐butanediol, BDO, Figure 2d, blue line) to C_8_ (ODO, Figure 2d, red line). The polymers containing the cyclic diol cyclohexanedimethanol (CHDM) showed a higher T_g_ of 84 °C (Figure 2d, green line, see Supporting Information Table.S6 and Figures S26, S27 for additional DSC data). The structures of the polyesters, fully preserving the furan ring, C=C and *E‐E* stereochemistry, were confirmed via ^1^H and ^13^C NMR spectroscopy (Supporting Information, Figures S15, S16). MALDI‐TOF analysis was performed on all samples, agreeing with the molecular weight distribution observed by GPC analysis. End group analysis shows the following: major being ester‐diol, minor being ester‐ester, diol‐diol and some traces of cyclic polyesters (Supporting Information, Figures S28–32).^42^

In order to investigate the cyclic thermal behaviour of this novel class of polyesters, DSC analysis on the ODO‐PFAE polymer was repeated for 6 cycles (temperature range −60–200 °C, 5 °C/min). Interestingly, a slight increase of the T_g_ (from 30 °C to 37 °C) and a decrease of the ΔCp (from 0.19 J/(g °C)^2^ to 0.13 J/(g °C)^2^) was observed (see Supporting Information, Table S9, for additional DSC data). The observed variation can be owing to the shift of the polymer's crystalline/amorphous balance towards a slightly higher crystallinity, highlighting some variance in thermal behaviour upon multiple heat cycles.

***UV***‐***induced photoreaction of 1 and mechanistic insight via DFT computations***: Photoactive properties of monomer **1** was studied before polycondensation to ascertain this compound does undergo [2+2] desired photo‐induced cycloaddition and thus is suitable for preparation of Type II photomonomers. Purified compound **1** was subjected to UV‐irradiation using a custom‐made LED torch (365 nm, 1250 mW flux output, see Supporting Information, Figure S2) following the procedure described in the experimental section.

**Figure 3:**
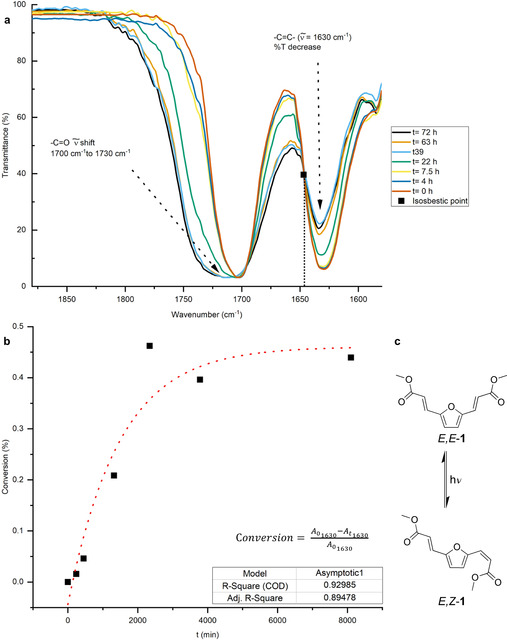
a) FT‐IR spectroscopy of UV‐ monomer after different irradiation time between 1580 and 1880 cm^−1^. Isosbestic point is observable at 1640 cm^−1^; b) Conversion calculated according to reference ^[17]^, at 1630 cm^−1^. Data were fitted with OriginLab (version 2019) using an exponential model (asymptot1); c) Proposed *E‐E* to *E‐Z* conversion of **1** upon irradiation.

Reaction progression was studied by FT‐IR spectroscopy (Figure 3a), showing disappearance of the *exo*‐furan C=C signal at 1630 cm^−1^ and C=O shift from 1700 cm^−1^ to 1715 cm^−1^ indicating loss of carbonyl conjugation. In addition, the presence of an isosbestic point at 1640 cm^−1^ suggests that the photoreactivity of monomer **1** follows a single reaction pathway.^17^, ^46^ Moreover, conversion of **1** to its photoproducts (Figure 3b), seems to follow an exponential trend with a horizontal asymptote at roughly 50 % conversion. Limitations owing to the experimental set up (*e. g*. LED maximum emission far from maximum absorbance, low power, the thickness of monomer layer, etc.) and the nature of the irradiated material (crystalline phase) likely limited the complete conversion of monomer **1** crystals to cycloadducts.

Analysis of the ^1^H NMR spectra of the material obtained after 24 h of UV irradiation, confirmed the presence of monomer **1** as well as its single [2+2] cyclo‐adduct **2** (Supporting Information, Figure S18, S19). However, the presence of the double cycloadduct **3** was not conclusively observed. As the UV reaction was conducted in the solid state, the formation of a single cycloadduct was likely preferred owing to the unsaturated side chains orientation in the crystals. In addition, after very long exposure to light (>72 h), the resulting material was no longer completely soluble in CDCl_3_. The presence of a complicated mixture of oligomers arising from the light‐induced self‐polymerisation of **1** was still observable by ^1^H‐NMR spectroscopy (Supporting Information, Figure S20). This suggests a reaction of **2** with another molecule of **1**, and further growth of oligomers in this manner is more likely.

**Figure 4:**
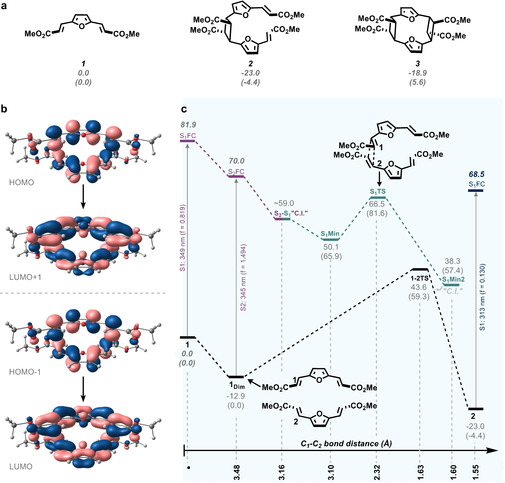
a) Relative stability of compound **1**, single cycloadduct **2** and double cycloadduct **3**. b) Orbital transitions corresponding to Frank‐Condon excitation of **1 dim** at 345 nm (f=1.494). c) Potential energy diagram of cycloadduct formation. Energy values and free energies (in parentheses) are in kcal/mol.

The reverse of the cycloaddition, also known as “crack‐healing” for polymers derived from cinnamic acid, proved unsuccessful in our hands.^47^, ^48^ Attempts to irradiate the cross‐linked polymer of **1** with a custom‐made UV‐lamp (5 W, max emission 253 nm) only led to further cross‐linking (followed by FT‐IR, Supporting Information Figure S35). The excitation of product **2** was explored by TD‐DFT (see below) and the transition is located in the double bonds of **2**. This implies that the cyclobutane moiety is not in the chromophore, which likely explains the difficulty of reversing the [2+2] cycloaddition photolytically (Figure 4c). The potentially reactive bright state reached after absorption at 293 nm (f= 1.41) may result in the formation **3**. Further studies are underway to assess the reversibility of the cycloaddition, with the ambition of also preparing a hybrid **Type II**‐**Type V** photopolymer.

To explain some of the features observed experimentally regarding the cycloadduct formation, TD‐DFT studies were carried out on the monomer **1** and the dimer **1_dim_** (see computational details). First, we explored the relative stability of the initial monomer **1** versus the cycloaddition products (Figure 4a). The single cycloadduct **2** is more stable than the initial monomer by 4.4 kcal/mol. In contrast, the double cycloaddition product **3** is 5.6 kcal/mol less stable than the initial monomer, suggesting that the photoreaction of only one double bond per unit is the most plausible scenario as experimental results indicated (∼50 % conversion by FT‐IR). The high energy of intermediate **3** is associated with high strain owing to the presence of two cyclobutane rings in the same structure, forming a macrocycle that distorts the furan rings owing to spatial proximity.

The formation of dimer adduct in the ground state is isoenergetic with respect to monomer **1**. Both species can absorb light at similar wavelengths (345 and 349 nm, **1** and **1_dim_** respectively), which is associated with the π‐π* excitation. In the case of **1_dim_** (Figure 4b), the bright state corresponds to **S_2_**, which is populated through HOMO‐1 – LUMO and HOMO – LUMO+1 transitions, that are correlated by symmetry and are paired by the orbital phases. Once the dimer reaches S_2_ (**S_2FC_**), the system relaxes in the excited potential energy surface when C_1_<C_2_ bond distance is reduced. During this process, S_2_ and S_1_ states become very close in energy and eventually a crossing between both states should take place. Further relaxation of the system along S_1_ should allow the population of a minimum in the excited state (**S_1_Min**). From here, the formation of the cycloadduct occurs through a transition state (**S_1_TS**) with a free energy barrier of 15.7 kcal/mol. Finally, the system reaches a S_1_‐S_0_ energy degeneracy region, which allows the generation of the cycloadduct **2** in the ground state. The possibility of an adduct formation in the triplet state was also explored (although curing was carried out in the presence of oxygen, which could hamper triplet state processes). Under these circumstances, the cycloaddition reaction is also possible (Supporting Information, Figure S33). The high barrier in the excited state can explain the difficult formation of the cycloadduct in solution owing to the low concentration of **1** and the possibility of alternative deactivation pathways. In contrast, in the solid state, where monomers **1** are much closer and molecular movements are hampered, reaction led to the light‐induced cycloaddition. In solution, isomerisation from *E‐E* to *E‐Z* configuration (Figure 3c) is likely to occur since traces of the *E‐Z* isomer were observed by ^1^H‐NMR spectroscopy after irradiation of the sample (Supporting Information, Figure S34).

***UV***‐***induced curing of ODO***‐***PFAE and impacts on mechanical behaviour***: UV‐induced curing of the previously synthesised polymer (ODO‐PFAE) was conducted in the same fashion as for the monomer **1** (see experimental section). An almost complete reduction of the *exo*‐furan C=C signals (followed by FT‐IR spectroscopy, Supporting Information Figure S21) confirmed the success of the curing procedure, substantiating this as a **Type II** photopolymer. In addition, the thus obtained cured material was visually neither soluble in THF nor CHCl_3_, suggesting that the desired cross‐linking had occurred. An extraction test using either THF‐*d_8_* or CDCl_3_ affirmed minimal solubility, with masses of cured strips showing no (THF) or low (<1 %, CDCl_3_) reduction upon mixing in the solvents for 1 h. Further NMR analysis (Figures S37–S39) of the recovered solvents and use of a standard (dimethyl maleate) suggests extraction of just ∼1.4 % and ∼2.1 % for THF and CDCl_3_ respectively. Although low, this level of extractable content would suggest that further improvements in the extent of cure may still be possible, for example by using a more intense source of UV rather than the LEDs (365 nm, 1250 mW output) used in this preliminary study. To confirm further the success of the procedure ^13^C solid state NMR spectroscopy was conducted (Supporting Information, Figure S22), this previously suggested as the method of choice to follow this type of reaction.^14^, ^49^ Reduction of the alkene signal (∼130 ppm) and appearance of a broad signal corresponding to cyclobutane (40–50 ppm) in ^13^C solid state NMR spectroscopy again supports success of the desired photoreaction.

**Figure 5:**
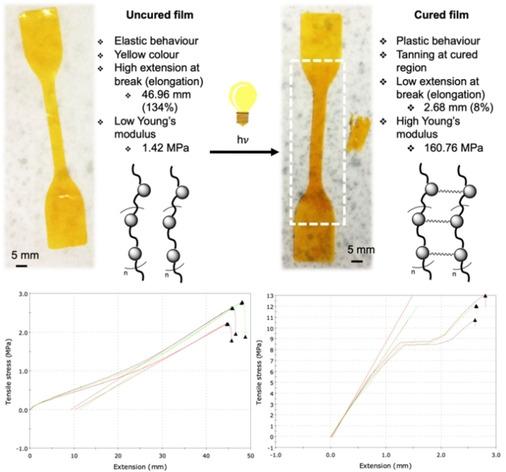
Stress‐strain curve of uncured film (left) and UV‐cured film (right). Dotted square shows the region where tanning of the polymer occurred. Pictures of the film (with enhanced contrast) shaped for analysis are displayed below the stress‐strain curves. Initial separation between clamps of the tensile strength measuring instrument set at 3.5 cm.

In order to test the mechanical properties of the ODO‐PFAE polyester, a film of the un‐cured material was prepared by solvent casting from CHCl_3_, this solvent selected for ease of removal. Prior to curing, the film's stress‐strain curve was measured (Figure 5, left). The un‐cured ODO‐PFAE film displayed a flexible, elastic behaviour, as demonstrated by steady increase in extension and tensile stress resulting in a high extension at break, 46.96 mm±1.59 mm and a small modulus 1.42 MPa±0.11 MPa. Post‐test the non‐cured strip fragment showed elasticity, reforming its original shape.

After UV curing, noticeable “tanning” of the strips was observed (Figure 5, white dotted square) and stiffness of the material greatly increased. The modulus of the cured polymer increased dramatically to 160.76 MPa ±14.98 MPa whereas its extension at break dropped to 2.68 mm±0.1 mm. Comparatively, a furan‐based copolymer obtained with acrylated 7,10‐dihydroxy‐8(E)‐octadecenoic acid (DOD) possessed a tensile strength of up to 19 MPa after UV‐curing.^50^ Post‐test, the herein obtained cured polymer was brittle with no elastic behaviour as observed pre‐cure.

Moreover, the cured polymer displayed a strain‐hardening behaviour as suggested by the plateau observed on the stress‐strain curve (Figure 5 bottom right) around 8.5 MPa followed by a steep increase in tensile stress. This type of strain‐hardening behaviour is commonly observed in plastics such as polypropylene and polyethylene, and usually explained by a reorientation of the polymer fibres in the direction of the stress as well as lamellar crystals.

The UV‐curing thus had a significant impact on the material properties. The higher stiffness and insolubility resulting from cross‐linking between the polymer chains could lead to its potential use as a negative photoresist or in 3D printing technologies. For instance, the stereolithography of this material may be conducted in the melt (80 °C) using its photoreactivity to harden each layer being printing with the help of a laser. The Young's modulus of the cured polymer is relatively low at 160.76 MPa compared to common commercial products used for 3D printing (between 1–10 GPa).^6^, ^51^ However, Sutton et al. reported the use of lignin‐resins blends with properties close to the one reported here (Young's modulus of 370 MPa and 7.6 % elongation) with good 3D‐printing results.^52^ Furthermore, most of today's formulations contain additives which offer the possibility to tune properties of a polymer/oligomer for specific applications (*e. g*. 3D printing or photolithography).^51^ Carbon nanotubes or lignin extract have for example been utilised to significantly stiffen commercial material resulting in an increased Young's modulus.^53^, ^54^ Alternatively, the insoluble character of this polymer following UV‐curing may find use in lithography. Once the surface to be etched is coated with this photopolymer, a UV‐protecting mask may be applied on surfaces to later solubilise. The UV exposed sections are stabilised via crosslinking, and then solvent washing removes un‐crosslinked polymer from the UV‐protected areas. Zhong et al. reported the formation of highly conductive photolithographically patternable ionogels from ionic liquids which displayed comparable Young's modulus (9.57 MPa) and strain (15 %) to our materials.^55^ Microelectronics for instance, depend heavily on this process for the design of transistors. Use of photopolymers is anticipated to diversify further, exploiting this unique controllable switchable behaviour. For instance, a photopolymer has recently been used to lock the alignment of boron nitride nanotubes in porous membranes for the generation of electricity from salinity gradient.^56^ Yet, due the utilisation of non‐renewable resources such as critical elements and fossil‐based (photo)polymers, 3D printing technologies or the microelectronics sector has yet to meet the criteria for a transition to sustainability.

## Conclusion

The advent of photopolymers revolutionised many industrial sectors, in particular (micro)electronics for which photolithography techniques allowed miniaturisation of circuit size. Photopolymers are highly sought after for their ability to crosslink and stiffen in 3D printing applications, while further developments will continue to find new applications their unique controllable properties.

The work herein represents a major advance towards a more sustainable design of photopolymers. Synthesis of monomer **1** from renewable HMF using alternative safer solvents (2,2,5,5‐tetramethyloxolane), non‐precious metal catalyst (MnO_2_) and semi‐continuous methods is an improvement on previously reported synthesis. Furthermore, not only was the polycondensation of this chromophore possible by enzymatic catalysis, but it also showed an improvement in terms of yield, *M*_n_ and chemical inertness compared to conventional metal‐based catalysts. Milder enzymatic catalysis conditions advantageously preserved the photoactive nature of the monomer for which TD‐DFT calculation unveiled the mechanistic pathway of its light‐driven [2+2] cycloaddition. This pericyclic reaction was controllably utilised for the UV‐curing of photopolymer filmstrips that dramatically changed their physio‐chemical properties, becoming stiffer and insoluble in commonly employed solvents. The observation of work‐hardening for the cured polyester further diversifies potential applications where strengthening under induced strain in desirable.

The physical properties of such photopolymer are likely to be tuneable by a judicious choice of diols or copolymers, showing the versatility of the approach used here. This new family of photoreactive furan polyesters will be of significant interest for the development of renewable photoinduced lithographic, 2/3D printing, coating and adhesive technologies.

## Experimental Section

All data used in the preparation of this manuscript is contained within this document, the Supporting Information, or available on request from DOI: 10.15124/d29e1e1f‐7fd2‐4cf1‐80e8‐220bca3194de

## Conflict of interest

The authors declare no conflict of interest.

## Supporting information

As a service to our authors and readers, this journal provides supporting information supplied by the authors. Such materials are peer reviewed and may be re‐organized for online delivery, but are not copy‐edited or typeset. Technical support issues arising from supporting information (other than missing files) should be addressed to the authors.

Supplementary
