# Supplementary material for: Work‐hardening Photopolymer from Renewable Photoactive 3,3’‐(2,5‐Furandiyl)bisacrylic Acid
Source: ChemSusChem. 2020 Jul 29;13(16):4140–50. doi: 10.1002/cssc.202000842 (PMC7496517; doi:10.1002/cssc.202000842)
Supplement: Supplementary file 1 — Supplementary [file CSSC-13-4140-s001.pdf]

ChemSusChem

Supporting Information

Work-hardening Photopolymer from Renewable Photoactive 3,3′-(2,5-Furandiyl)bisacrylic Acid

Yann Lie, Alessandro Pellis,* Ignacio Funes-Ardoiz, Diego Sampedro, Duncan J. Macquarrie, and Thomas J. Farmer*© 2020 The Authors. Published by Wiley-VCH Verlag GmbH & Co. KGaA. This is an open access article under the terms of the Creative Commons Attribution License, which permits use, distribution and reproduction in any medium, provided the original work is properly cited.

Author Contributions

Y.L. Investigation:Lead; Methodology:Lead; Validation:Lead; Writing - Original Draft:Lead; Writing - Review & Editing:Lead
A.P. Conceptualization:Equal; Formal analysis:Supporting; Investigation:Supporting; Methodology:Supporting; Validation:Supporting; Visualization:Equal; Writing - Original Draft:Supporting; Writing - Review & Editing:Supporting
I.F.-A. Conceptualization:Supporting; Data curation:Supporting; Formal analysis:Supporting; Investigation:Supporting; Visualization:Supporting; Writing - Review & Editing:Supporting
D.S. Conceptualization:Supporting; Formal analysis:Supporting; Investigation:Supporting; Methodology:Supporting; Visualization:Supporting; Writing - Review & Editing:Supporting
D.M. Funding acquisition:Equal; Supervision:Equal; Writing - Review & Editing:Equal
T.F. Conceptualization:Equal; Funding acquisition:Equal; Methodology:Equal; Supervision:Equal; Writing - Original Draft:Equal; Writing - Review & Editing:Equal

Materials and Methods

Additional methods and information..4

Supplementary Tables

Supplementary Table S1 Porosimetry analysis of MnO_2. *Post reaction, no calcination; **Post reaction, post calcination 10
Supplementary Table S2 Reusability of 88% MnO_2 after first reaction conversion measured by GC-FID...... 10
Supplementary Table S3 Recycling experiment with used MnO_2. *Calcination at 300 °C for 1 h prior to reaction........ 10
Supplementary Table S4 GPC results for the polymers synthesised using the chemo-catalytic protocols 11
Supplementary Table S5 TGA analysis of the 1-based polymers synthesised in DPE as the organic media... 11
Supplementary Table S6 DSC analysis of the 1-based polymers synthesised in DPE as the organic media ... 11
Supplementary Table S7 iCaLB-catalysed synthesis of 1-based PFAE in DPE as the organic media at 85 ºC and 20 mbar 12
Supplementary Table S8 MALDI analysis of the 1-based aromatic-aliphatic unsaturated polyesters synthesised in DPE........ 12
Supplementary Table S9 DSC analysis 1+ODO DPE 12
Supplementary Table S10 Vertical excitations of compound X and its dimer calculated with three different functionals. The three transitions with higher oscillator strength are shown (first 10 singlet excited states were calculated). 13

Supplementary Figures

Supplementary Figure S1 Semi-continuous oxidation of HMF to DFF with 88% MnO_2-packed HPLC column. A: Oxygen input (optional, 2 $mL.min^{-1}$); B: 1% HMF solution in TMO C: JASCO PU-980 HPLC pump D: Heating tape controller E: Heating tape F. MnO_2-packed HPLC column (4 mm internal diameter, 25 mm length) clamped in custom-made heating block G: DFF solution obtained at column exit. 14
Supplementary Figure S2 Custom made apparatus for UV-irradiation of samples 15
Supplementary Figure S3 Sketch of the 253 nm UV lamp used in reverse cycloaddition reaction trials. Rotor stand and clamp were omitted in the final version and samples were directly irradiated on a petri 16
Supplementary Figure S4 Powder XRD analysis a: 99% MnO_2; b: 88% MnO_2 c: 88% MnO_2 after first reaction dried at 80°C overnight in vacuum oven 17
Supplementary Figure S5 SEM-EDX 99% MnO_2........ 18
Supplementary Figure S6 EDX-SEM analysis of 88% MnO_2........ 19
Supplementary Figure S7 EDX-SEM images of 88% MnO_2 after first passage of HMF solution (dried in vacuum oven at 80 °C overnight) 20
Supplementary Figure S8 EDX-SEM of 88% MnO_2 after reaction and calcination at 300 °C for 1 h under air. 21
Supplementary Figure S9 ^{1}H NMR spectrum of DFF 22
Supplementary Figure S10 ^{13}C NMR spectrum of DFF 23
Supplementary Figure S11 ^{1}H NMR spectrum of **16** 24
Supplementary Figure S12 ^{13}C NMR spectrum of **16** 25
Supplementary Figure S13 ^{1}H NMR spectrum of monomer **1** 26
Supplementary Figure S14 ^{13}C NMR spectrum of monomer **1**........ 27
Supplementary Figure S15 1H-NMR analysis of the ODO-PFAE synthesised in diphenyl ether after the three purification steps. 28
Supplementary Figure S16 ^{13}C-NMR analysis of the ODO-PFAE synthesised in diphenyl ether after the three purification steps. 28

Supplementary Figure S17 Overlay of ^{1}H-NMR spectra for $CHCl_3$-soluble crude products obtained from chemo-catalysed polycondensations with 1,8-octanediol (ODO) and purified polymers obtained after enzymatically-catalysed polymerisation 29
Supplementary Figure S18 ^{1}H NMR spectrum of crude product obtained after 24 h UV-irradiation of monomer **1** (signals indicate monomer **1** and cycloadduct **2**) 30
Supplementary Figure S19 Deconvoluted ^{1}H NMR spectrum for the isolation of cycloadduct **2** signals after 24 h of irradiation of **1** 30
Supplementary Figure S20 ^{1}H NMR spectrum of crude product obtained after 72 h UV-irradiation of monomer **1** 31
Supplementary Figure S21 Overlay FT-IR spectra of UV-cured polymer ODO-PFAE after different irradiation time 31
Supplementary Figure S22 ^{13}C solid state NMR spectra of the cured (top) and uncured (bottom) ODO polymer. Dotted circles show the reduction of the alkene signal and appearance of the cyclobutane signal between 40-50 ppm 32
Supplementary Figure S23 TGA analysis of monomer **1** and of the polymer synthesised from **1** and 1,8-octanediol (ODO) in DPE using immobilized CaLB as the biocatalyst 33
Supplementary Figure S24 TGA analysis of the polymers synthesised (in duplicate) from **1** and 1,4-butanediol (BDO) in DPE using immobilized CaLB as the biocatalyst 34
Supplementary Figure S25 TGA analysis of the polymers synthesised (in duplicate) from **1** and 1,4-cyclohexanedimethanol in DPE using immobilized CaLB as the biocatalyst 35
Supplementary Figure S26 DSC thermogram (red, 1st heating, blue 1st cooling and orange 2nd heating cycle) of the PFAE containing BDO with DPE as the solvent 36
Supplementary Figure S27 DSC thermogram (red, 1st heating, blue 1st cooling and orange 2nd heating cycle) of the PFAE containing ODO as the diol with DPE as the solvent 37
Supplementary Figure S28 MALDI analysis of ODO-PFAE and assignment of the main repetitive unit 38
Supplementary Figure S29 MALDI analysis of BDO-PFAE and assignment of the main repetitive unit 39
Supplementary Figure S30 MALDI analysis of CHDM-PFAE and assignment of the main repetitive unit 40
Supplementary Figure S31 MALDI analysis of ODO-PFAE synthesised using zinc as the catalyst 41
Supplementary Figure S32 MALDI analysis of ODO-PFAE synthesised using zirconium as the catalyst 42
Supplementary Figure S33 Energy profile for cycloadduct formation through the triplet energy surface. Potential energies and free energies (in parentheses) in kcal mol^{-1} 43
Supplementary Figure S34 ^{1}H NMR of dilute irradiated solution of **1** after 50 h, integrated signal show the presence of *E-Z* isomer (0.01 M in $CDCl_3$ irradiation done in 1 cm quartz cuvette, signals between 2.0 and 3.0 ppm are due to the DMSO used as internal standard) 43
Supplementary Figure S35 Overlay of FT-IR spectrum of photoinduced reversed cycloaddition trials (monomer **1** cured for >72 h was used and irradiated at indicated times) a: Non-standardised data b: Standardised data using excel built-in function 44
Supplementary Figure S36 Proposed mechanism for the transesterification catalysed by the serine hydrolase enzyme CaLB 45
Supplementary Figure S37 ^{1}H-NMR spectrum of blank media for extraction studies of UV-cured ODO-PFAE polymer strips, showing THF-d_8 and dimethyl maleate was used as an external standard. A: full spectrum range, B: expansion of 4.5-3.5 ppm 46
Supplementary Figure S38 ^{1}H-NMR spectrum of recovered solvent (THF-d_8) from extraction studies of UV-cured ODO-PFAE polymer strips (9.3 mg), showing and dimethyl maleate was used as a standard (IS). A: full spectrum range and calculation of polymer extracted from strip using the CH_2 signal at 4.05 ppm, CRU of

polymer assumed = 318 g/mol, B: expansion of 4.5-3.5 ppm showing signal and 4.05 ppm (see Figure S37 B for comparison) 47
Supplementary Figure S39 ^{1}H-NMR spectrum of recovered solvent ($CDCl_3$) from extraction studies of UV-cured ODO-PFAE polymer strips (10.8 mg), showing and dimethyl maleate was used as a standard (IS). A: full spectrum range and calculation of polymer extracted from strip using the CH_2 signal at 4.13 ppm, CRU of polymer assumed = 318 g/mol, B: expansion of 4.5-3.5 ppm 48

Supplementary references

Supplementary references 57

Materials and methods

Chemicals, enzymes and UV-curing apparatus

5-(hydroxymethyl)furfural (HMF) was purchased from Fluorochem, manganese dioxide 88%, MnO_2 99% (dried at 120 °C overnight in a vacuum oven and stored in a vacuum desiccator) and 1,8-octanediol (ODO) were purchased from Acros Organics. Pyridine and piperidine were purchased from Alfa Aesar, 98% H_2SO_4, 37% HCl, K_2CO_3, NaCl, $MgSO_4$, chloroform and methanol were purchased from Fischer Scientific. 3Å molecular sieves (activated at 350 °C overnight stored in 80 °C oven) were bought from Honeywell Fluka. Candida antarctica lipase B (CaLB) immobilized onto acrylic resin (iCaLB, product code: L4777), 1,4-butanediol (BDO), diphenyl ether (DPE), Zr(IV) isopropoxide 2-propanol complex, Zn(II) acetate, Ti(IV) *tert*-butoxide, malonic acid, 2-propanol, $CDCl_3$ and all other chemicals and solvents were purchased from Sigma-Aldrich and used as received if not otherwise specified. 2,2,5,5-tetramethyloxolane (TMO) was prepared as previously described and stored over 3 Å molecular sieves.[1] iCaLB was lyophilized for 48 h and stored in a desiccator before use. The hydrolytic activity of this iCaLB formulation was previously determined using the tributyrin assay, resulting in an activity of 2200 U $gdry^{-1}$, further details on the data and protocal for this assay is available from the study by Pellis *et al.*.[2]

UV-lamps used for curing were custom-built by the electronic workshop of the University of York. The 365 nm UV LED gen2 emitter, 1250 mW (max output), LZ1-00UV00 was purchased from LED engine and the 5 W, 253 nm fluorescent lamp (PLS5/TUV 2 Pin 5W Germicidal UVC) from Lamp Specs website. The 22 mm diameter biconvex quartz lens used to collimate the 235 nm beam was purchased from UQG optics.

Synthesis of diformylfuran (DFF, **15**):

A spent HPLC column (25 mm length, 4 mm internal diameter) was emptied, cleaned then re-packed with 88% MnO_2 (6.2 g) with vacuum connected to one end of the column to ensure effective packing. The column was then connected to a JASCO HPLC pump PU 980 and clamped in a custom-made heating block and heated to 75 ºC. Heating tape was applied to the tubing at the exit of the column to avoid clogging of the capillaries (ESI, Figure S1). Thereafter, neat TMO preheated to 75 ºC was passed through the column at a flow rate of 1 $mL.min^{-1}$. Once the first drop of TMO exited the column, a 1% w/v HMF solution in TMO (1 g HMF, 7.9 mmol, in 100 mL TMO) was preheated to 75 ºC and then passed through the system. The solution at the exit of the column was evaporated to afford 2,5-diformylfuran (DFF) as white solid (0.771 g, 6.2 mmol, 78% yield). The evaporated TMO was recovered and reused after distillation through a Vigreux column and drying over $MgSO_4$. ^1H NMR (400 MHz, [$CDCl_3$]): δ (ppm) 9.86 (s,2H, CHO-fur-CHO), 7.33 (s, 2H, CHO-CO=CH-CH=CO-CHO, furan). ^{13}C NMR (101 MHz, [$CDCl_3$]): δ (ppm) 179.33 (CHO-fur-CHO), 154.29 (CHO-CO=CH-CH=CO-CHO, furan), 119.34 (t, CHO-CO=CH-CH=CO-CHO, furan). Data are in accordance with previously reported chemical shifts.[3]

Synthesis (2E,2'E)-3,3'-(2,5-furandiyl)bisacrylic acid, **16**:

An oven dried 2-necked 250 mL round bottom flask equipped with a waterless condenser was charged with 2,5-diformylfuran (12. 566 g, 101 mmol), malonic acid (42.146 g, 405 mmol), 150 mL of anhydrous pyridine (stored under 3 Å molecular sieves) and a magnetic stirrer flea. After complete dissolution, piperidine (1.724 g, 20.2 mmol, 0.2 eq.) was added and the system was heated to 85 °C overnight (16 h). Thereafter, the reaction was brought to reflux for 2h. After cooling to room temperature, the solution was poured in a 500 mL beaker and neutralised with careful addition of 37% conc. HCl (150 mL) and 1 M HCl (300 mL) until the pH dropped under 1. The reaction mixture

was place on an ice bath and the resulting precipitate was collected via vacuum filtration over fritted funnel. The beige solid further was washed with cold 1 M HCl and dried at 80 ºC in a vacuum oven overnight to afford of 2,5-furan diacrylic acid (17.25 g, 82.9 mmol, 82% yield). ^{1}H-NMR (400 MHz, [DMSO-d_6]): δ (ppm) 7.34 (d, J = 15.8 Hz, =CO-CH=CH-COOH *2), 6.97 (s, -CO=CH-CH=CO-, furan), 6.34 (d, J = 15.8 Hz, =CO-CH=CH-COOH *2). 13C-NMR (101 MHz; [DMSO-d_6]): δ (ppm) 167.7 (=CO-CH=CH-COOH *2), 152.6 (=CO-CH=CH-COOH *2, furan), 130.5 (=CO-CH=CH-COOH *2), 118.9 (=CO-CH=CH-COOH *2), 118.1 (-CO=CH-CH=CO-, furan). Chemical shifts are in accordance with previously reported values.[3] ESI-MS: $C_{10}H_8NaO_5$, theoretical: 231.0264 [M^+], measured: 231.0271 [M^+]. Melting point: Decomposes >200 °C.

<u>Synthesis of (2E,2'E)-3,3'-(2,5-furandiyl)bisacrylic acid dimethyl ester, **1**:</u>

An oven-dried 250 mL round bottom flask equipped with a waterless condenser was charged with -2,5-furan diacrylic acid (8.144 g, 39.1 mmol) and 80 mL MeOH. Consequently, NaCl (0.984 g, 16.8 mmol, 0.43 eq.) was added to decrease the solubility of the diester in the methanolic solution and 98% H_2SO_4 (210 µL, 3.91 mmol, 0.1 eq.) was added. The suspension was refluxed for 7h and left to cool with stirring. The solution was further cooled with stirring to 0 °C with an ice bath and the crystalline material was collected by vacuum filtration. Recrystallisation from 2-propanol followed by drying under high–vacuum afforded monomer 1 as pale brown shiny flakes (7.377 g, 31.2 mmol, 82% yield). This monomer was protected from light as much as possible until further use. ^{1}H-NMR (400 MHz, [$CDCl_3$]): δ (ppm) 7.40 (2H, d, J = 15.7 Hz, =CO-CH=CH-COOMe *2), 6.65 (2H, s, -CO=CH-CH=CO-, furan), 6.43 (2H, d, J = 15.8 Hz, =CO-CH=CH-COOMe *2), 3.80 (s, CH3-OOC-CH=CH-). 13C NMR (101 MHz; [$CDCl_3$]): δ (ppm) 167.2 (=CO-CH=CH-COOMe *2), 152.5 (=CO-CH=CH-COOMe *2, furan), 130.4 (=CO-CH=CH-COOMe *2), 117.6 (=CO-CH=CH-COOMe *2), 116.9 (-CO=CH-CH=CO-, furan), 52.0 (=CO-CH=CH-COO-CH3*2). Chemical shifts are in accordance with previously reported values.[3] ESI-MS: $C_{12}H_{12}NaO_5$, theoretical (m/z): 259.0577 [M^+] measured (m/z): 259.0759 [M^+]. Melting point: 153.7 – 155.9 °C. (lit. 152-153 °C)[4]

<u>Chemo-catalytic polycondensation of **1** with 1,8-octanediol:</u>

In a custom-made polymerisation flask were added consecutively, the monomer 1 (1 g, 4.23 mmol), 1,8-octane diol (1.24 g, 8.47 mmol) and 5 mol% of the catalyst (K_2CO_3, Zr(IV) isopropoxide isopropanol complex, Zn(II) acetate or Ti(IV) *tert*-butoxide e.g. for Ti(IV) *tert*-butoxide: 72.04 mg, 35 mol% respective to titanium, 0.05 eq.) with a large egg shaped rare earth magnetic stirring bar. The vessel was surmounted with a short path distillation arm to continuously remove the produced methanol, connected to a Schlenk line and the system was purged and filled with Ar or N_2 3 times before heating under Ar or N_2 at 95 ºC overnight. High vacuum was then applied whilst maintaining the heating, for 4 hours to remove any excess diol and the crude material was analysed by GPC.

<u>Enzymatic polymerizations in high boiling organic media</u>

Based on previously published procedure used for alternative monomers enzymatic polycondensation reactions were carried out as follows.[5] 37 8 mmol of diester (0.2 M) and 8 mmol of the diol (0.2 M) (diester:diol ratio= 1:1) were added together with 4 mL of diphenyl ether (DPE) in a 25 mL round bottom flask. The mixture was then stirred at 85 ºC until complete dissolution of the monomers in the solvent. 10% w w^{-1} (calculated on the total amount of the monomers) of iCaLB was then added and the reaction was run for 6 h at 1000 mbar. The reaction system was covered with aluminium foil to avoid light-induced side reactions of the components. A vacuum of 20 mbar was subsequently applied for an additional 90 h while maintaining the reaction temperature at 85 ºC. Warm chloroform was added to the reaction mixture to solubilize the polymer product and the biocatalyst was filtered off. The chloroform was then removed under vacuum. The polymer-DPE

mixture was subsequently crashed out in ice-cold methanol achieving precipitation of the products. Three methanol triturations were subsequently performed in order to remove the residual DPE. The reactions led to a light-yellow powdery polymerization product. All reactions were conducted in duplicates.

Enzymatic polymerizations in low boiling organic media

Enzymatic polycondensation reactions were conducted in similar manner to that previously described for other polyesters.46 Briefly, $8x10^{-4}$ mol of diester (0.2 M) and $8x10^{-4}$ mol of the aliphatic diol (0.2 M) (diester:diol ratio= 1:1) were added together with 4 mL of TMO as a low boiling, green, organic solvent in a 100-mL round bottom flask. The mixture was then stirred at 85 ºC until complete dissolution of the monomers in the solvent. 10% w/w (calculated on the total amount of the monomers) of iCaLB was then added and the reaction was run for 6 h at 1000 mbar. The reaction system was covered with aluminium foil to avoid light-induced side reactions of the components. A vacuum of 300 mbar was subsequently applied for an additional 90 h while maintaining the reaction temperature at 85 ºC. At the end of the reaction, the biocatalyst was filtered off and the TMO was then removed under vacuum. The reactions led to yellow powdery polymerization products.

ODO-PFAE film casting:

The synthesized polymer, ODO-PFAE was dissolved in chloroform (10 mg mL^{-1}) and the solution casted on a Kapton polyimide film placed inside a glass petri dish. The solvent was slowly removed at 21 °C keeping the casting system in the dark. The obtained thin film (thickness of 50 μm) was then peeled off the polyimide film and mechanically tested and cured as described below.

UV-irradiation of synthesised material

The beam of a custom-made LED torch or fluorescent lamp (ESI Figure S2 and S3) maximum emission at 365 nm, 1250 mW maximum flux output for the LED and 253 nm, 5W for the fluorescent lamp) was collimated with a biconvex plastic lens (for irradiation at 365 nm) and a biconvex quartz lens (for irradiation at 253 nm) on the sample layer (typically 100 mg neat for monomer 1 and ODO-PFAE 2.5 cm long, 0.5 cm wide strips for film, <1 mm thick, distance from lens ~5 cm for 365 nm lamp, <2 cm for 253 nm lamp) placed on a glass petri dish. The reaction was monitored by FT-IR spectroscopy and mixing with a spatula (for powdery/crystalline) or change of irradiated faces (for film strips) was done every 24 hours to assure homogeneous curing. Typically, UV-curing of a sample was stopped after 72 hours of continuous irradiation unless specified otherwise.

Solubility testing of the cured material

The cured film strips were accurately weighed (9-10 mg) and place in either THF-d_8 or $CDCl_3$ (800 μL of each) and put on an automatic roller for 1 hour (RT), after which the strip was removed and NMR spectrum of the extract recorded following addition of dimethyl maleate (2 μL) as a standard. Comparisons of integration of signals possibly of extracted polymer (CH_2s of ester bond) with the standard allowed for determination of extent of extraction (see Figures S37-39). Separately, the recovered strips from above were dried thoroughly under high-vacuum and weighed again to assess via mass balance what proportion of the polymer was extracted into solution, no mass change observed using THF, while for $CDCl_3$ the mass of strip reduced from 10.8 to 10.7 mg.

Nuclear Magnetic Resonance (NMR) Spectroscopy

^1H and ^{13}C-NMR spectroscopy analysis were performed on a JEOL JNM-ECS400A spectrometer at a frequency of 400 MHz for ^1H and 100 MHz for ^{13}C. $CDCl_3$ was used as solvent if not otherwise specified.

Gel Permeation Chromatography (GPC)

Samples were dissolved in $CHCl_3$ and filtered through a cotton filter prior to passing into a HPLC vial. Gel permeation chromatography was carried out at 30 °C on an Agilent Technologies HPLC System (Agilent Technologies 1260 Infinity) connected to a 17369 6.0 mm ID × 40 mm L HHR-H, 5 µm Guard column and a 18055 7.8 mm ID × 300 mm L GMHHR-N, 5 µm TSKgel liquid chromatography column (Tosoh Bioscience, Tessenderlo, Belgium) using 1 mL min^{-1} $CHCl_3$ as mobile phase. An Agilent Technologies G1362A refractive index detector was employed for detection. The molecular weights of the polymers were calculated using linear polystyrene calibration standards 250-70000 Da (Sigma-Aldrich).

Matrix Assisted Laser Desorption Ionization (MALDI)

MALDI-TOF MS analysis were carried out by using a Bruker Solarix-XR FTICR mass spectrometer and the relative software package for the acquisition and the processing of the data. An acceleration voltage of 25 kV, using DCTB as matrix and KTFA as ionization agent were used. 10 µL of sample were mixed with 10 µL of matrix solution (40 mg mL^{-1} DCTB in CHCl3) and 3 µL of KTFA (5 mg mL^{-1}). 0.3 µL of the mixture were applied on the plate and the measurement was conducted in positive mode with the detector set in reflector mode.

Differential Scanning Calorimetry (DSC)

DSC experiments were performed on a TA Instruments Q2000 DSC under an inert gas atmosphere (N_2). Heating and cooling rates were set to 5 °C/min over the T range of -60-200 °C. Sample mass was of between 5-10 mg for all measured samples. The T_g values were calculated from the second heating scan.

Thermogravimetric analysis (TGA)

TGA was performed on a PL Thermal Sciences STA 625 thermal analyser. ~10 mg of accurately weighed sample in an aluminium sample cup was placed into the furnace with a N_2 flow of 100 mL min^{-1} and heated from room temperature to 625 °C at a heating rate of 10 °C min^{-1}. From the TGA profiles the temperatures at 10% and 50% mass loss (T_d10 and T_d50) were subsequently determined.

Fourier Transformation Infrared Spectroscopy

Fourier Transformation Infrared Spectroscopy (FT-IR) analysis of the synthesized polymers was performed on a PerkinElmer 400 spectrometer using the attenuation total reflectance setting. The same pressure was applied on the outer surface of all analysed samples. A number of 16 scans were recorded using a 1 cm^{-1} resolution. All spectra were processed using the automated baseline correction and the data auto tune functions.

Computational Details

All the calculations were carried out in Gaussian16 (A.03) program package.[6] Optimizations and frequency calculations were performed without symmetry restrictions at wB97xD/6-31+G(d,p) level

due to the good agreement with the experimental UV-VIS absorption of monomer 1.[7] Other functionals were also tested (ESI Table.S10). Solvation was introduced by the CPCM implicit solvent method (chloroform).[8] All the stationary points were characterized as minima or transition state (0 or 1 imaginary frequency) in the corresponding electronic state (ground or excited state) and thermochemistry corrections at standard conditions were added to obtain the corresponding free energies. Both potential energies and free energies (in parentheses) are included in the manuscript in kcal/mol.

Supplementary methods

Additional methods and information

MestReNova (version, 14.0.0, MestreLab Reasearch) was used for NMR spectra processing. Deconvolution was carried out using the software Global Spectral Deconvolution as described here: https://resources.mestrelab.com/gsd

OriginLab2019 (V9.6.5.169, 2019b) was used to compute the full width at height maximum (FWHM) used to calculate the domain frame by the Scherrer equation:

$$\alpha = \frac{K\lambda}{FWHM\cos(\frac{2\theta}{2})}$$

Where α is the domain frame or crystallite size (in nm), K is the Scherrer constant taken as 0.9, λ the wavelength used for analysis, here 0.15406 nm, FWMH, the full width at height maximum and θ the peak position. The asymptotic exponential fitted curved for the conversion followed by FT-IR spectroscopy was also computed with OriginLab using the automatic aymptot1 exponential model available in the software.

XRD was performed using a Bruker AXS D8 Advance controlled by XRD Commander software, with scan type set at locked coupled, operating voltage of 40 kV (current, 40 mA), scan speed of 2 sec/step and the scan scope from 0 θ to 90 θ.

Single crystal XRD were recorded on an Oxford Diffraction SuperNova apparatus equipped with dual Mo & Cu sources and structure was resolved by Rachel R. Parker.

GC-FID was recorded on an Agilent GC7890B equipped with Rxi-5HT column The GC method was as followed: initial temperature 50 ºC, holding 0min, Ramp rate 30 ºC/min, Temperature final: 300 ºC, hold 5 min, split ratio 5:1, injector temperature 300 ºC. Blanks containing DCM and TMO was run prior analysis of the HMF-DFF mixture

Porosimetry analyses were performed using a Micromeritics ASAP 2010. Samples were degased for at least 6 h at 130 °C prior to analysis. The Brunauer-Emmett-Teller (BET) theory was used to determine the surface area, and data processed using the MicroActive software provided with the instrument.

EDX-SEM data were recorded on a JEOL 7800F Prime SEM, equipped with a Schottky (field-assisted) thermionic emitter.

Supplementary Tables

Supplementary Table S1 Porosimetry analysis of MnO_2. *Post reaction, no calcination; **Post reaction, post calcination

	BET Surface Area (m^2/g)	Adsorption average pore width (4V/A by BET, nm)	Desorption average pore width (4V/A by BET, nm)	BJH Adsorption average pore width (4V/A, nm)	BJH Desorption average pore width (4V/A, nm)
**MnO_2 99%**	19	4.4	4.3	5.3	5.1
**MnO_2 88%**	108	7.7	7.6	7.8	6.6
**MnO_2 88%***	74	8.83	8.7	10.6	8.2
**MnO_2 88%****	77	11.6	11.4	10.9	9.7

Supplementary Table S2 Reusability of 88% MnO_2 after first reaction conversion measured by GC-FID

Passage	AreaDFF (RT = 3.71 min)	%AreaDFF	Area HMF (RT = 2.95 min)	%AreaHMF
1	345726.4	53%	304786	47%
2	172926.9	67%	86764.5	33%
3	328513.2	72%	124952.4	28%
4	226206.1	82%	49939.4	18%
5	208220.6	84%	38340.2	16%
6	285530.7	87%	41563.4	13%

Supplementary Table S3 Recycling experiment with used MnO_2. *Calcination at 300 °C for 1h prior to reaction

Passage	AreaDFF	%AreaDFF	AreaHMF	%AreaHMF
**1**	40.8	12%	285.7	88%
**2**	46.3	14%	287.2	86%
**3**	36.1	23%	119.5	77%
**4**	43.0	22%	149.9	78%
**5**	59.2	22%	207.8	78%
**6***	38.3	59%	26.9	41%

Supplementary Table S4 GPC results for the polymers synthesised using the chemo-catalytic protocols

Catalyst	Formula	M_n [Da]	M_w [Da]	Đ
**Potassium carbonate**	K_2CO_3	200	400	2.77
**Zinc(II) acetate**	$Zn(CH_3COO)_2$	100	400	3.42
**Zirconium(IV) *iso*-propoxide**	$Zr(OCH(CH_3)_2)_4$	>100	100	1.33
**Titanium(IV) *tert*-butoxide**	$Ti[OC(CH_3)_3]_4$	1200	2400	1.96

Supplementary Table S5 TGA analysis of the **1**-based polymers synthesised in DPE as the organic media

Diester	Diol	T_d5 [%]	T_d10 [%]	T_d50 [%]	Residue at 625 °C
**1**		192	211	267	3
**1**	**BDO**	356	376	435	32
		349	377	438	34
	**ODO**	382	389	434	13
		385	396	438	29
	**ODO**[a]	270	379	422	10
	**CHDM**	376	392	431	23
		375	394	430	22

[a]reaction conducted using double the reagent's concentration (0.4 M instead of 0.2 M)

Supplementary Table S6 DSC analysis of the 1-based polymers synthesised in DPE as the organic media

Diester	Diol	T_{m1} [°C][1]	T_g [°C][2]			ΔCp J/(g °C)[2]
**1**	**BDO**	101	35	47	39	0.17
		100	31	42	37	0.16
	**ODO**	59	16	23	19	0.18
		61	23	30	28	0.19
	**ODO**[a]	54	-3	2	-2	0.28
	**CHDM**	108	78	88	84	0.17
		107	75	81	80	0.05

[a]reaction conducted using double the reagent's concentration (0.4 M instead of 0.2 M)

Supplementary Table S7 iCaLB-catalysed synthesis of **1**-based PFAE in DPE as the organic media at 85 ºC and 20 mbar

	^{1}H-NMR		Isolated yield [%][3]	GPC		
**Diol**	**Monomers conversion [%][1]**	**Monomers conversion [%][2]**		**M_n [Da]**	**M_w [Da]**	**Đ**
**BDO**	85	82	74	1300	3300	2.5
	86	86	75	1300	3300	2.5
**ODO**	97	97	89	3600	25700	5.9
	97	97	91	3500	22100	6.3
**ODO[a]**	88	89	71	3000	6600	2.2
**CHDM**	94	92	82	1600	5000	3.2
	93	92	77	1800	5100	2.8

[a] reaction conducted using double the reagent's concentration (0.4 M instead of 0.2 M).
[1] calculated from ^{1}H-NMR based on the diol component.
[2] calculated from ^{1}H-NMR based on the diester component.
[3] calculated after MeOH precipitation and three washing steps.

Supplementary Table S8 MALDI analysis of the **1**-based aromatic-aliphatic unsaturated polyesters synthesised in DPE.

comment.

**Polymer**		**End groups**			
**Ester**	**Diol**	**Ester/diol**	**Cyclic**	**Ester/ester**	**Diol/diol**
**1**	BDO	+++	+	+-	+-
	ODO	+++	+	+-	+-
	CHDM	+++	+	+	+

Supplementary Table S9 DSC analysis **1**+ODO DPE

**Heating cycle**	**T_g [°C][2]**			**ΔCp J/(g °C)[2]**
2nd	23	30	28	0.19
3rd	25	33	29	0.18
4th	26	34	31	0.15
5th	28	36	33	0.13
6th	29	37	34	0.13

Supplementary Table S10 Vertical excitations of compound **1** and its dimer **1_{Dim}** calculated with three different functionals. The three transitions with higher oscillator strength are shown (first 10 singlet excited states were calculated).

Functional\Compound	**1**	**1_{Dim}**
ωB97xD	S1: 349 nm; f = 0.819<br>S4: 247 nm; f = 0.535<br>S5: 221 nm; f = 0.451	S2: 345 nm; f = 1.494<br>S3: 312 nm; f = 0.061<br>S10: 246 nm; f = 0.769
B3LYP-D3(BJ)	S1: 392 nm; f = 0.878<br>S5: 248 nm; f = 0.586<br>S8: 232 nm; f = 0.315	S4: 382 nm; f = 1.652<br>S6: 303 nm; f = 0.018<br>S7: 295 nm; f = 0.096
CAMB3LYP-D3(BJ)	S1: 352 nm; f = 0.813<br>S4: 250 nm; f = 0.463<br>S5: 223 nm; f = 0.531	S2: 346 nm; f = 1.547<br>S8: 250 nm; f = 0.067<br>S10: 249 nm; f = 0.573
Experimental value	352 nm (π-π*); 245 nm (n- π*)	

Both ωB97xD and CAMB3LYP-D3(BJ) yield accurate results for this system. The ωB97xD functional was selected for this study for its reduced computational cost.

Supplementary Figures

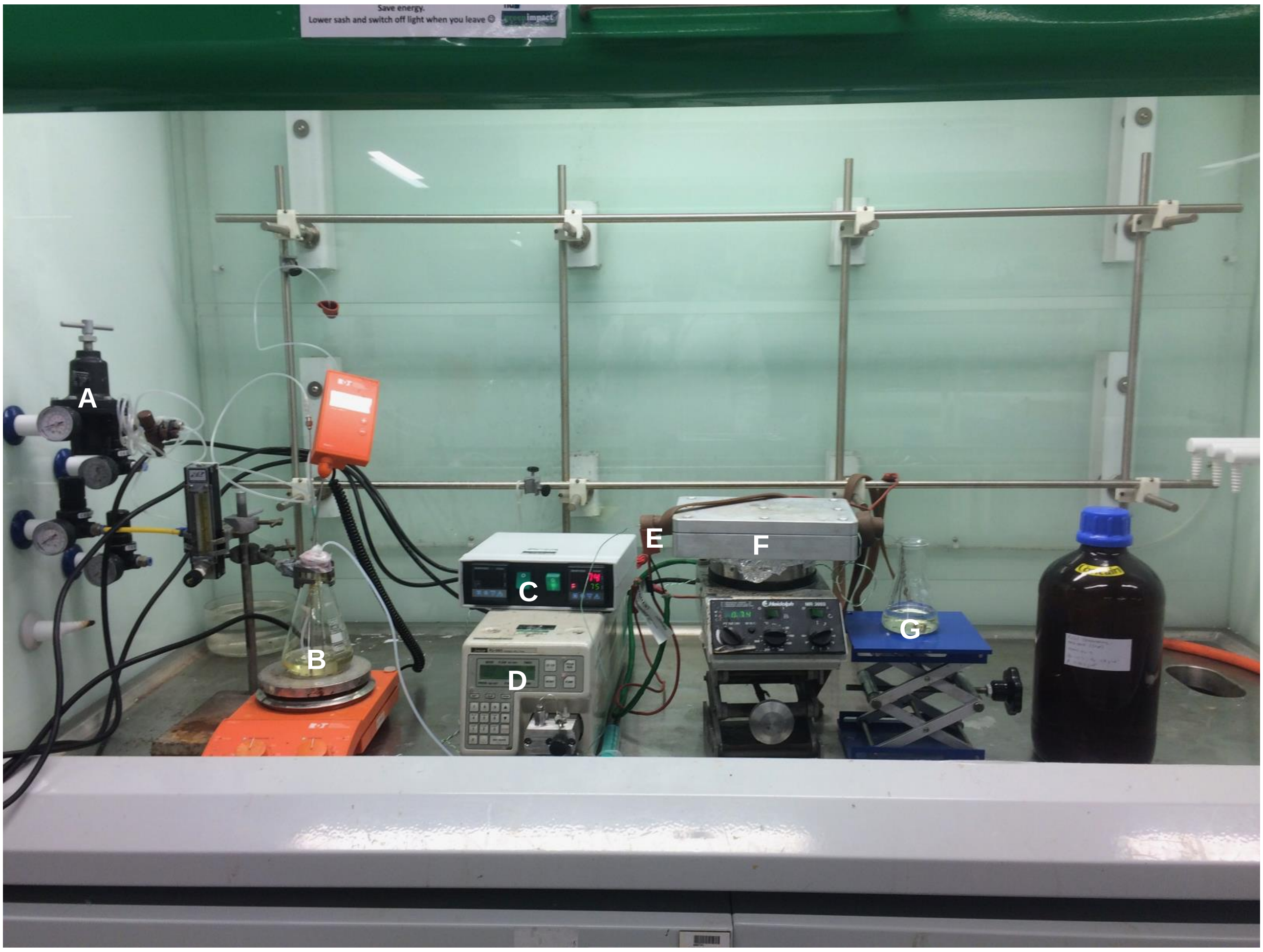

Supplementary Figure S1 Semi-continuous oxidation of HMF to DFF with 88% MnO_2-packed HPLC column. **A**: Oxygen input (optional, 2 $mL.min^{-1}$); **B**: 1% HMF solution in TMO **C**: JASCO PU-980 HPLC pump **D**: Heating tape controller **E**: Heating tape **F**. MnO_2-packed HPLC column (4 mm internal diameter, 25 mm length) clamped in custom-made heating block **G**: DFF solution obtained at column exit.

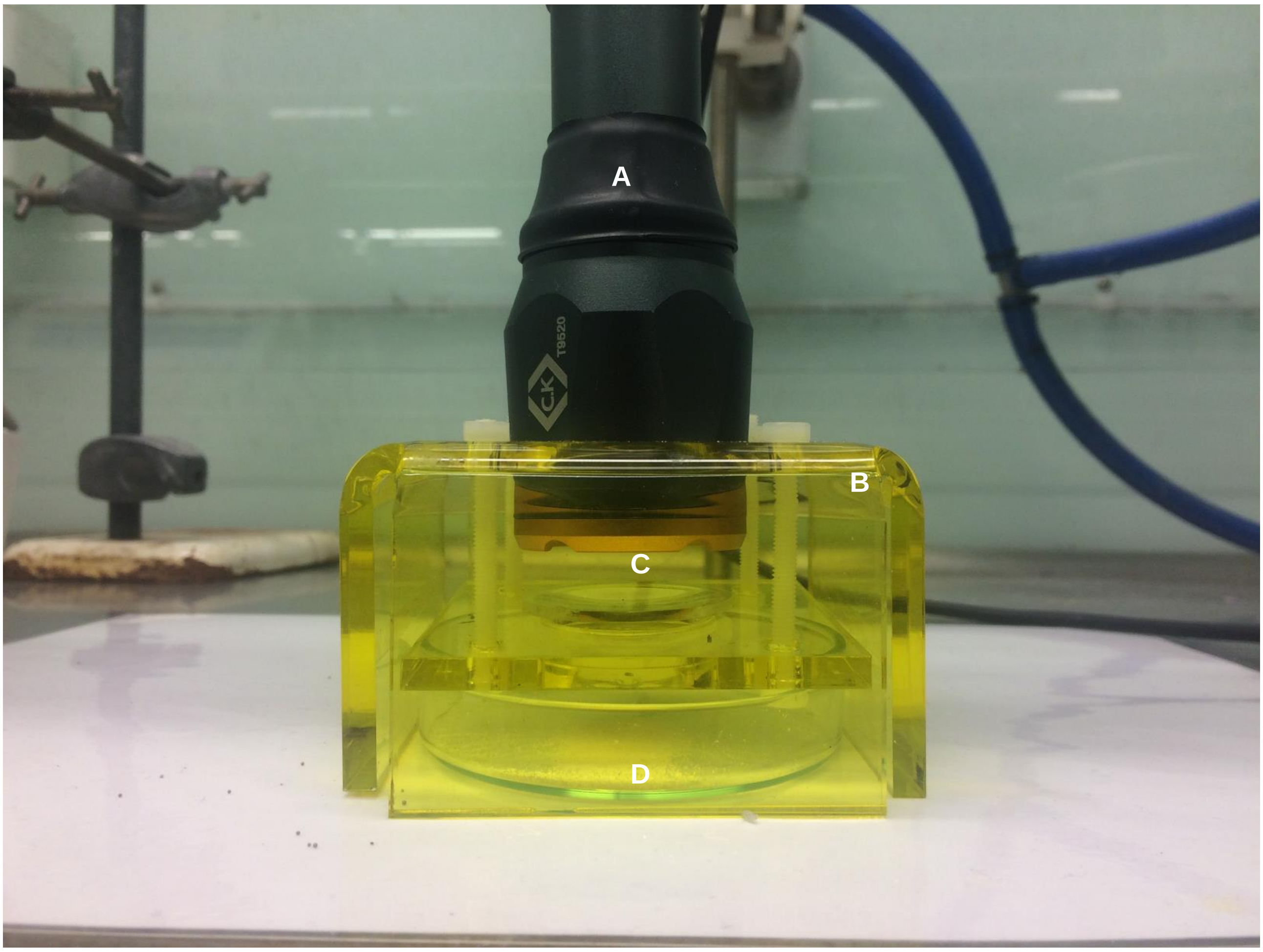

Supplementary Figure S2 Custom made apparatus for UV-irradiation of samples

A: LED torch (maximum emission at 365 nm, 1250 mW flux output); B: Plastic casing coated with UV-protecting filter; C: Quartz lens; D: Irradiated sample in petri dish.

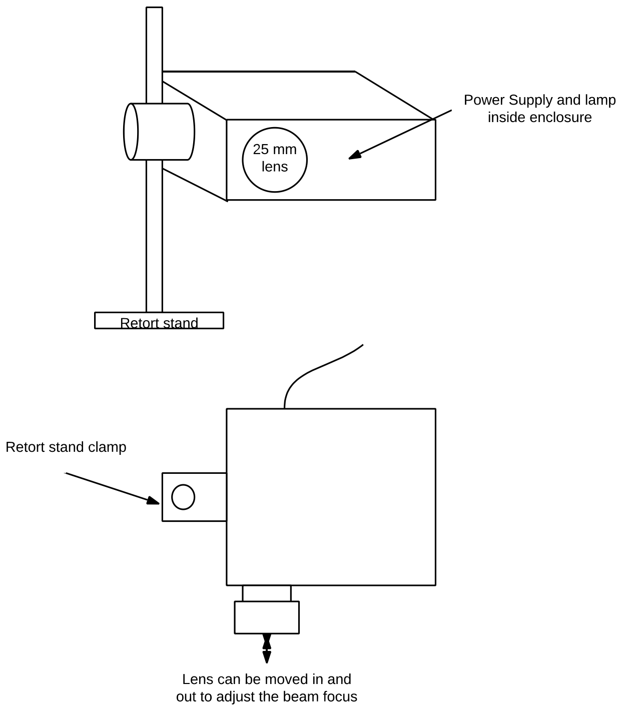

Supplementary Figure S3 Sketch of the 253 nm UV lamp used in reverse cycloaddition reaction trials. Rotor stand and clamp were omitted in the final version and samples were directly irradiated on a petri

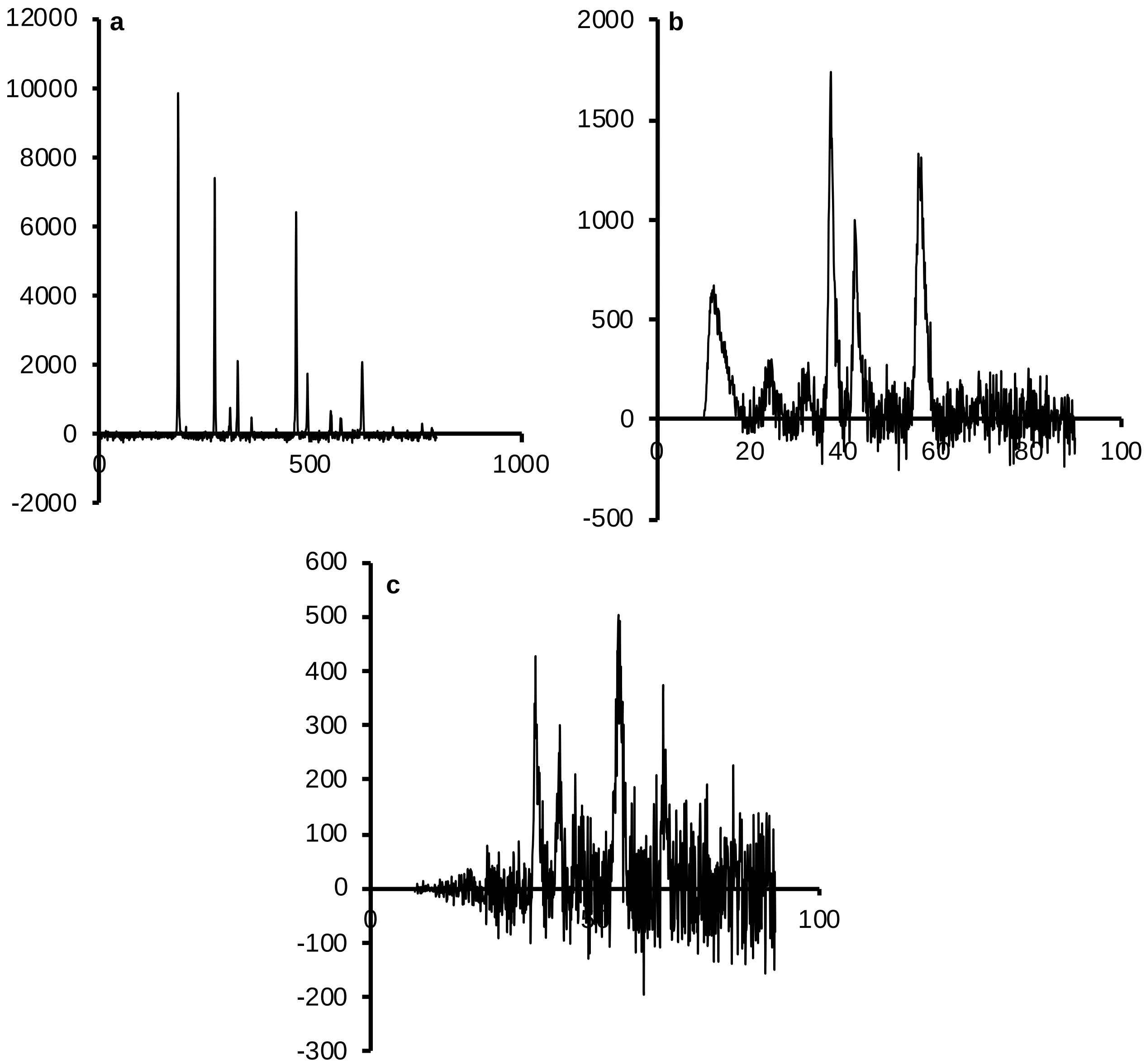

Supplementary Figure S4 Powder XRD analysis **a**: 99% MnO_2; **b**: 88% MnO_2 **c**: 88% MnO_2 after first reaction dried at 80°C overnight in vacuum oven

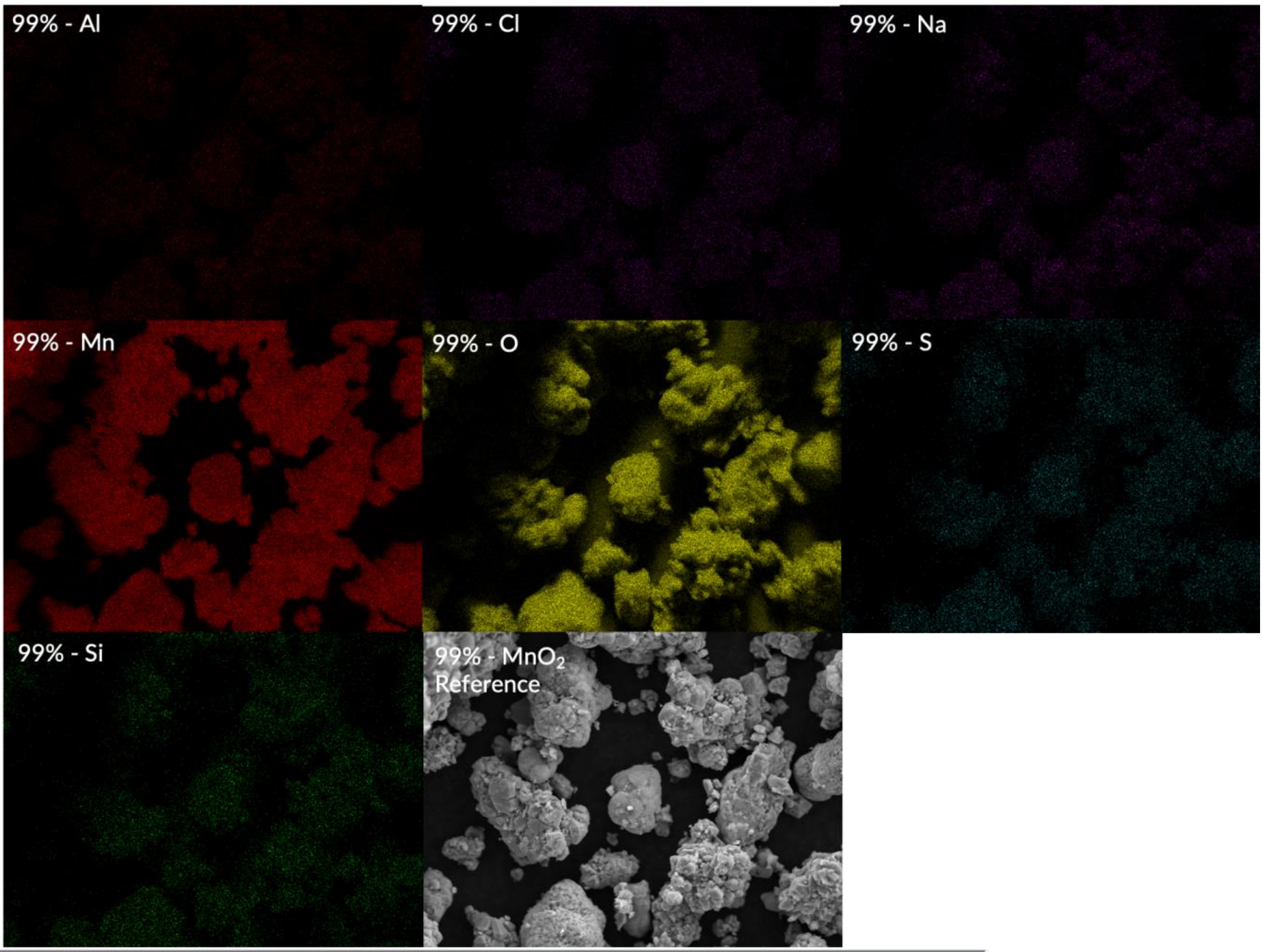

Element	expst(23)		
	Net Counts	Weight %	Atom %
O K	507936	14.61	36.77
Na K	2892	0.19	0.33
Al K	15846	0.42	0.62
Si K	2739	0.06	0.09
S K	4699	0.10	0.13
Cl K	3316	0.08	0.09
Mn K	1193876	84.55	61.98
		100.00	100.00

Supplementary Figure S5 SEM-EDX 99% MnO_2

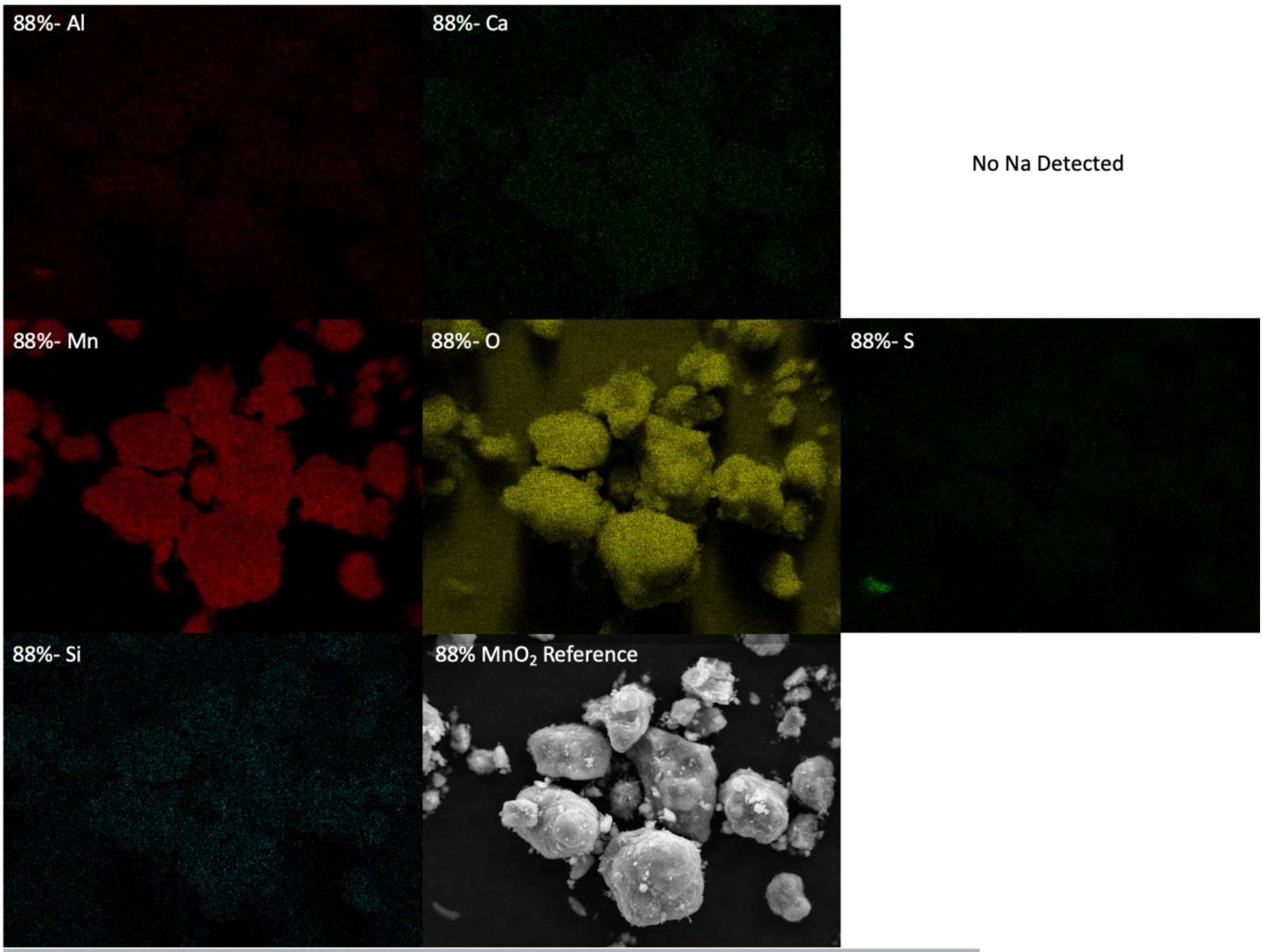

Element	expst(21)		
	Net Counts	Weight %	Atom %
Mn K	739251	74.57	46.75
Ca K	3918	0.16	0.13
S K	10998	0.33	0.35
Si K	6382	0.20	0.24
Al K	14238	0.51	0.65
Na K	5467	0.49	0.73
O K	599381	23.75	51.13
		100.00	100.00

Supplementary Figure S6 EDX-SEM analysis of 88% MnO_2

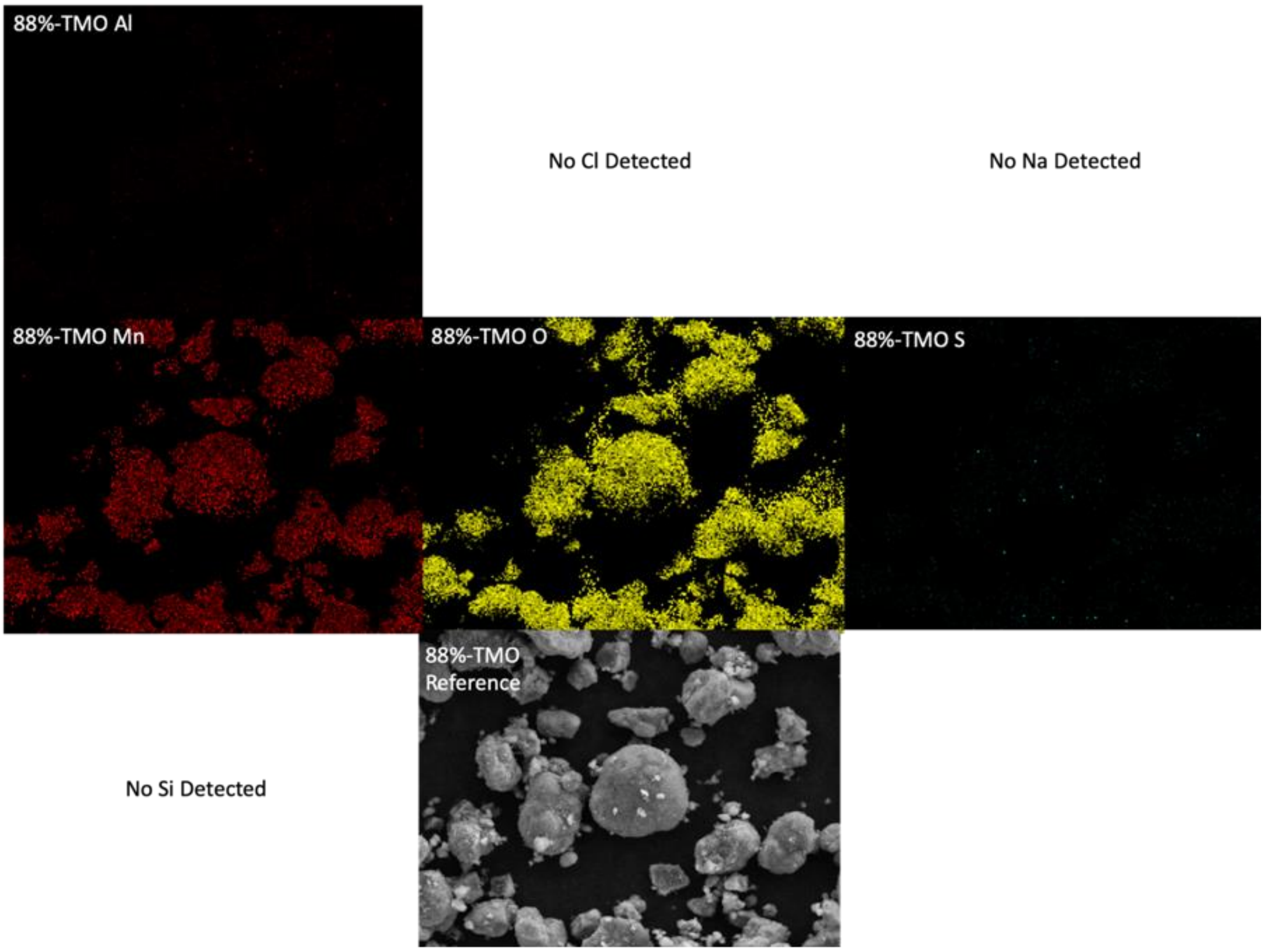

Element	Base(1)		
	Net Counts	Weight %	Atom %
O K	44221	21.40	48.07
Al K	1205	0.53	0.71
S K	856	0.31	0.35
Mn K	63229	77.75	50.87
		100.00	100.00

Supplementary Figure S7 EDX-SEM images of 88% MnO_2 after first passage of HMF solution (dried in vacuum oven at 80 °C overnight)

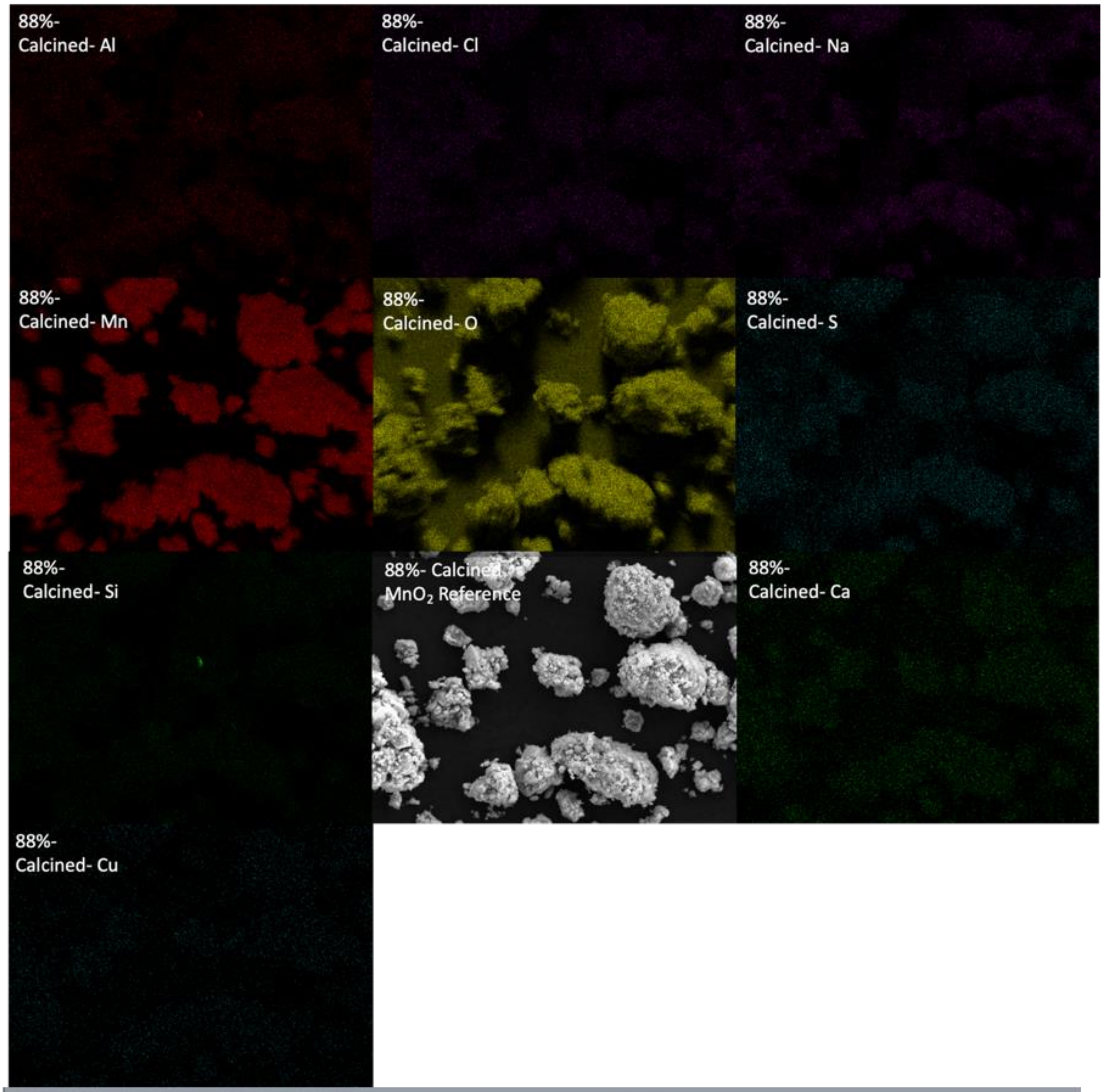

Element	expst(22)		
	Net Counts	Weight %	Atom %
O K	552833	18.96	44.14
Na K	4374	0.34	0.54
Al K	13348	0.41	0.57
Si K	6785	0.18	0.24
S K	11438	0.29	0.34
Cl K	1975	0.05	0.06
Ca K	5244	0.18	0.16
Mn K	933938	79.40	53.84
Cu K	900	0.19	0.11
		100.00	100.00

Supplementary Figure S8 EDX-SEM of 88% MnO_2 after reaction and calcination at 300 °C for 1 h under air.

Supplementary information characterisation of synthesised compounds

2,5-diformylfuran (DFF):

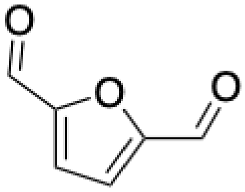

^{1}H NMR (400 MHz, [$CDCl_3$]): δ (ppm) 9.86 (s,2H, CHO-fur-CHO), 7.33 (s, 2H, CHO-CO=CH-CH=CO-CHO, furan)

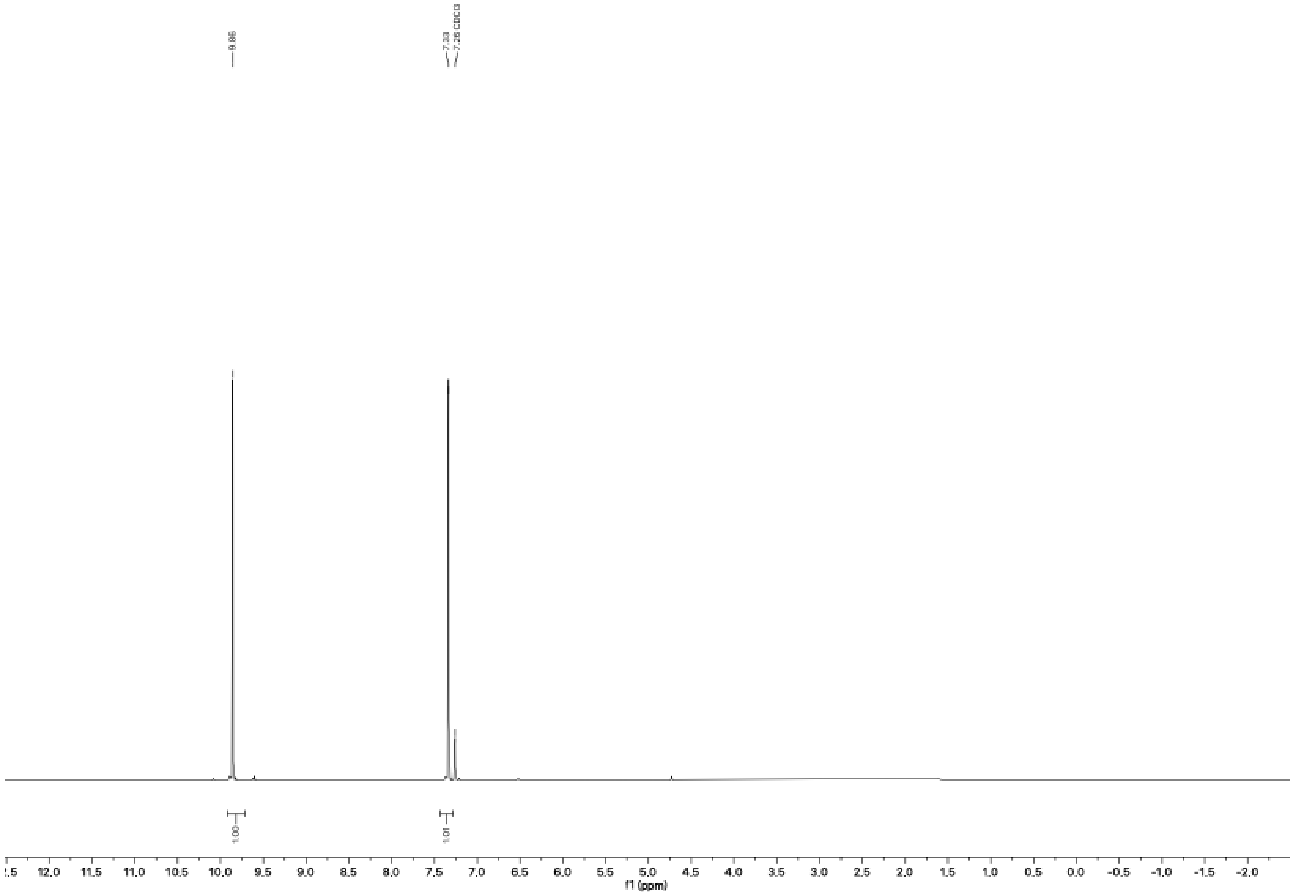

Supplementary Figure S9 ^{1}H NMR spectrum of DFF

^{13}C NMR (101 MHz, [$CDCl_3$]): δ (ppm) 179.33 (CHO-fur-CHO), 154.29 (CHO-CO=CH-CH=CO-CHO, furan), 119.34 (t, CHO-CO=CH-CH=CO-CHO, furan)

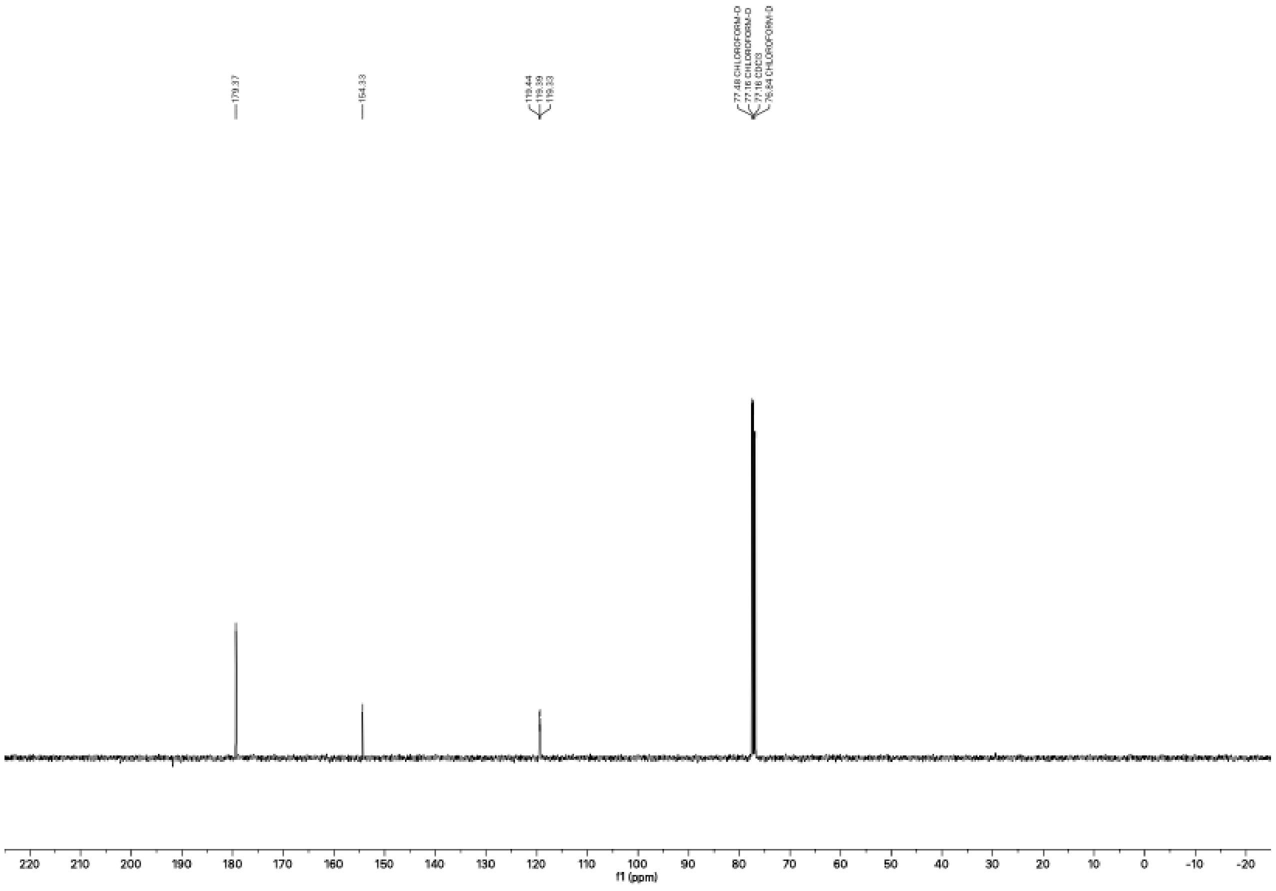

Supplementary Figure S10 ^{13}C NMR spectrum of DFF

*(2E,2’E)-3,3’-(Furan-2,5-diyl)diacrylic acid, **16**:*

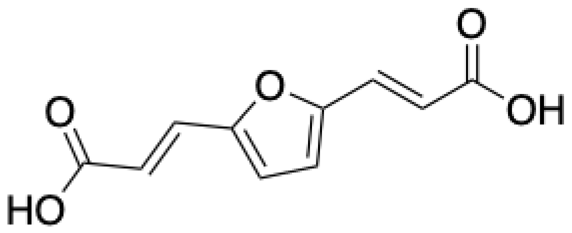

^{1}H NMR (400 MHz, [DMSO-d6]): δ (ppm) 7.34 (d, J = 15.8 Hz, =CO-CH=CH-COOH *2), 6.97 (s, -CO=CH-CH=CO-, furan), 6.34 (d, J = 15.8 Hz, =CO-CH=CH-COOH *2).

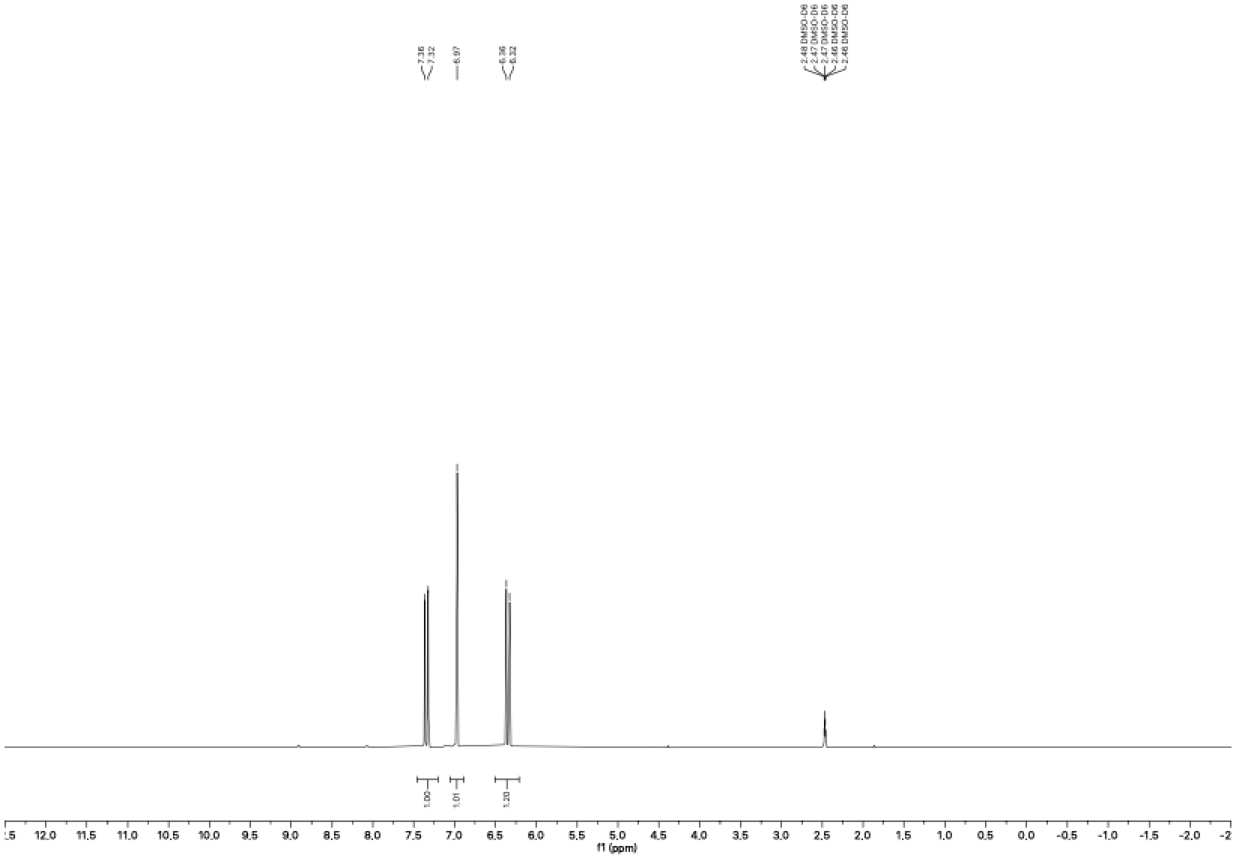

Supplementary Figure S11 ^{1}H NMR spectrum of **16**

^{13}C NMR (101 MHz; [DMSO-d6]): δ (ppm) 167.7 (=CO-CH=CH-COOH *2), 152.6 (=CO-CH=CH-COOH *2, furan), 130.5 (=CO-CH=CH-COOH *2), 118.9 (=CO-CH=CH-COOH *2), 118.1 (-CO=CH-CH=CO-, furan).

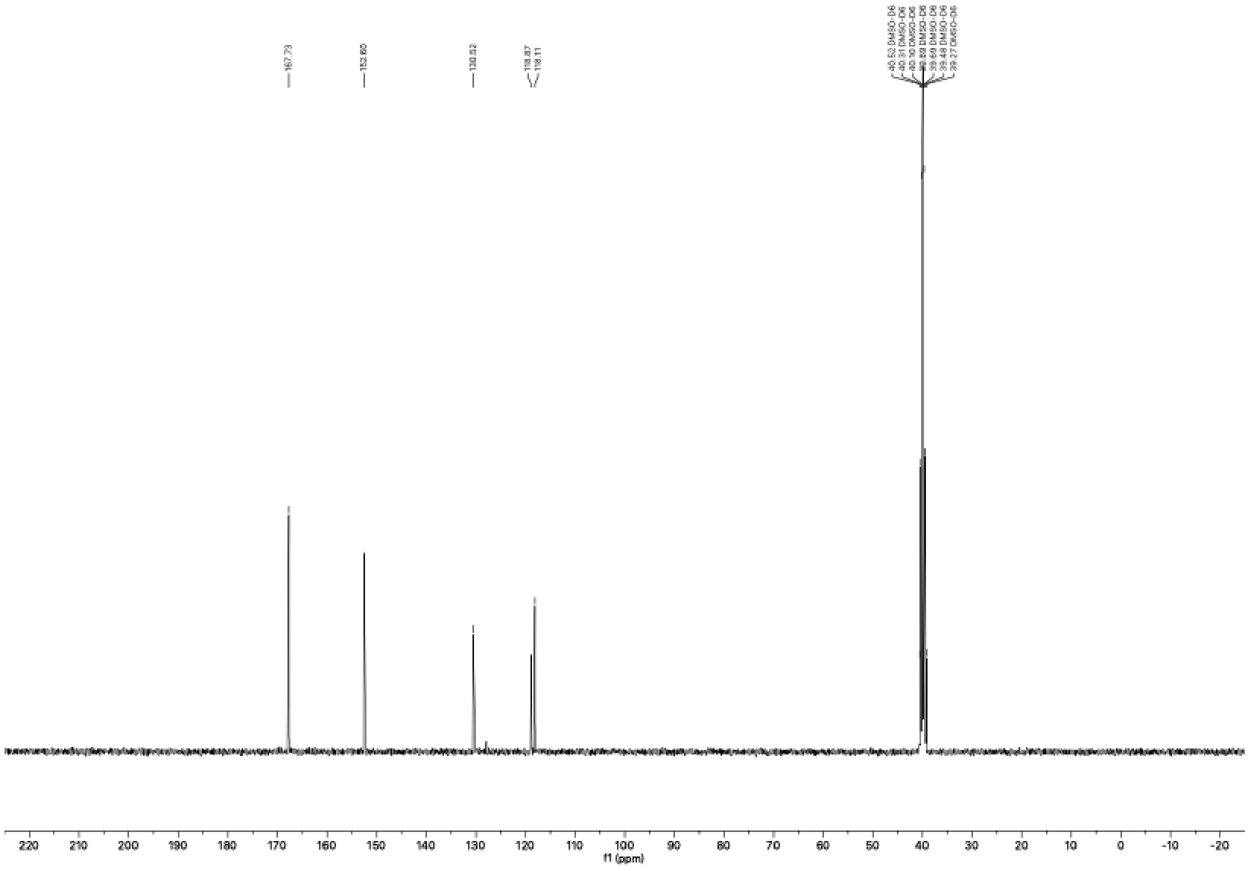

Supplementary Figure S12 ^{13}C NMR spectrum of **16**

ESI-MS: $C_{10}H_8NaO_5$, theoretical: 231.0264 [M^+], measured: 231.0271 [M^+].

Melting point: Decomposes >200 °C.

*Dimethyl (2E,2'E)-3,3'-(Furan-2,5-diyl)diacrylic ester, monomer **1**:*

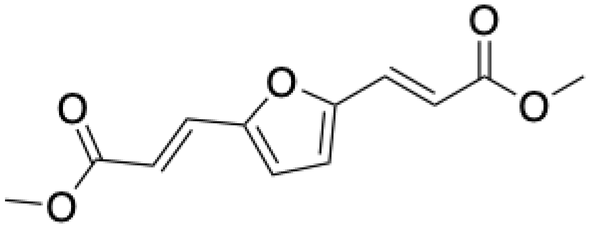

^{1}H-NMR (400 MHz, [$CDCl_3$]): δ (ppm) 7.40 (2H, d, J = 15.7 Hz, =CO-CH=CH-COOMe *2), 6.65 (2H, s, -CO=CH-CH=CO-, furan), 6.43 (2H, d, J = 15.8 Hz, =CO-CH=CH-COOMe *2), 3.80 (s, CH_3-OOC-CH=CH-).

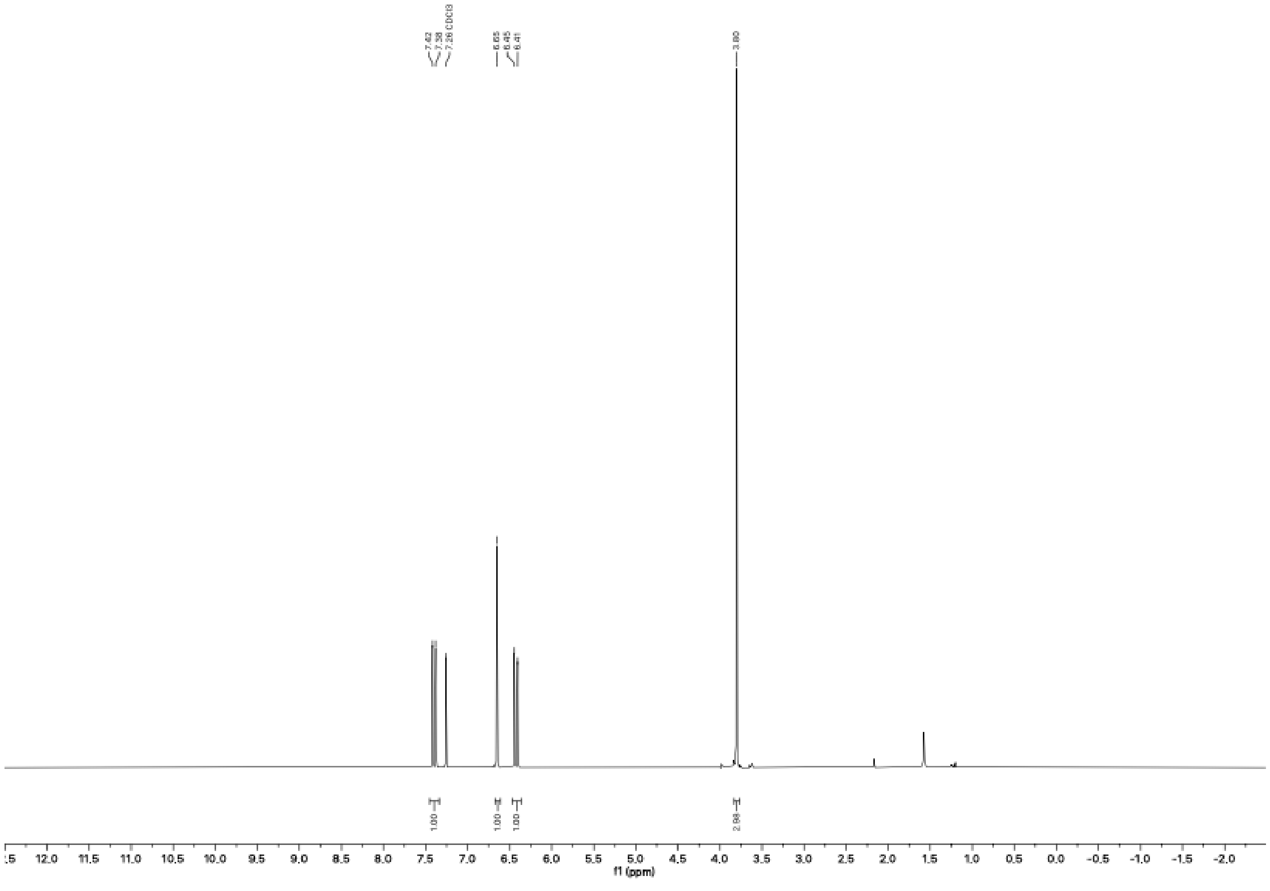

Supplementary Figure S13 ^{1}H NMR spectrum of monomer **1**

^{13}C NMR (101 MHz; [$CDCl_3$]): δ (ppm) 167.2 (=CO-CH=CH-COOMe *2), 152.5 (=CO-CH=CH-COOMe *2, furan), 130.4 (=CO-CH=CH-COOMe *2), 117.6 (=CO-CH=CH-COOMe *2), 116.9 (-CO=CH-CH=CO-, furan), 52.0 (=CO-CH=CH-COO-CH_3*2).

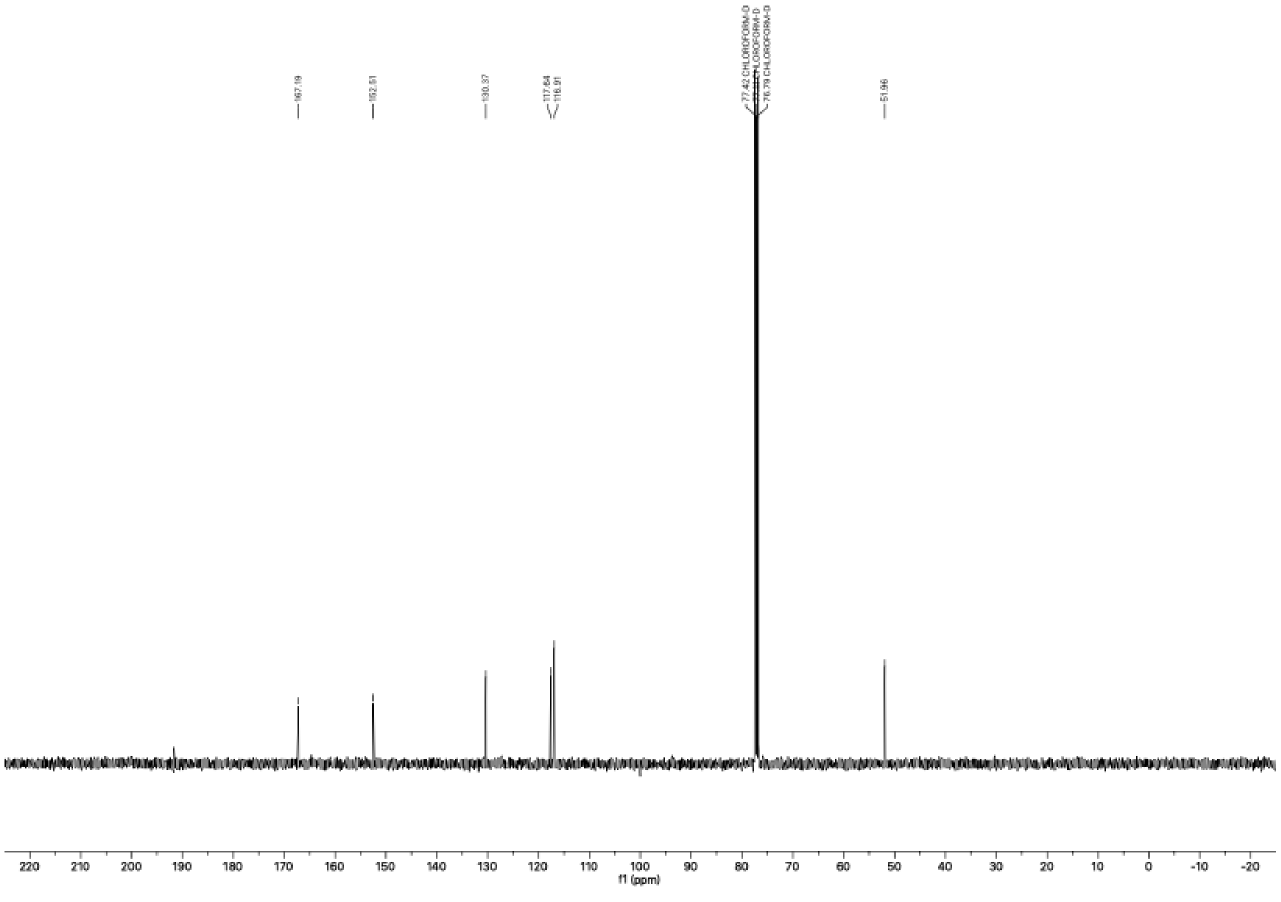

Supplementary Figure S14 ^{13}C NMR spectrum of monomer **1**

ESI-MS: $C_{12}H_{12}NaO_5$, theoretical (m/z): 259.0577 [M^+] measured (m/z): 259.0759 [M^+].

Melting point: 153.7 – 155.9 °C. (lit. 152-153 °C)[4]

Poly-2,5-dioctylacrylic ester furan (ODO-PFAE):

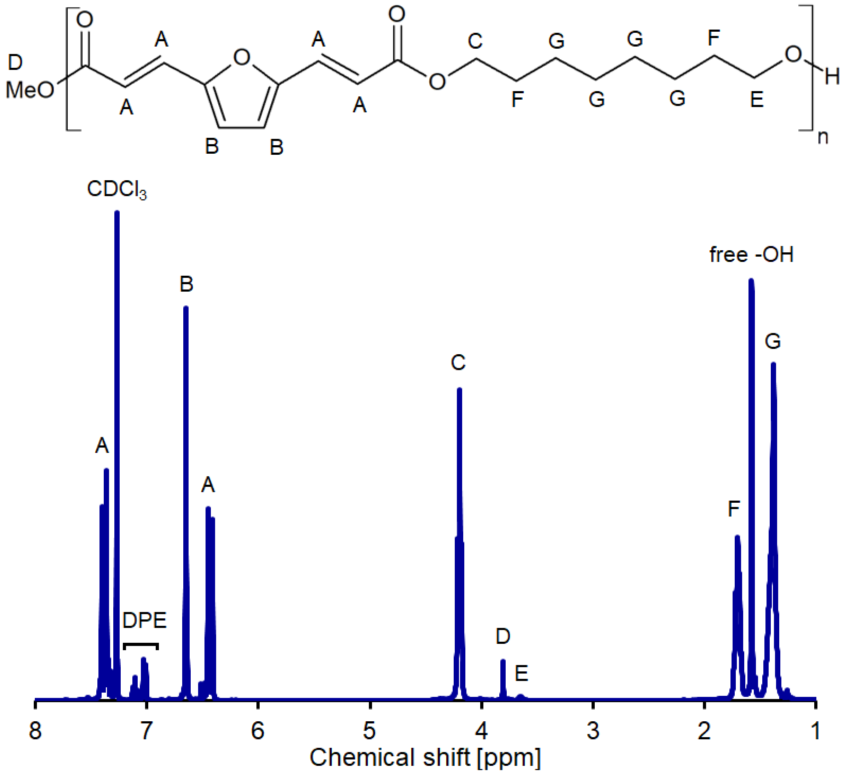

Supplementary Figure S15 1H-NMR analysis of the ODO-PFAE synthesised in diphenyl ether after the three purification steps.

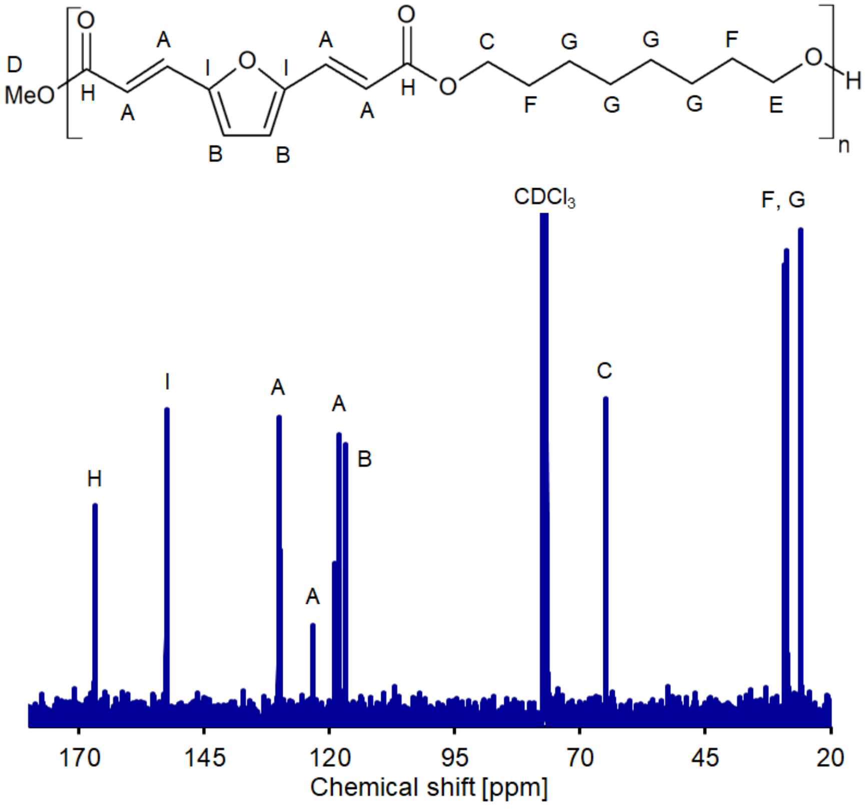

Supplementary Figure S16 ^{13}C-NMR analysis of the ODO-PFAE synthesised in diphenyl ether after the three purification steps.

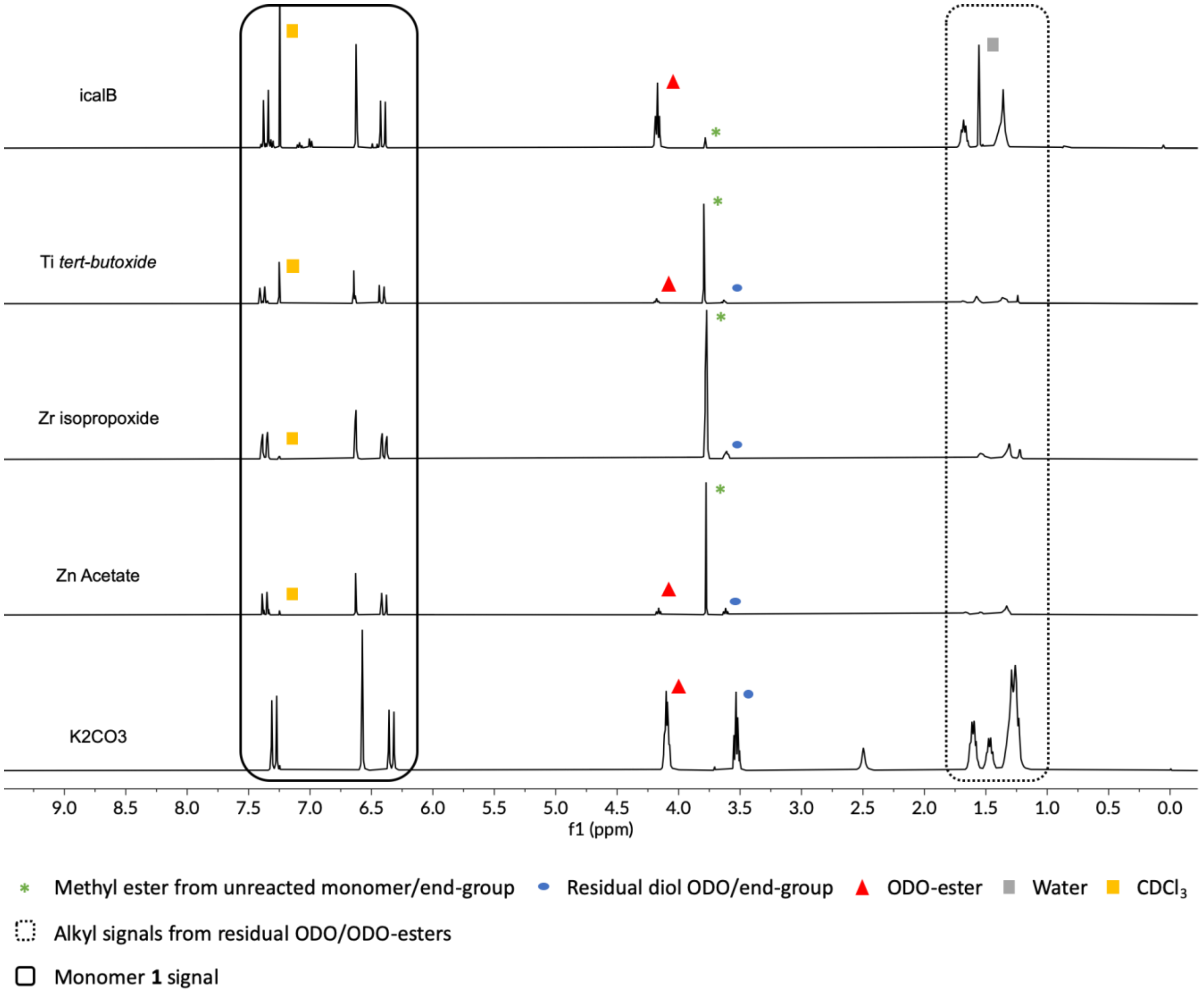

Supplementary Figure S17 Overlay of ^{1}H-NMR spectra for $CHCl_3$-soluble crude products obtained from chemo-catalysed polycondensations with 1,8-octanediol (ODO) and purified polymers obtained after enzymatically-catalysed polymerisation

The absence of methyl ester signals (3.75 ppm) from K_2CO_3-catalysed reaction shows that good conversion was obtained with this catalyst, but only short oligomers could be formed (see GPC results table S4). This milder catalyst likely avoided branching side reactions which could explain the low M_n. The higher M_n obtained with $Ti(O^tBu)_4$ might be explained by branching that readily occurred with this catalyst (and Zr isopropoxide and Zn acetate) which would result in an increase of M_n in spite of the low conversion observed by ^{1}H-NMR.

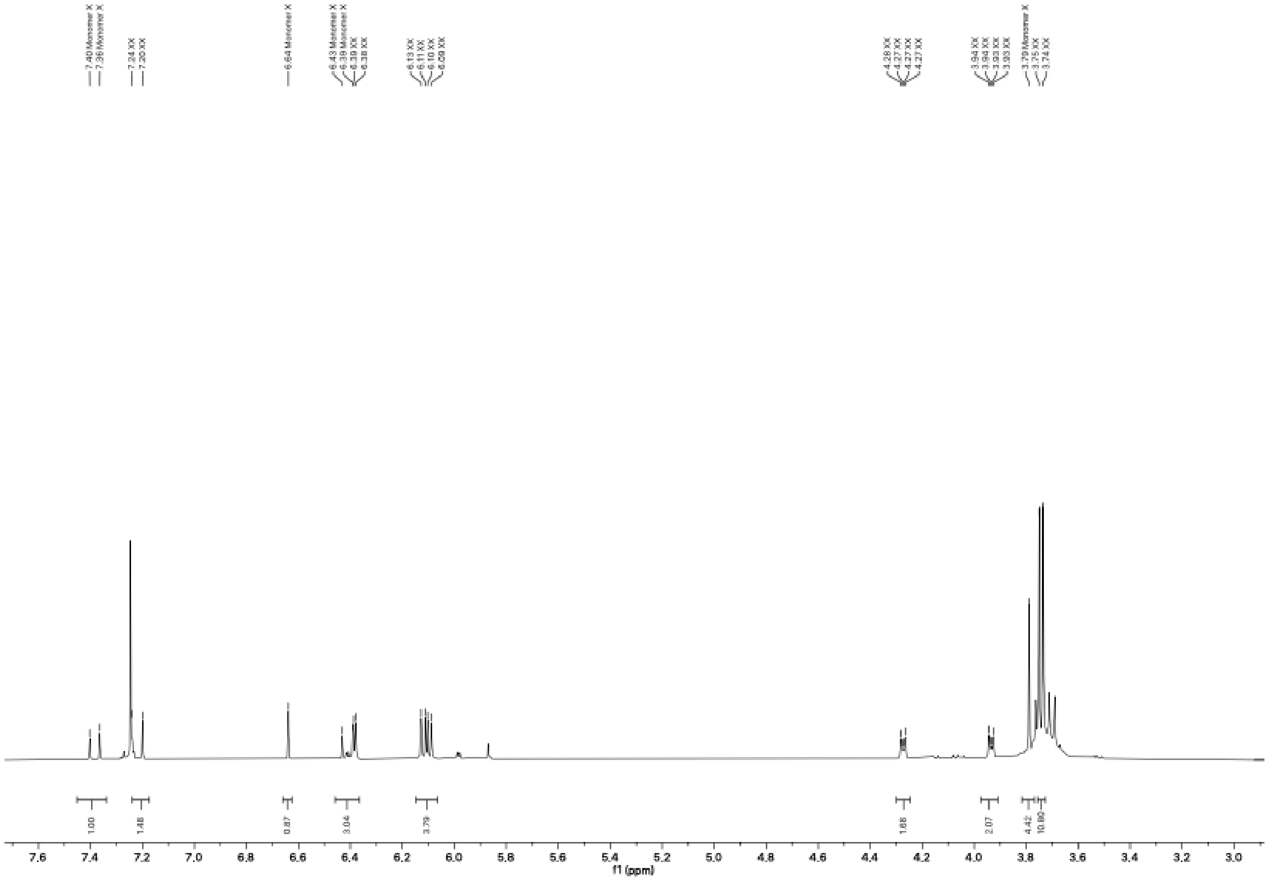

Supplementary Figure S18 ^{1}H NMR spectrum of crude product obtained after 24 h UV-irradiation of monomer **1** (signals indicate monomer **1** and cycloadduct **2**)

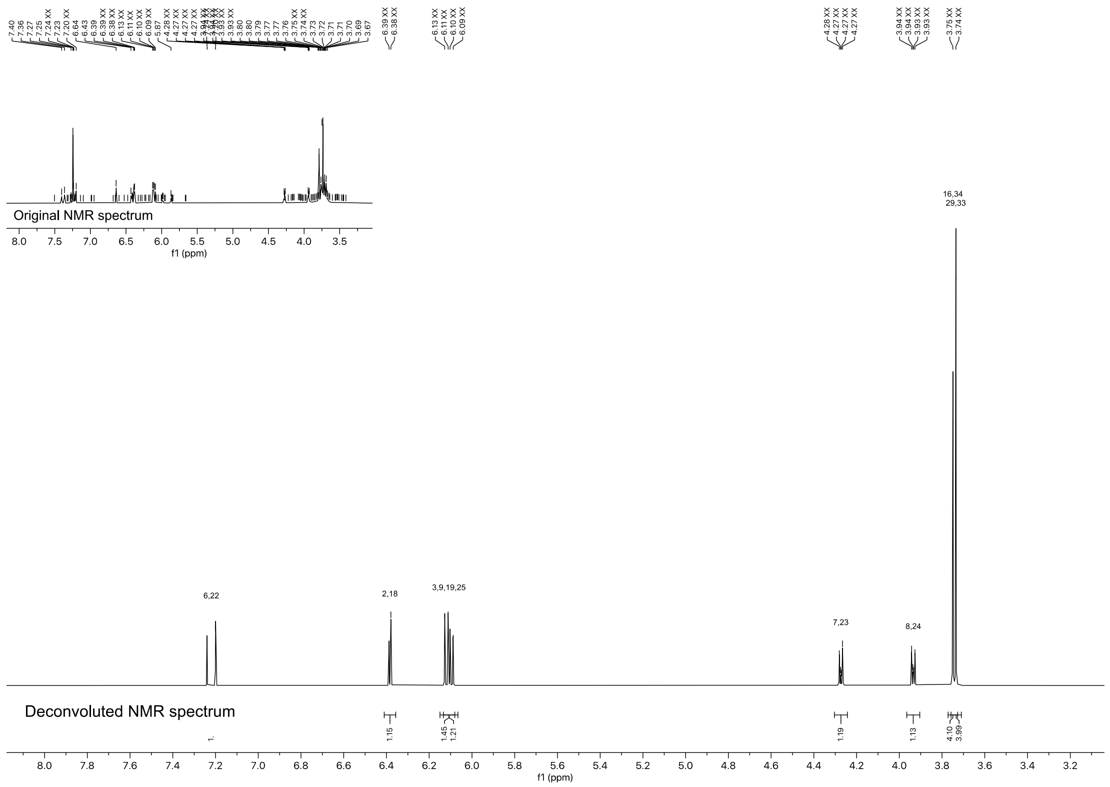

Supplementary Figure S19 Deconvoluted ^{1}H NMR spectrum for the isolation of cycloadduct **2** signals after 24 h of irradiation of **1**

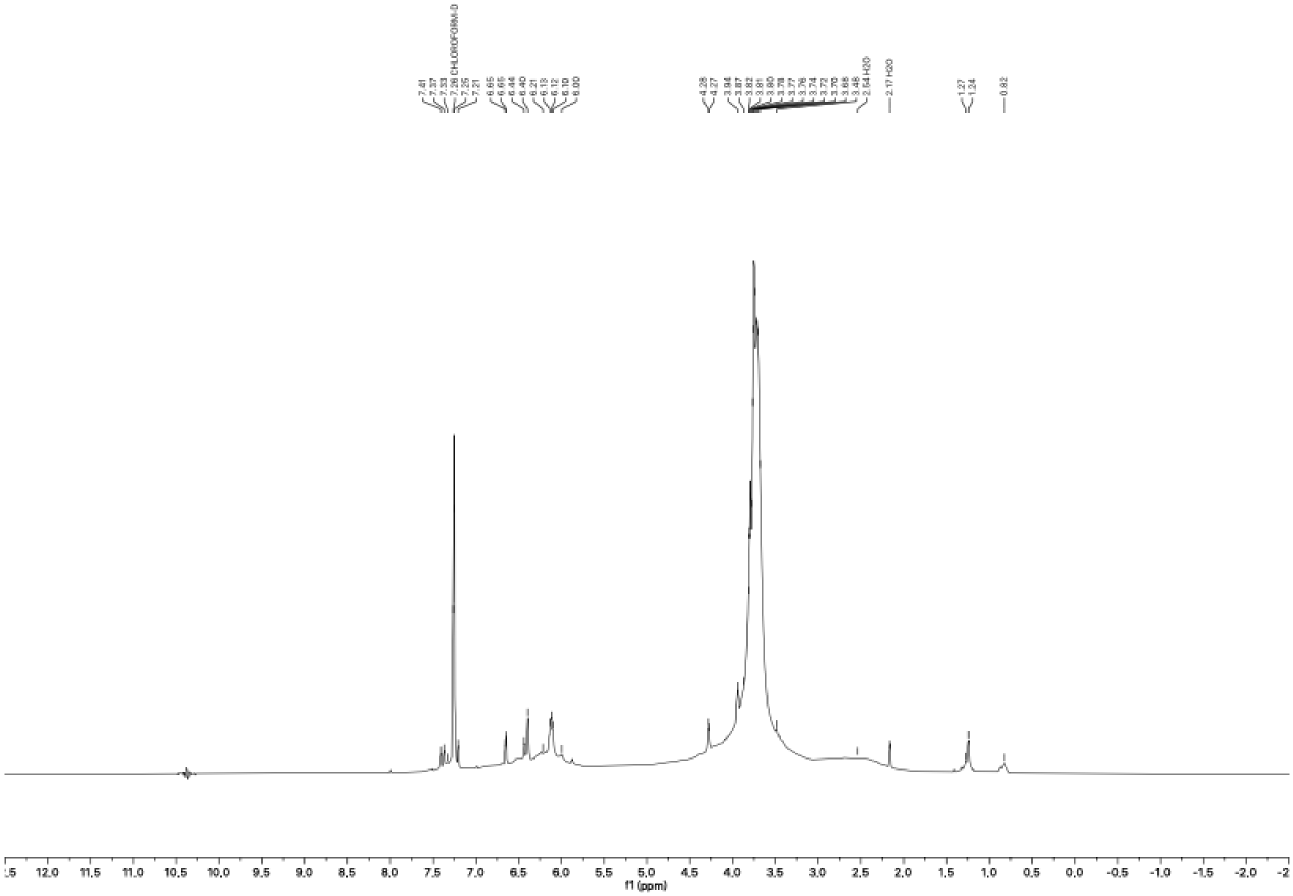

Supplementary Figure S20 ^{1}H NMR spectrum of crude product obtained after 72 h UV-irradiation of monomer **1**

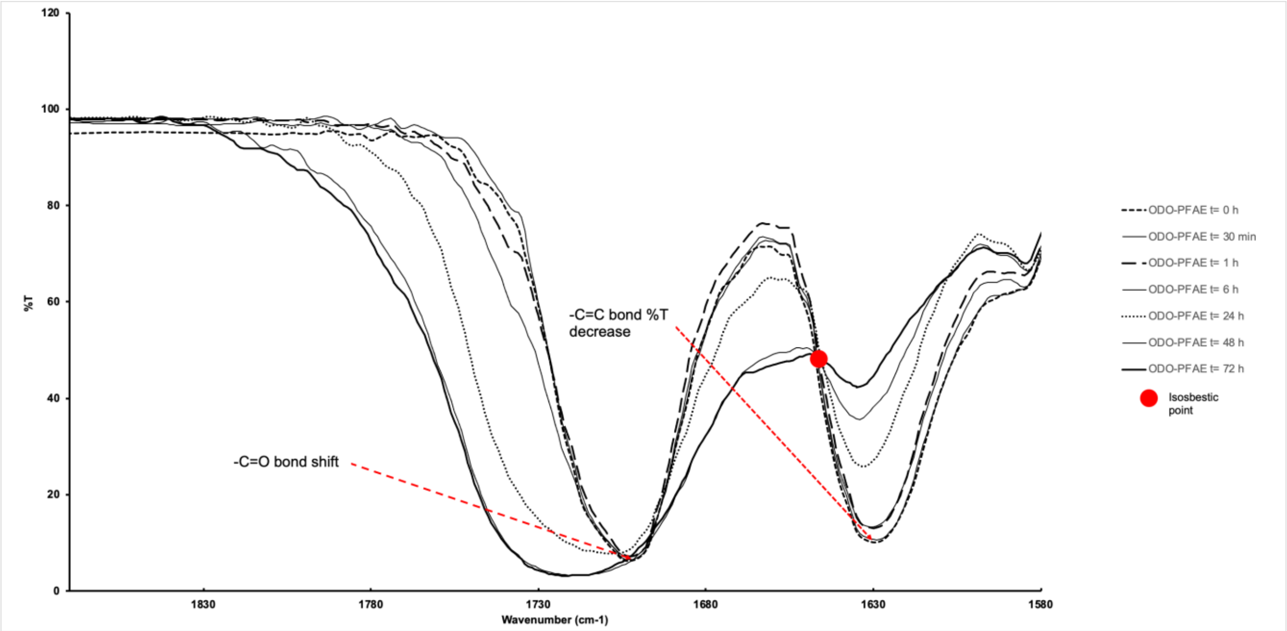

Supplementary Figure S21 Overlay FT-IR spectra of UV-cured polymer ODO-PFAE after different irradiation time

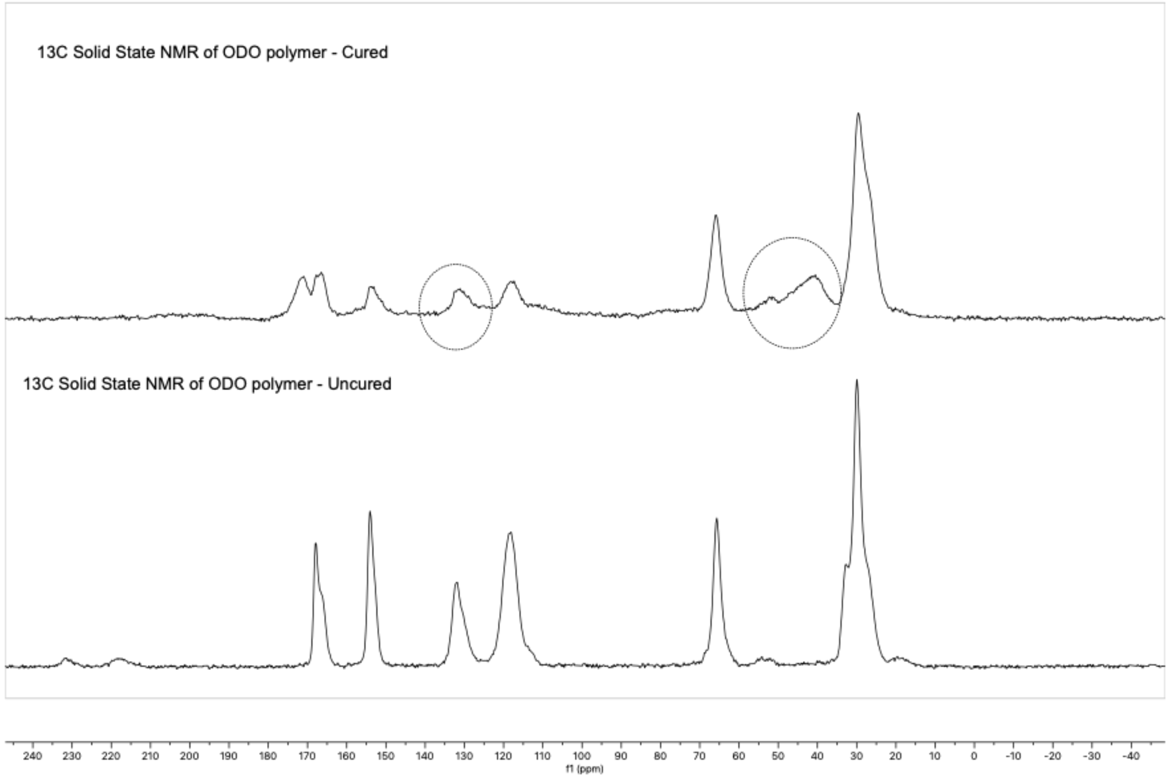

Supplementary Figure S22 ^{13}C solid state NMR spectra of the cured (top) and uncured (bottom) ODO polymer. Dotted circles show the reduction of the alkene signal and appearance of the cyclobutane signal between 40-50 ppm

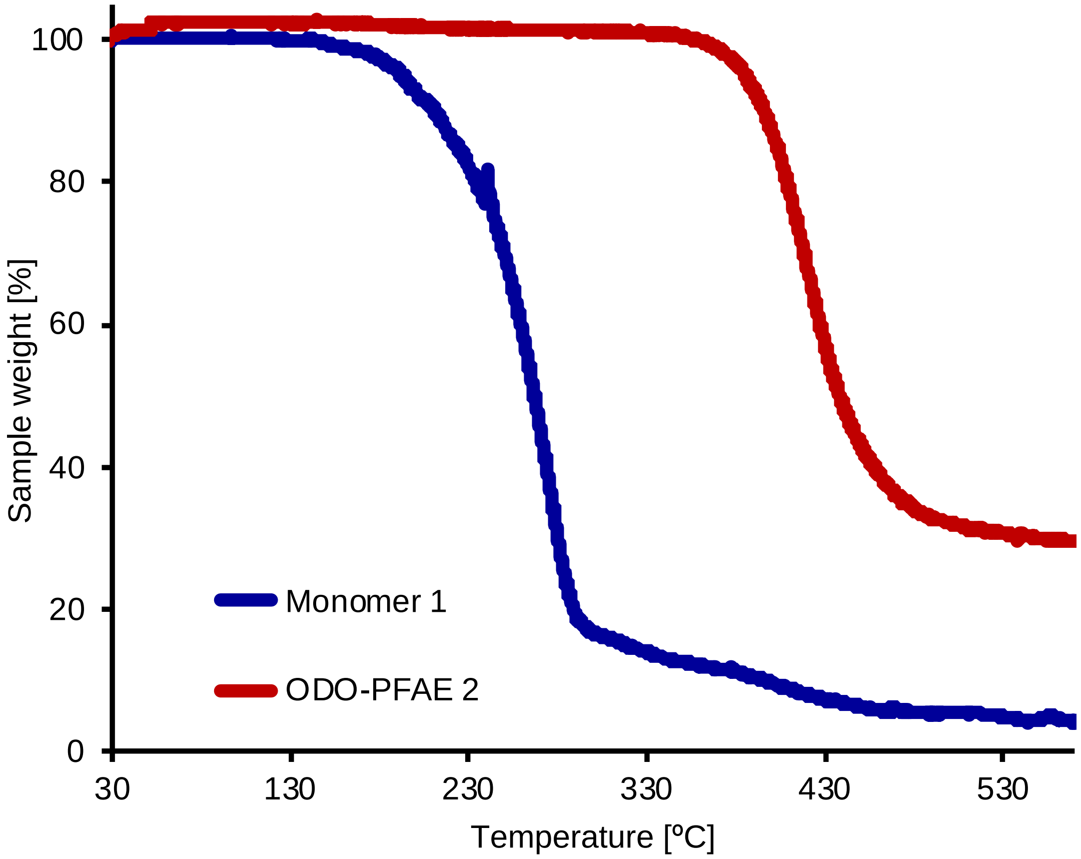

Supplementary Figure S23 TGA analysis of monomer **1** and of the polymer synthesised from **1** and 1,8-octanediol (ODO) in DPE using immobilized CaLB as the biocatalyst

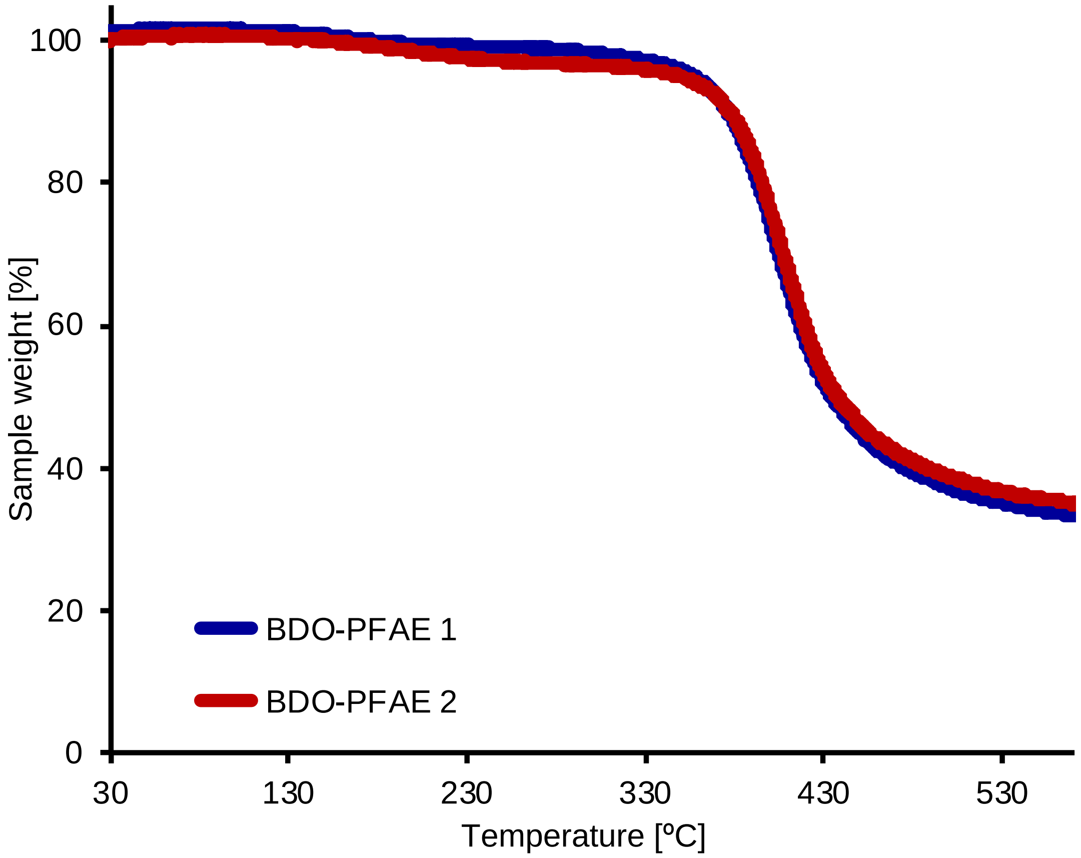

Supplementary Figure S24 TGA analysis of the polymers synthesised (in duplicate) from **1** and 1,4-butanediol (BDO) in DPE using immobilized CaLB as the biocatalyst

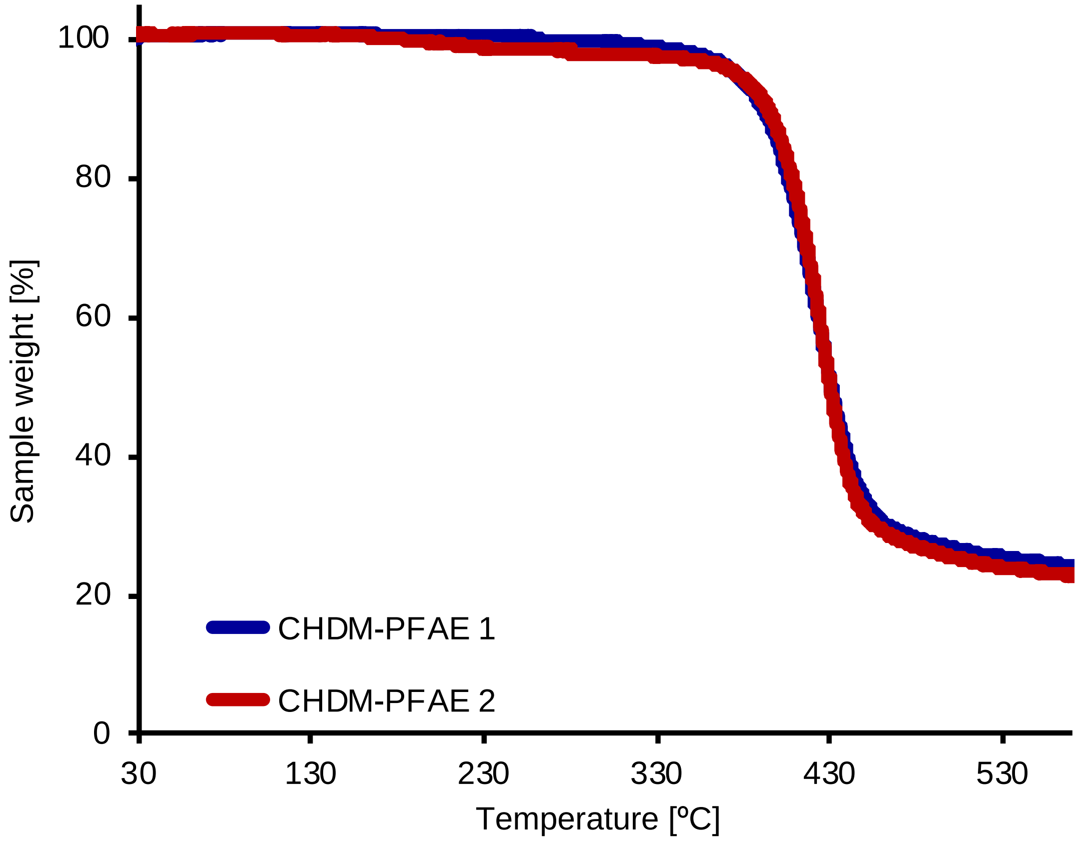

Supplementary Figure S25 TGA analysis of the polymers synthesised (in duplicate) from **1** and 1,4-cyclohexanedimethanol in DPE using immobilized CaLB as the biocatalyst

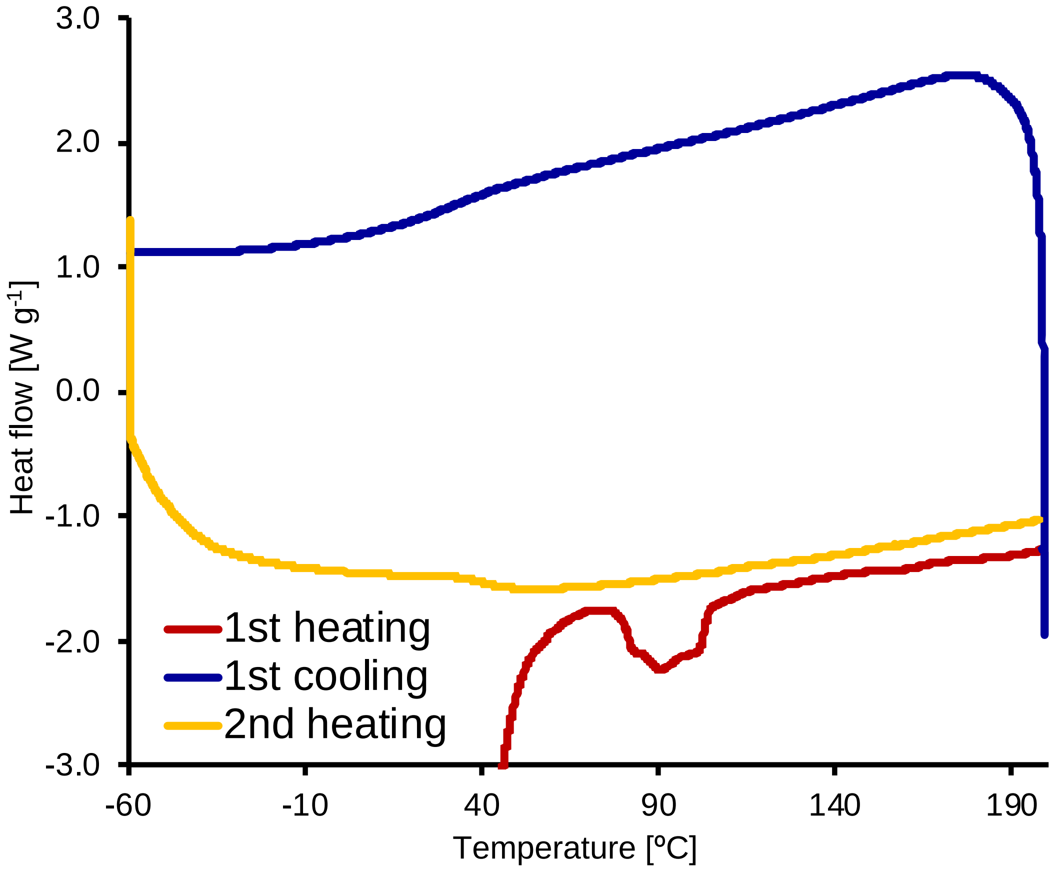

Supplementary Figure S26 DSC thermogram (**red**, 1st heating, **blue** 1st cooling and **orange** 2nd heating cycle) of the PFAE containing BDO with DPE as the solvent

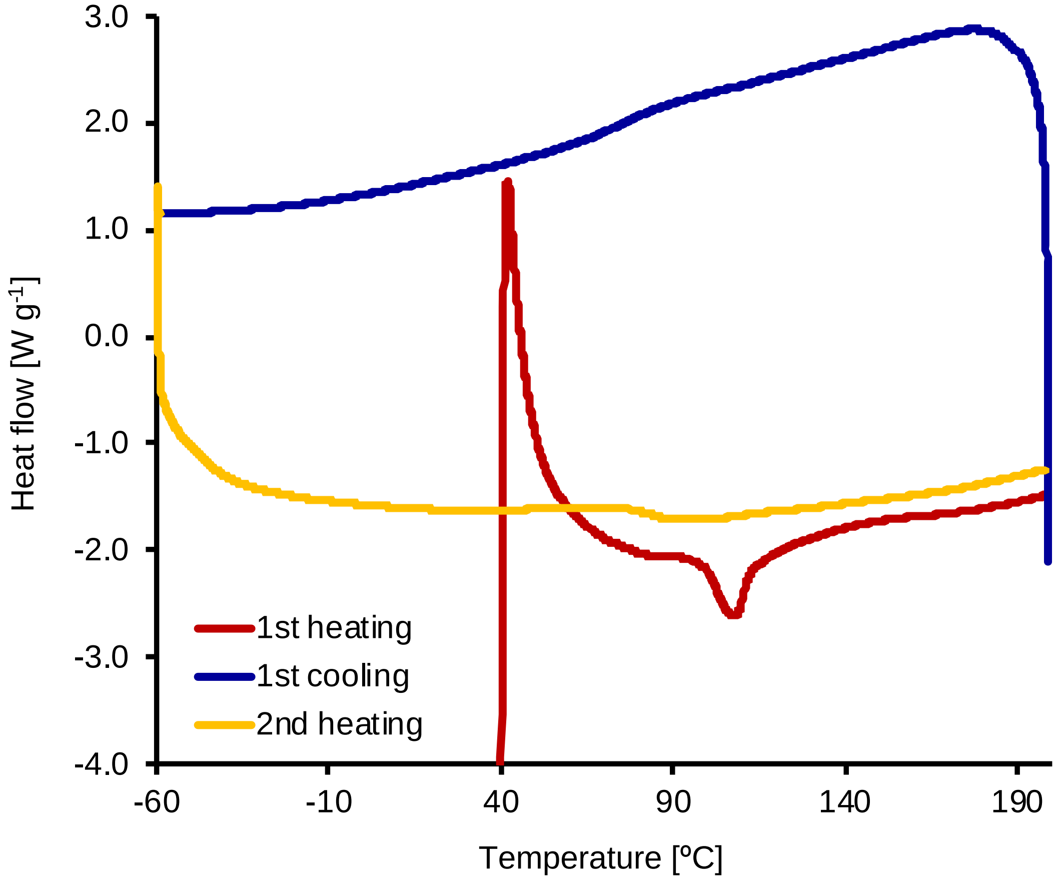

Supplementary Figure S27 DSC thermogram (**red**, 1st heating, **blue** 1st cooling and **orange** 2nd heating cycle) of the PFAE containing ODO as the diol with DPE as the solvent

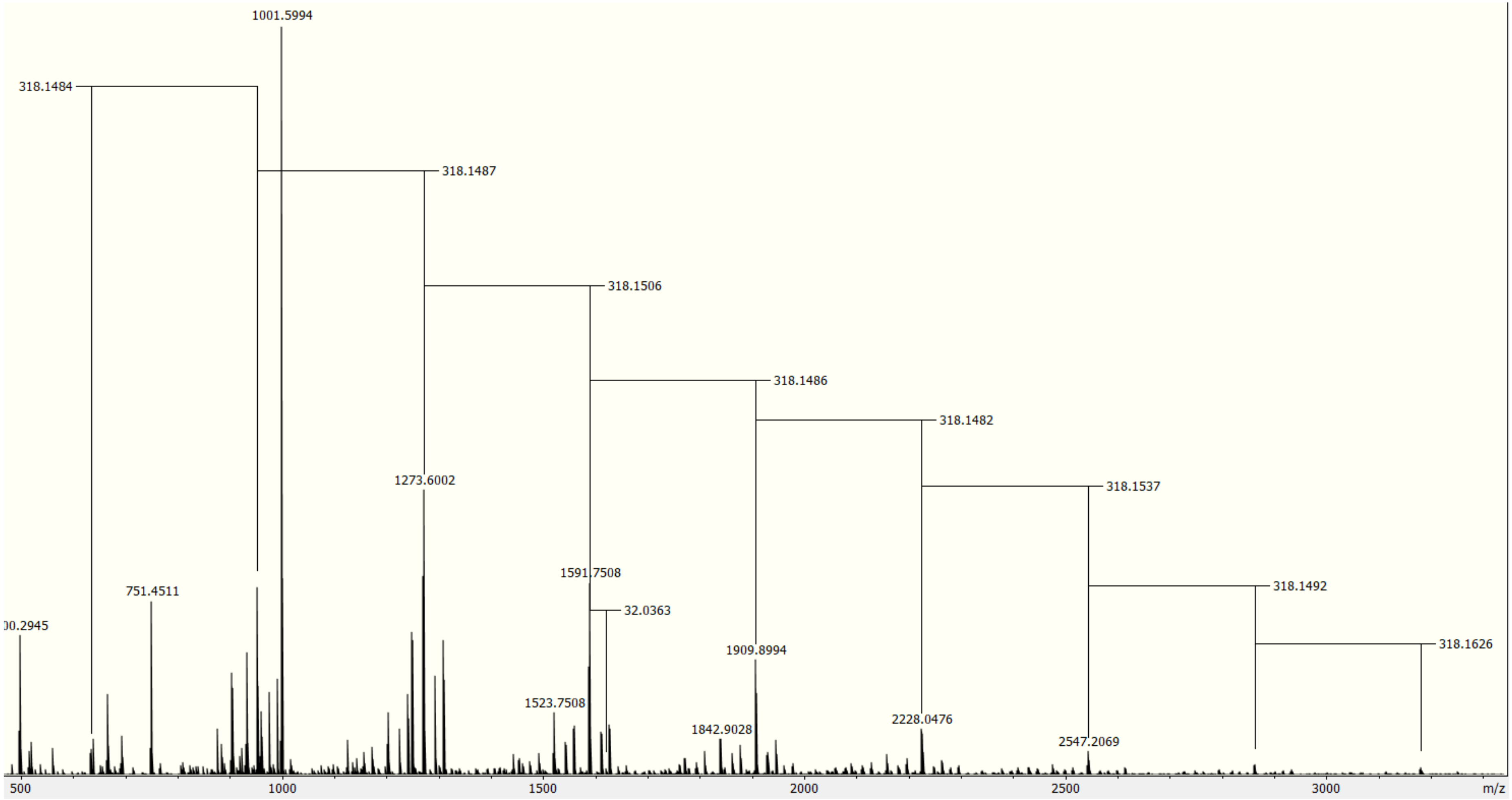

Supplementary Figure S28 MALDI analysis of ODO-PFAE and assignment of the main repetitive unit

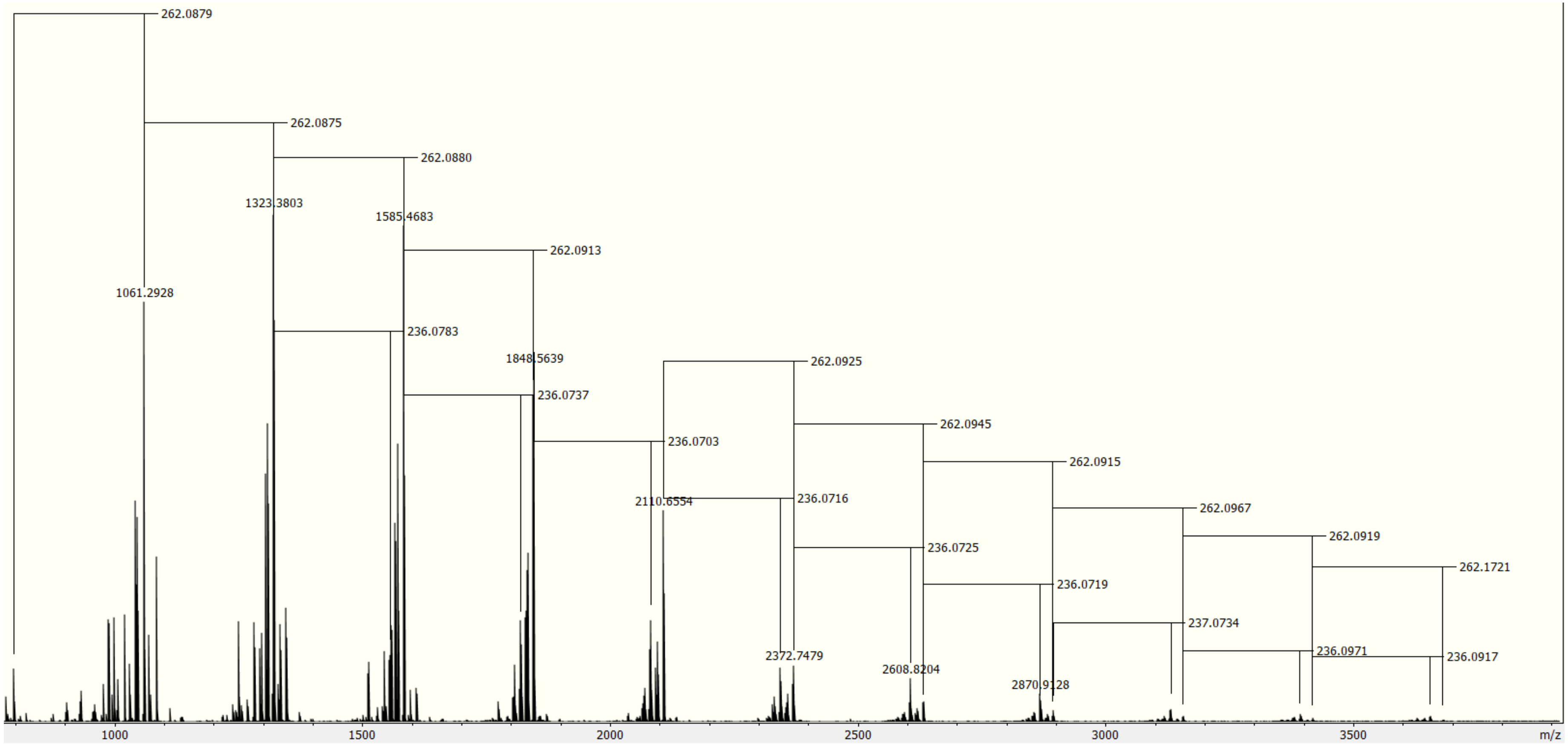

Supplementary Figure S29 MALDI analysis of BDO-PFAE and assignment of the main repetitive unit

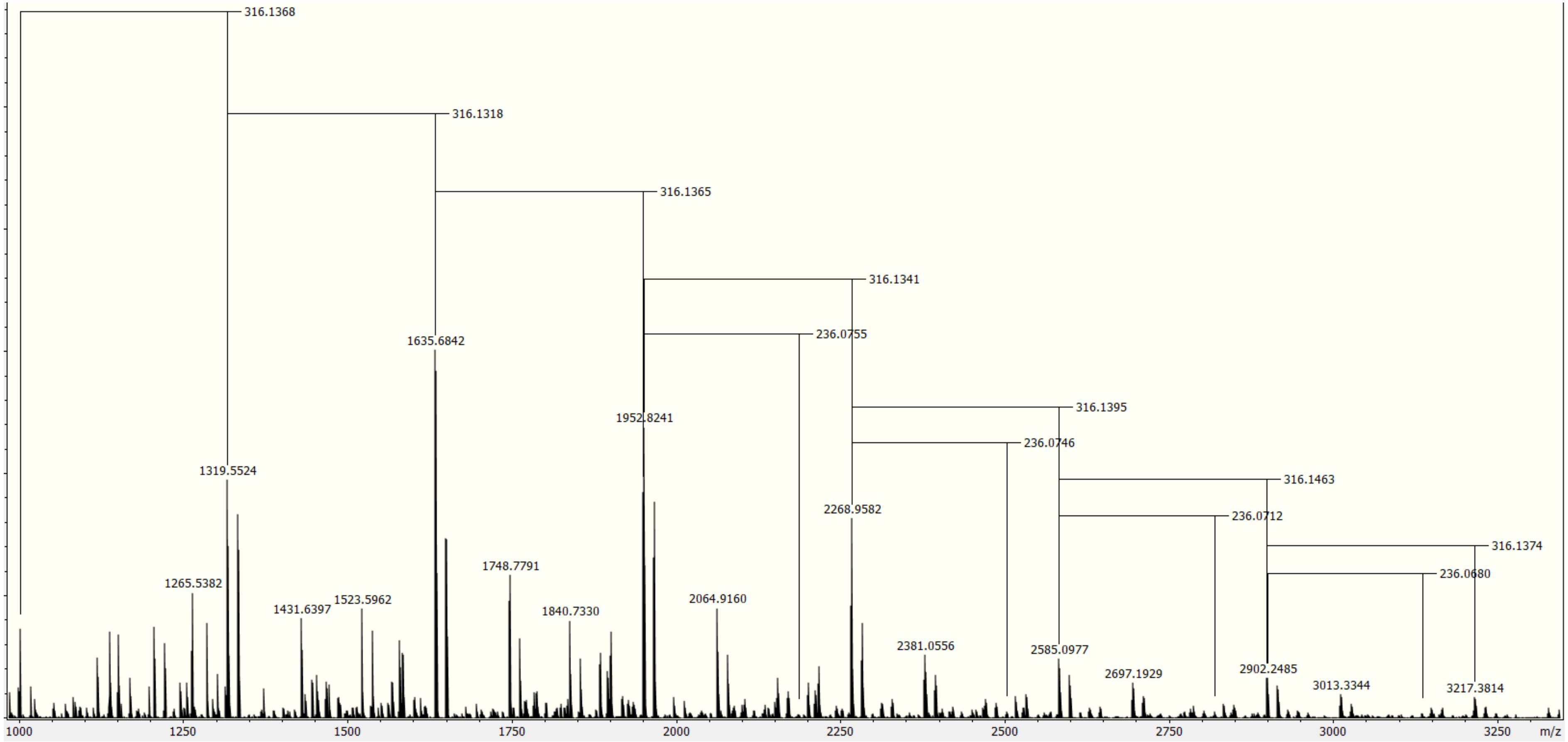

Supplementary Figure S30 MALDI analysis of CHDM-PFAE and assignment of the main repetitive unit

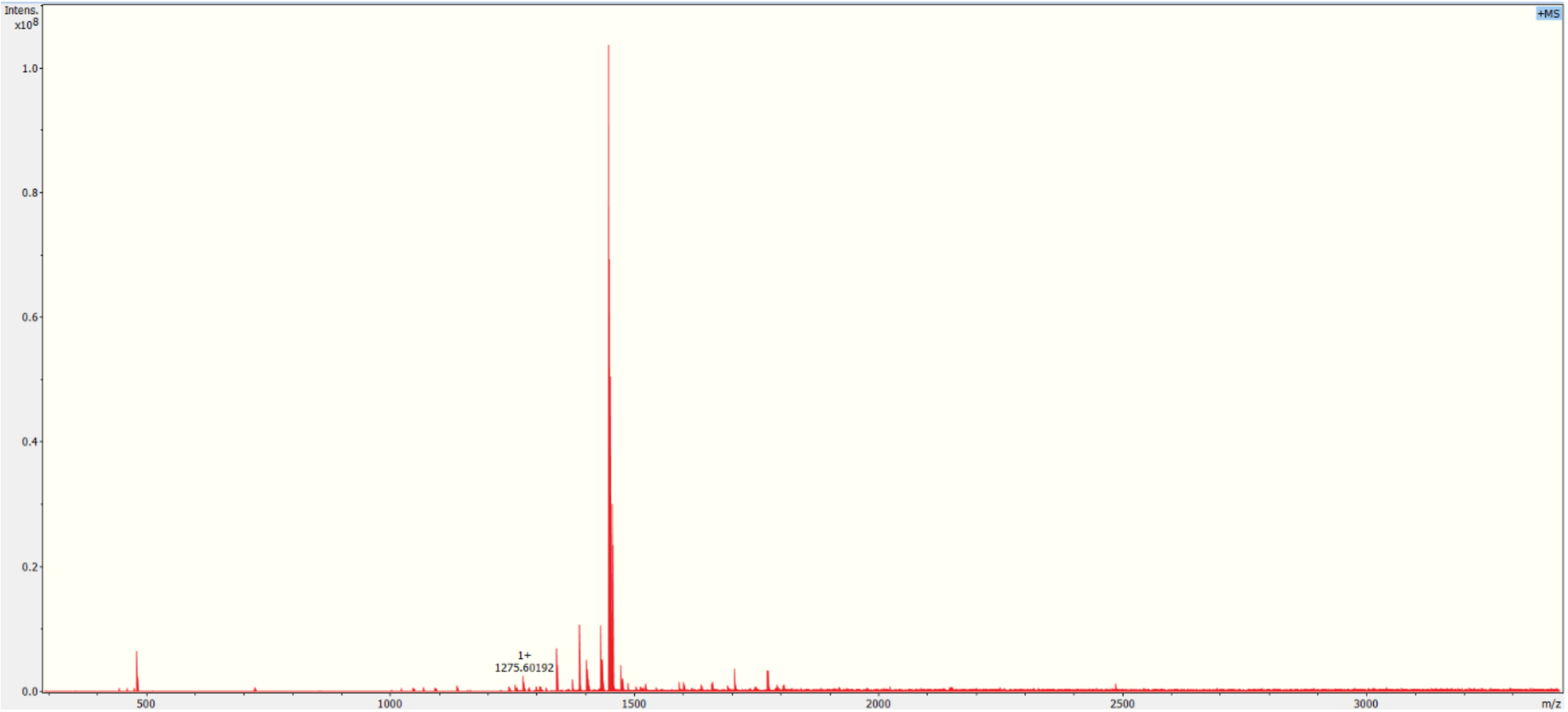

Supplementary Figure S31 MALDI analysis of ODO-PFAE synthesised using zinc as the catalyst

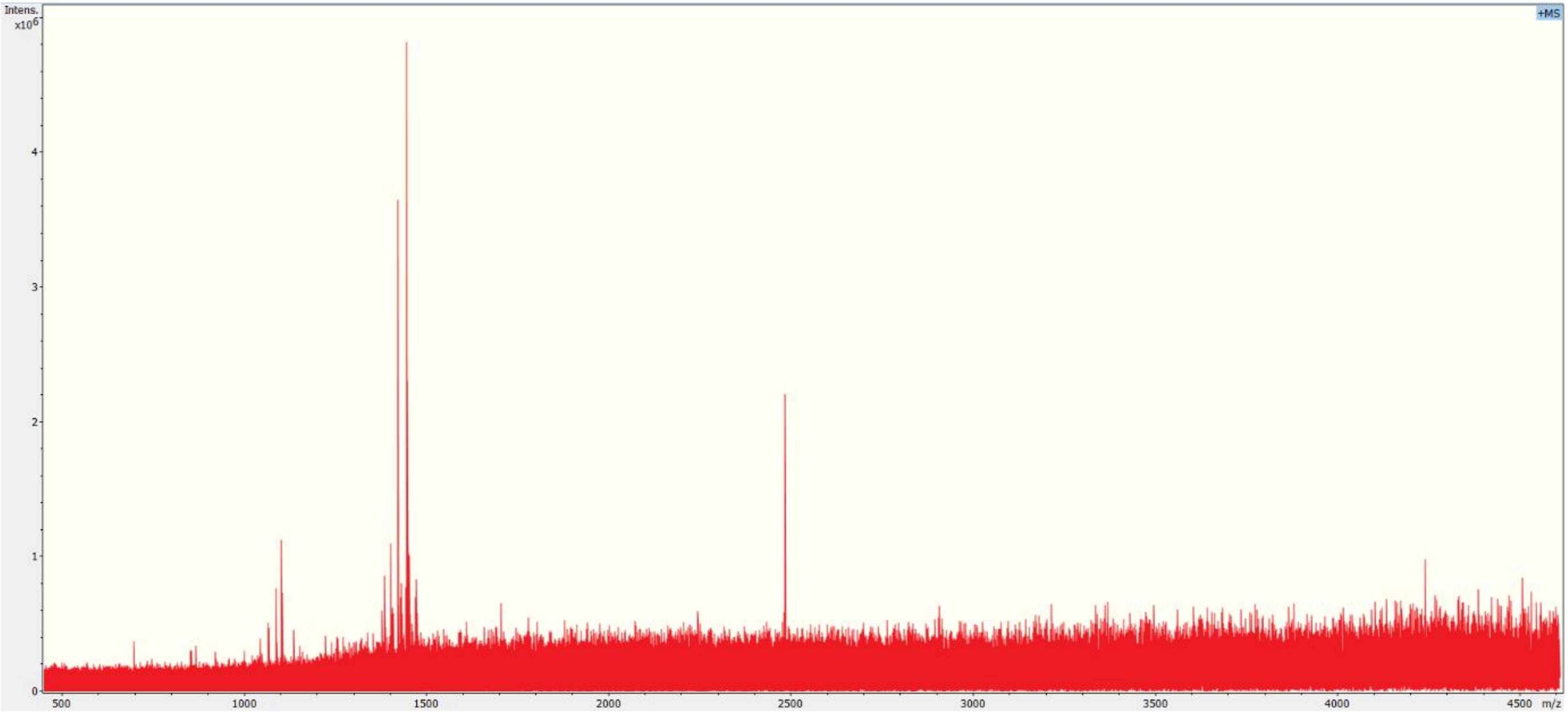

Supplementary Figure S32 MALDI analysis of ODO-PFAE synthesised using zirconium as the catalyst

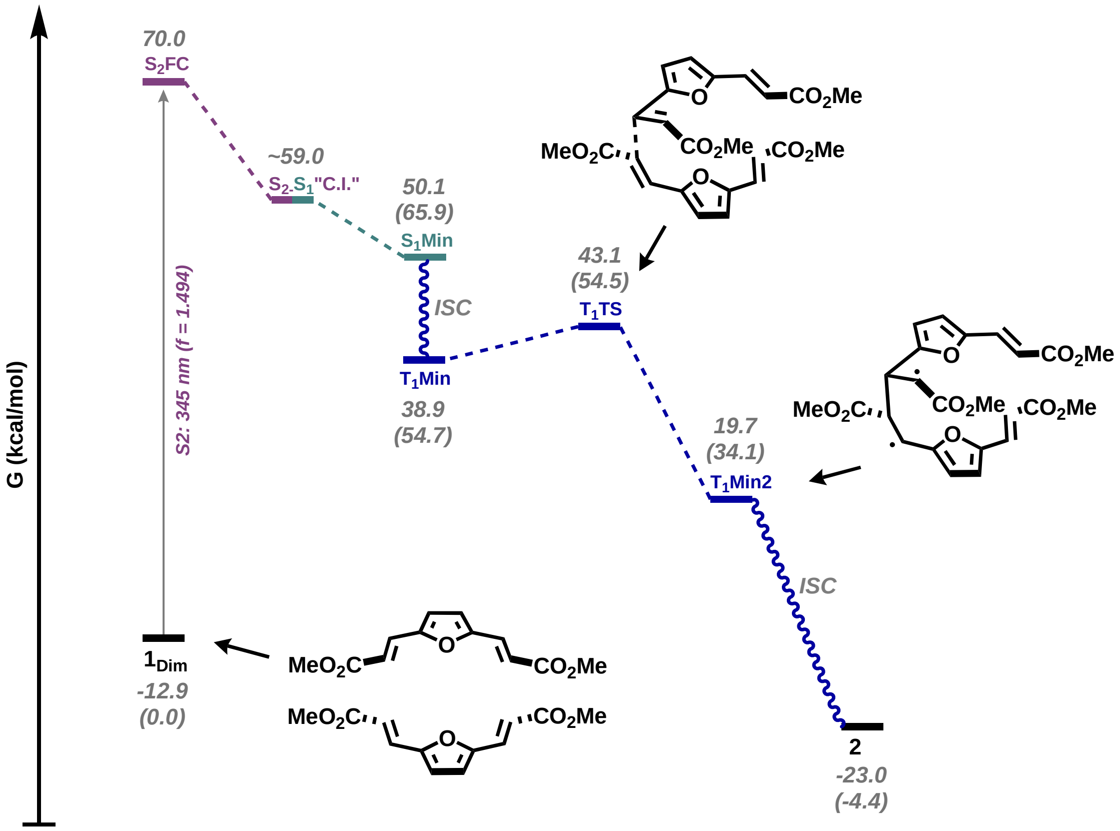

Supplementary Figure S33 Energy profile for cycloadduct formation through the triplet energy surface. Potential energies and free energies (in parentheses) in kcal mol^{-1}

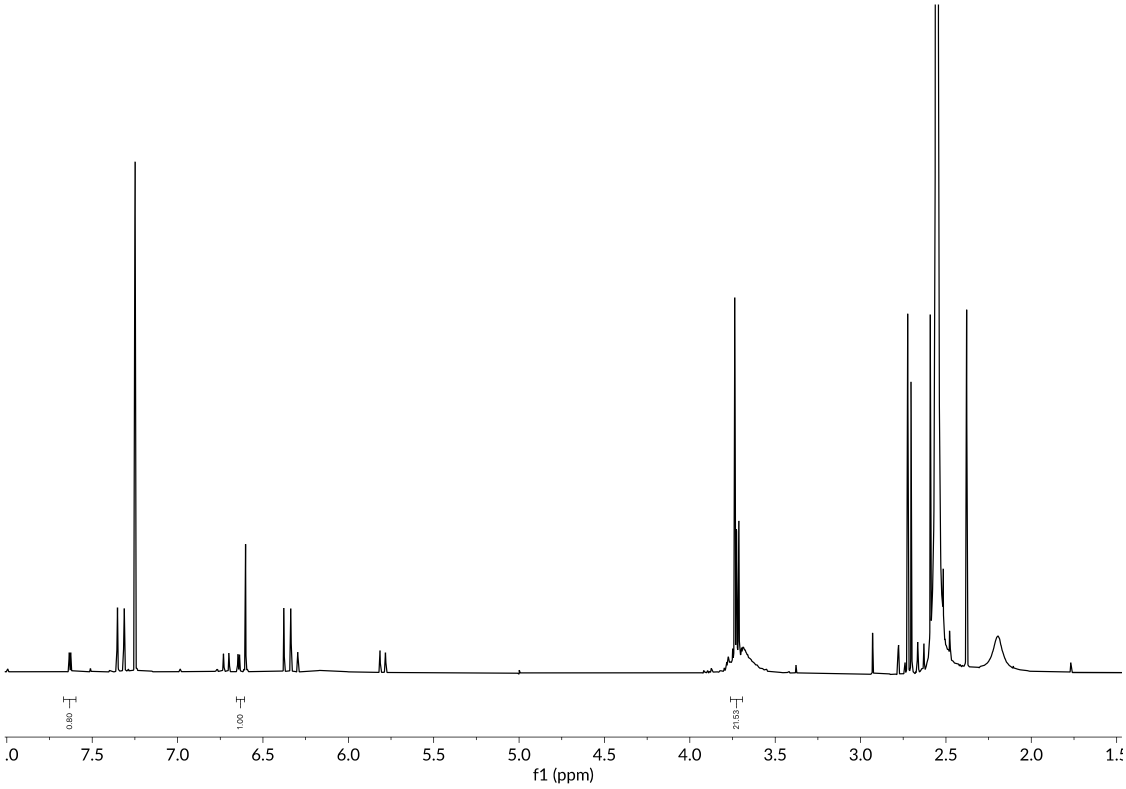

Supplementary Figure S34 ^{1}H NMR of dilute irradiated solution of **1** after 50 h, integrated signal show the presence of *E*-*Z* isomer (0.01 M in $CDCl_3$ irradiation done in 1 cm quartz cuvette, signals between 2.0 and 3.0 ppm are due to the DMSO used as internal standard)

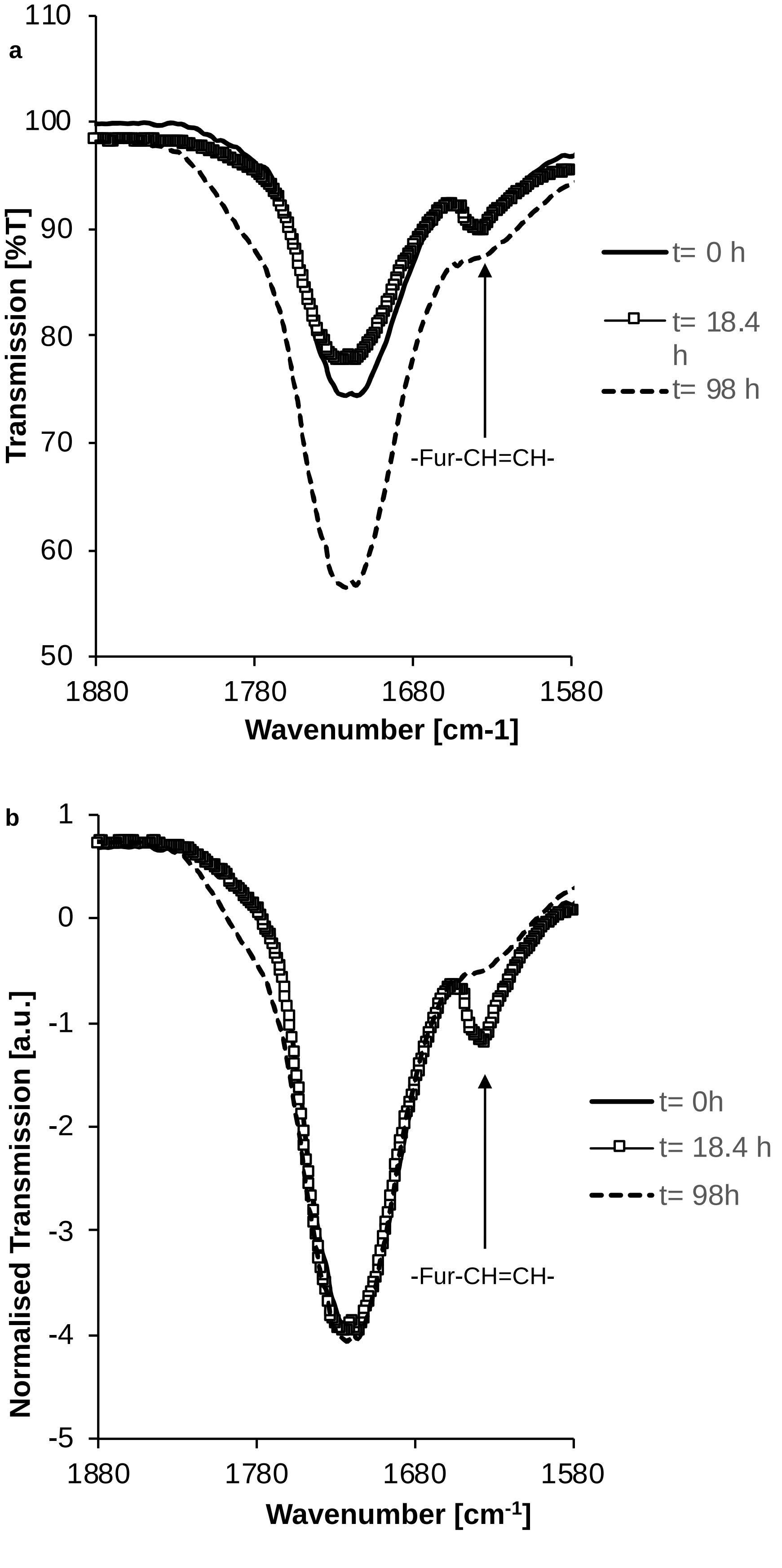

Supplementary Figure S35 Overlay of FT-IR spectrum of photoinduced reversed cycloaddition trials (monomer **1** cured for >72 h was used and irradiated at indicated times) **a**: Non-standardised data **b**: Standardised data using excel built-in function

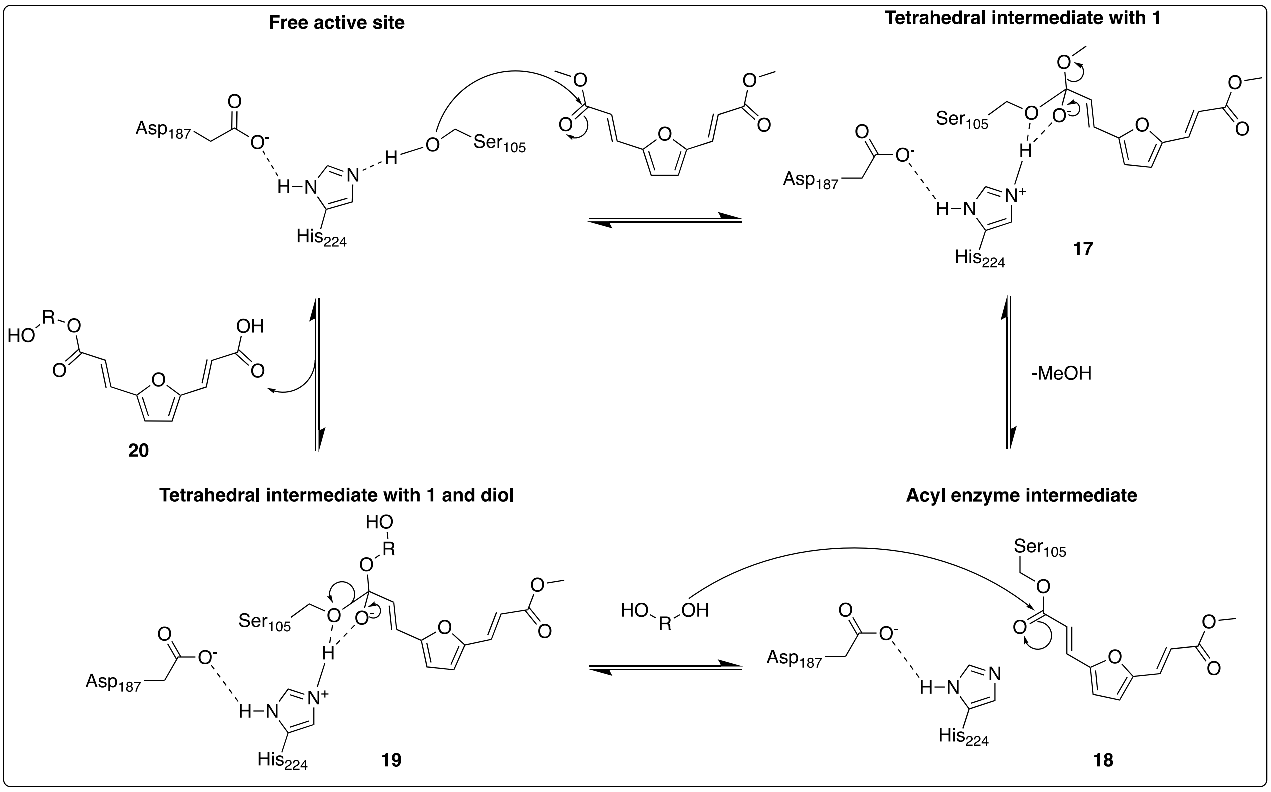

Supplementary Figure S36 Proposed mechanism for the transesterification catalysed by the serine hydrolase enzyme CaLB

The enzyme employed belongs to the serine hydrolase superfamily the mechanism of which has previously been investigated.[9] The conjugation of the monomer **1** is temporarily broken during the formation of a tetrahedral intermediate (Figure S36, **17**) with a serine residue present in CaLB's active site. The transesterification with the diols then occurs with attack of the diol on the acyl enzyme intermediate (Figure S36, **18**) which formed from **17** after release of methanol. The elongated product (**20**) is released from the second tetrahedral species formed (Figure S36, **19**), which closes the catalytic cycle by reforming the active site. As such the relatively low M_n obtained here is possibly due to the need for the highly stable extended conjugation of **1** to be temporarily partially broken for formation of intermediate **17**.[9] The low M_n may also be due to the steric hindrance created by the elongated chains of the polymer. The longer bulky oligomers likely prevent the substrate from entering the CaLB's active site and thus limit the further growth of the polymer. It is in fact well known that fully aliphatic polyesters can reach far higher molecular weights compared to terephthalate, furan and pyridine/based diesters.[5,10–12]

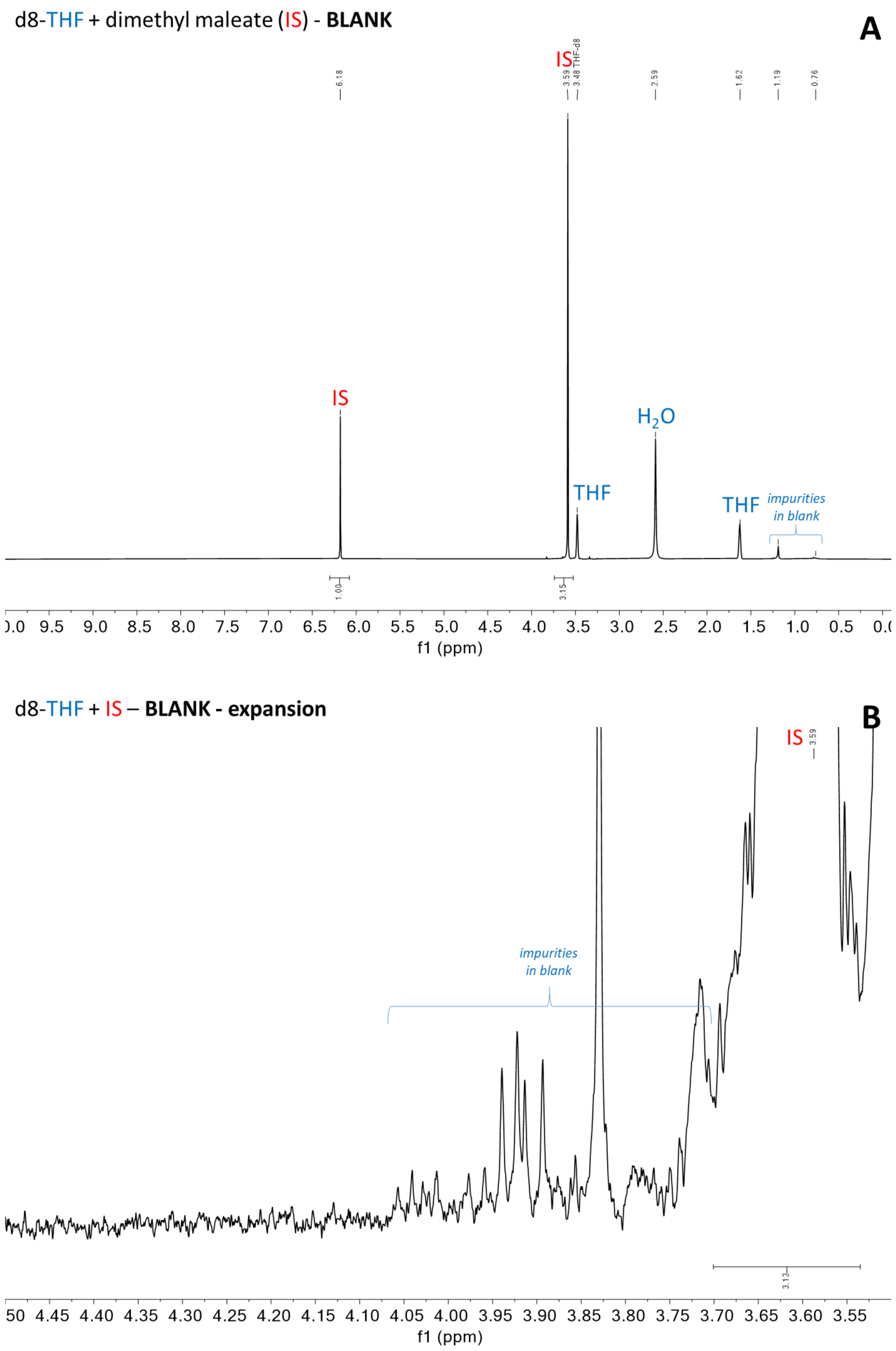

Supplementary Figure S37 ^{1}H-NMR spectrum of blank media for extraction studies of UV-cured ODO-PFAE polymer strips, showing THF-d_8 and dimethyl maleate was used as an external standard. **A**: full spectrum range, **B**: expansion of 4.5-3.5 ppm

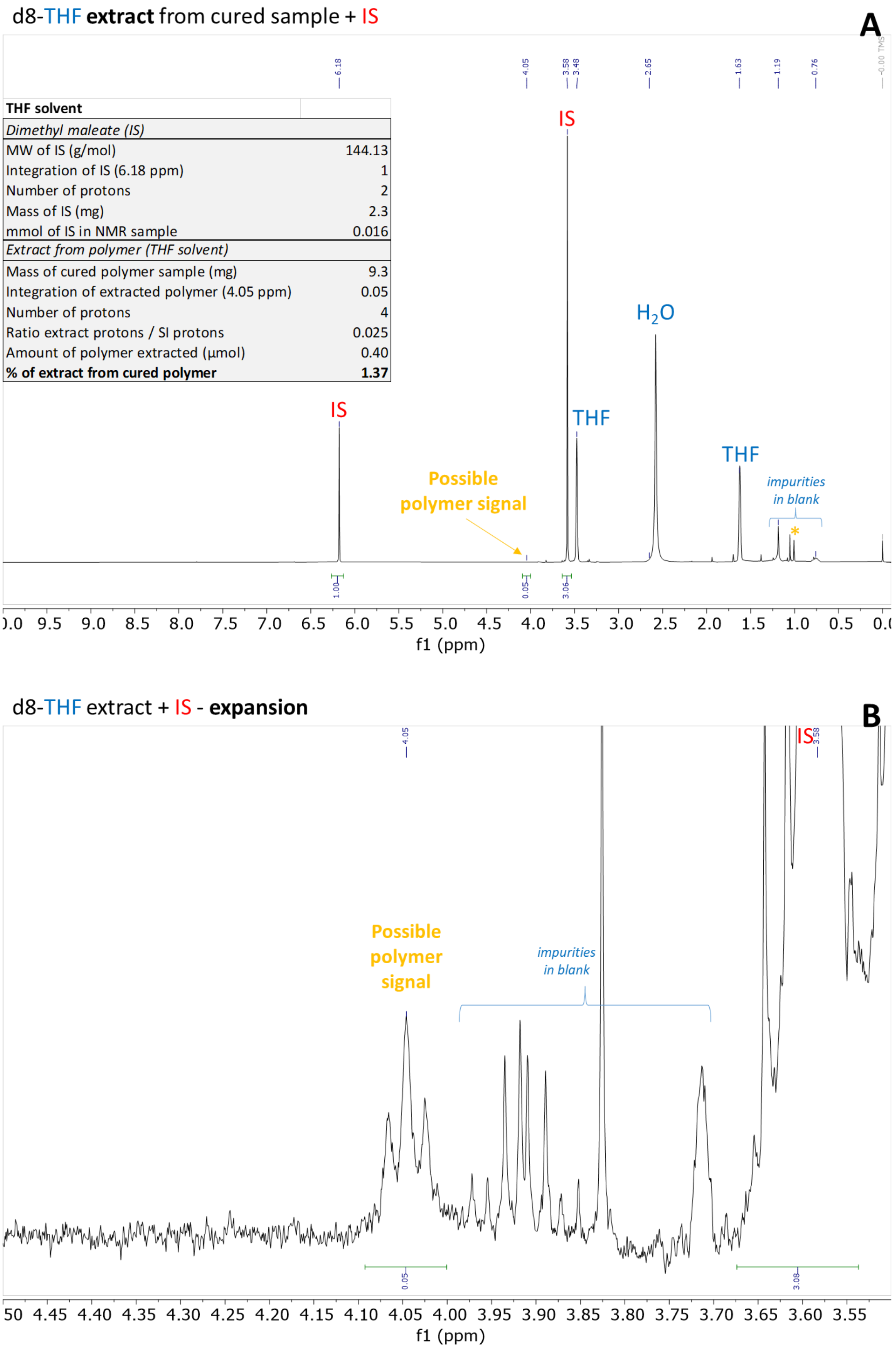

Supplementary Figure S38 ^{1}H-NMR spectrum of recovered solvent (THF-*d*$_8$) from extraction studies of UV-cured ODO-PFAE polymer strips (9.3 mg), dimethyl maleate was used as a standard (IS). **A**: full spectrum range and calculation of polymer extracted from strip using the CH_2 signal at 4.05 ppm, CRU of polymer assumed = 318 g/mol, **B**: expansion of 4.5-3.5 ppm showing signal and 4.05 ppm (see Figure S37 **B** for comparison)

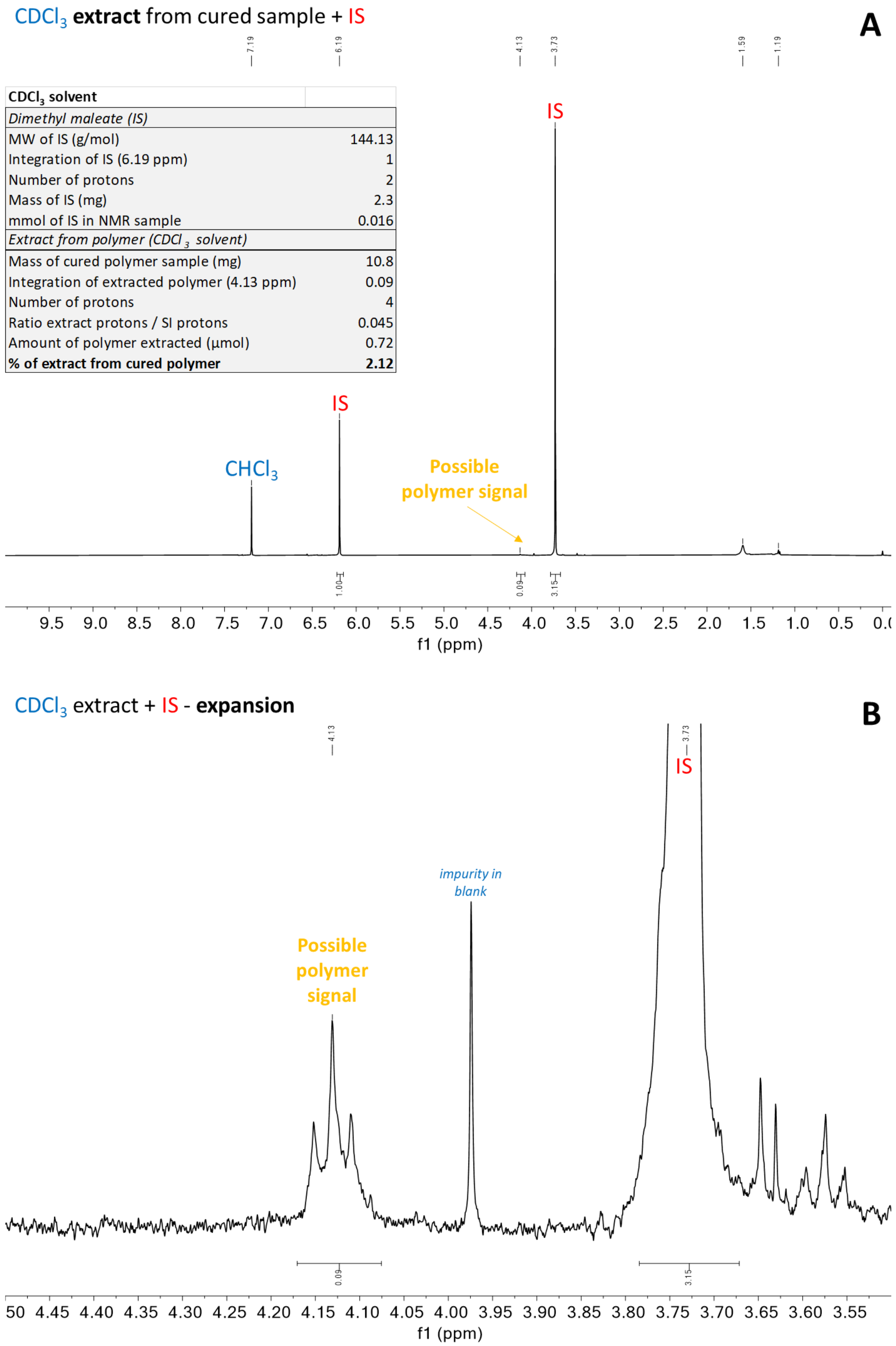

CDCl$_3$ solvent	
Dimethyl maleate (IS)	
MW of IS (g/mol)	144.13
Integration of IS (6.19 ppm)	1
Number of protons	2
Mass of IS (mg)	2.3
mmol of IS in NMR sample	0.016
Extract from polymer (CDCl$_3$ solvent)	
Mass of cured polymer sample (mg)	10.8
Integration of extracted polymer (4.13 ppm)	0.09
Number of protons	4
Ratio extract protons / SI protons	0.045
Amount of polymer extracted (µmol)	0.72
**% of extract from cured polymer**	**2.12**

Supplementary Figure S39 ^{1}H-NMR spectrum of recovered solvent ($CDCl_3$) from extraction studies of UV-cured ODO-PFAE polymer strips (10.8 mg), dimethyl maleate was used as a standard (IS). **A**: full spectrum range and calculation of polymer extracted from strip using the CH_2 signal at 4.13 ppm, CRU of polymer assumed = 318 g/mol, **B**: expansion of 4.5-3.5 ppm

Cartesian coordinates of TD-DFT calculated compounds

1

E = -840.3658832
G_{corr} = 0.177887

C	1.09619800	1.42414600	-0.00008900
C	0.70958700	2.74042700	-0.00018200
C	-0.70959100	2.74042400	-0.00018000
C	-1.09619400	1.42414000	-0.00010600
O	0.00000100	0.62219100	-0.00004600
H	1.37129800	3.59427300	-0.00023500
H	-1.37130600	3.59426700	-0.00023500
C	-2.40267900	0.81713600	-0.00007700
H	-3.23648400	1.51308400	-0.00012400
C	-2.63208200	-0.50676200	0.00000100
H	-1.82100500	-1.22683200	0.00005000
C	2.40268000	0.81714000	-0.00004000
H	3.23648800	1.51308500	-0.00007700
C	2.63208100	-0.50675900	0.00004400
H	1.82100400	-1.22682900	0.00008300
C	-3.98205000	-1.09876900	0.00002800
O	-4.18399300	-2.30084000	0.00011200
O	-4.96756800	-0.19177000	-0.00001100
C	3.98205000	-1.09876700	0.00009100
O	4.18399000	-2.30083900	0.00016900
O	4.96757000	-0.19177000	0.00005800
C	-6.30393000	-0.70558100	0.00003300
H	-6.47882500	-1.31052200	0.89211300
H	-6.95166000	0.16916000	-0.00000600
H	-6.47884700	-1.31061700	-0.89198000
C	6.30393100	-0.70558400	0.00010900
H	6.95166300	0.16915500	0.00005600
H	6.47882400	-1.31051200	0.89220000
H	6.47884500	-1.31063600	-0.89189300

1_{Dim}

E = -1680.752294
G_{corr} = 0.376274

C	-1.08843500	-0.58456000	1.64289700
C	-0.69851900	0.44936700	2.45564000
C	0.71980300	0.44712500	2.45143800
C	1.10165700	-0.58804300	1.63650600
O	0.00415800	-1.21593000	1.14097600
H	-1.35982700	1.12457400	2.97910300
H	1.38605900	1.12031200	2.97118600
C	2.40711400	-1.04680300	1.24286500
H	3.23753500	-0.50521000	1.68601100
C	2.64308500	-2.03789600	0.36742300
H	1.83626900	-2.58314300	-0.11001700
C	-2.39756000	-1.03836900	1.25608700
H	-3.22335700	-0.49547200	1.70613800
C	-2.64212600	-2.02509800	0.37810600
H	-1.84006100	-2.57065200	-0.10692800
C	3.99625200	-2.42185400	-0.06830900
O	4.20999800	-3.24832100	-0.93929700
O	4.97236200	-1.76448200	0.57099000
C	-3.99919600	-2.39941400	-0.05374300
O	-4.22106500	-3.21470700	-0.93322900
O	-4.96902400	-1.74575400	0.59875000
C	6.31123900	-2.04616900	0.15229400
H	6.43951400	-1.79394700	-0.90256900
H	6.94787500	-1.41860800	0.77343600
H	6.54627000	-3.10110700	0.30761700
C	-6.31120000	-2.01544200	0.18289900
H	-6.94194700	-1.39364600	0.81575600
H	-6.44339800	-1.74813000	-0.86776000
H	-6.55050100	-3.07131500	0.32472900
C	1.09807900	0.58968300	-1.63777600
C	0.71665500	-0.44606000	-2.45218800
C	-0.70166000	-0.45051000	-2.45427500
C	-1.09202200	0.58291500	-1.64113300
O	0.00029900	1.21592600	-1.14069300
H	1.38345000	-1.11825200	-2.97257500
H	-1.36243600	-1.12675500	-2.97703300
C	-2.40141000	1.03537200	-1.25348000
H	-3.22703000	0.48980100	-1.70068100
C	-2.64673100	2.02456300	-0.37847200
H	-1.84522600	2.57368300	0.10346200
C	2.40332900	1.04986700	-1.24526600
H	3.23380500	0.51155500	-1.69218000
C	2.63881200	2.03790800	-0.36627300
H	1.83159600	2.57918000	0.11499700
C	-4.00412400	2.39935200	0.05204500
O	-4.22675400	3.22126400	0.92514100
O	-4.97340600	1.73876600	-0.59428500
C	3.99182100	2.42153900	0.07016400
O	4.20509500	3.24179700	0.94713900
O	4.96831300	1.77094000	-0.57542400
C	-6.31566600	2.01017600	-0.17990600
H	-6.55609100	3.06473400	-0.32941700
H	-6.94603100	1.38315300	-0.80797400
H	-6.44721300	1.75034500	0.87273200
C	6.30730200	2.05239800	-0.15686500
H	6.94422600	1.43069600	-0.78357900
H	6.53985000	3.10891800	-0.30499200
H	6.43785100	1.79301800	0.89595500

2

E = -1680.768377
G_{corr} = 0.385329

C	-1.61542400	1.00890600	-1.72868300
C	-1.64843100	1.53373500	-2.98774000
C	-0.31588600	1.95329400	-3.28080800
C	0.43567200	1.66272400	-2.17459200
O	-0.35734100	1.08549800	-1.22942700
H	-2.51767800	1.60253100	-3.62524700
H	0.04458300	2.41785100	-4.18735800
C	1.82679100	1.86225500	-1.85752100
H	2.44955400	2.21761000	-2.67329100
C	2.36391000	1.63518700	-0.64613600
H	1.75786800	1.29989400	0.18864400
C	-2.61052900	0.33068100	-0.86672200

H	-3.61695400	0.53272000	-1.23776700
C	-2.47852800	0.56149900	0.66723900
H	-1.50839700	1.00089200	0.91463500
C	3.79200100	1.80637300	-0.33405500
O	4.25318400	1.65839700	0.78589800
O	4.54010600	2.13688400	-1.39361500
C	-3.55707000	1.38024700	1.32384300
O	-4.32756400	0.97326800	2.16878100
O	-3.56351300	2.62711300	0.84870500
C	5.94682000	2.25257400	-1.16240800
H	6.34982100	1.29571800	-0.82346500
H	6.37878200	2.52752100	-2.12308100
H	6.15124900	3.02333500	-0.41640000
C	-4.55523200	3.51387800	1.38630900
H	-4.40166100	4.46372800	0.87818500
H	-5.55615800	3.12794800	1.18380900
H	-4.41523700	3.62809700	2.46288700
C	0.69602500	-1.88888800	2.19616300
C	-0.00744400	-2.31645500	3.28952500
C	-1.38589000	-2.08216100	3.00242000
C	-1.42732200	-1.52003700	1.75996000
O	-0.17024100	-1.40131600	1.26455200
H	0.41525000	-2.75209300	4.18348000
H	-2.23744600	-2.29108500	3.63315300
C	-2.51115000	-0.96621900	0.91471700
H	-3.48046000	-1.27957700	1.30631100
C	-2.37649400	-1.19764900	-0.62223800
H	-1.36836700	-1.50150800	-0.90368800
C	2.10167400	-1.88174200	1.88174200
H	2.76581300	-2.17142300	2.69087000
C	2.60607800	-1.54633600	0.68130300
H	1.96230700	-1.27506500	-0.14842900
C	-3.34989200	-2.15048200	-1.26172700
O	-3.03464600	-3.10035600	-1.94952900
O	-4.61637300	-1.82866300	-0.98315900
C	4.04511100	-1.51024600	0.37591500
O	4.48719000	-1.26622000	-0.73488300
O	4.82765100	-1.76685000	1.43169200
C	-5.63218900	-2.67460200	-1.53805700
H	-5.51437100	-3.69575900	-1.17049200
H	-6.57741500	-2.25364000	-1.20123300
H	-5.57519000	-2.66740800	-2.62824000
C	6.23796500	-1.69188200	1.20611700
H	6.69958500	-1.93010300	2.16280400
H	6.54377600	-2.41182700	0.44410100
H	6.51412500	-0.68336300	0.89071200

3

E = -1680.761852

G_{corr} = 0.394756

C	-0.74782200	1.33074300	2.17672100
C	-1.72725400	1.48915600	3.11205800
C	-2.90580300	0.86887300	2.57473000
C	-2.55895800	0.37151900	1.35334000
O	-1.27489100	0.72711500	1.08027300
H	-1.60734100	1.92808100	4.09221500
H	-3.85976700	0.74243600	3.06640100
C	-3.10876300	-0.71852300	0.50117000
H	-4.19953300	-0.70858200	0.51979100
C	-2.54688500	-0.88173300	-0.92749100
H	-1.57188500	-0.39126200	-1.01013700
C	0.73669800	1.33615300	2.24659300
H	1.05098200	1.89202400	3.13116500
C	1.51034300	1.71886300	0.94627700
H	0.90717000	2.14756800	0.14717200
C	-3.35721300	-0.43736000	-2.11447700
O	-3.30487700	-0.96450000	-3.20780400
O	-4.10865800	0.62750300	-1.83740500
C	2.70025400	2.59805400	1.23496800
O	3.48660300	2.39899700	2.14166500
O	2.78698000	3.62635700	0.39367300
C	-4.88646000	1.15840100	-2.91960700
H	-5.58821400	0.40528200	-3.28316600
H	-5.42058600	2.01012900	-2.50329100
H	-4.23308700	1.47587900	-3.73446300
C	3.91188800	4.49880600	0.57044600
H	3.81158300	5.26162500	-0.19904600
H	3.88876600	4.95016300	1.56414600
H	4.84149600	3.94155500	0.43996100
C	0.47525700	-1.27534700	2.35484700
C	-0.01793200	-1.92347200	3.44852100
C	-1.24118000	-2.55116200	3.03335400
C	-1.40259400	-2.24542100	1.71447100
O	-0.32268400	-1.54287200	1.28621500
H	0.39630600	-1.89506500	4.44616600
H	-1.94063400	-3.09511600	3.65193500
C	-2.58623400	-2.19898000	0.81681300
H	-3.36815100	-2.83678400	1.23188000
C	-2.32605400	-2.40282500	-0.71227300
H	-1.33166900	-2.76397500	-0.97015600
C	1.39799100	-0.11996800	2.18075600
H	2.22251900	-0.17310700	2.89344500
C	1.87704500	0.22170200	0.75339100
H	1.20282200	-0.21649400	0.01103300
C	-3.33313000	-3.31095400	-1.36998400
O	-3.05650800	-4.31423200	-1.99322500
O	-4.58730900	-2.89433300	-1.15869600
C	3.27789600	-0.12439400	0.32804600
O	3.94045200	0.54455500	-0.43985700
O	3.69014700	-1.27312000	0.86142100
C	-5.63099900	-3.65919600	-1.77373700
H	-5.61869100	-4.68543100	-1.40176800
H	-6.55876800	-3.16285200	-1.49579600
H	-5.50548800	-3.65820700	-2.85829300
C	4.99935700	-1.72090800	0.48177600
H	5.15072500	-2.65870400	1.01242900
H	5.04610300	-1.87870200	-0.59740200
H	5.74912300	-0.98563300	0.77965500

S_2-S_1"C.I." (estimated)

E(S2) = -1680.639079

E(S1) = -1680.635432

C	-1.51479800	-0.24537400	-2.01796300
C	-1.10674600	-1.33932000	-2.78301600
C	0.28673300	-1.32252400	-2.79489100
C	0.68367600	-0.21261700	-2.04507100
O	-0.41971200	0.43514100	-1.58309000
H	-1.76805600	-2.05565100	-3.24817700

H	0.95621800	-2.01786300	-3.27996600
C	1.96177600	0.31389700	-1.74002300
H	2.81427800	-0.23243900	-2.13029300
C	2.14429900	1.44906000	-1.00372800
H	1.29852600	1.99651100	-0.60121500
C	-2.79269500	0.20072700	-1.61069000
H	-3.64125600	-0.36697500	-1.97906400
C	-2.98305800	1.27065200	-0.78311900
H	-2.14206100	1.83497700	-0.39561800
C	3.45262700	1.98741400	-0.64487600
O	3.60950700	2.96255900	0.07982900
O	4.47869700	1.30503400	-1.18005000
C	-4.29497400	1.71927800	-0.32089500
O	-4.45269100	2.60977000	0.50346700
O	-5.31987300	1.06248700	-0.88898400
C	5.78823200	1.75368100	-0.82547100
H	5.95239800	1.62863100	0.24748900
H	6.47507100	1.12467500	-1.38941000
H	5.92197300	2.80259700	-1.09800500
C	-6.62951100	1.45167100	-0.46883600
H	-7.31423100	0.78515700	-0.99082100
H	-6.73446300	1.33824500	0.61246700
H	-6.82507800	2.49050900	-0.74418000
C	1.51370300	0.24550300	2.01876100
C	1.10484900	1.33995100	2.78267200
C	-0.28862000	1.32249900	2.79406800
C	-0.68473900	0.21162300	2.04522700
O	0.41911300	-0.43601500	1.58422000
H	1.76564500	2.05705400	3.24737400
H	-0.95862800	2.01800500	3.27817800
C	-1.96246900	-0.31539800	1.73960400
H	-2.81543800	0.23113500	2.12857800
C	-2.14404900	-1.45100300	1.00373500
H	-1.29770900	-1.99872400	0.60278100
C	2.79193200	-0.20010100	1.61201100
H	3.64012100	0.36821800	1.98026600
C	2.98328700	-1.27011800	0.78479900
H	2.14289600	-1.83504500	0.39686100
C	-3.45186900	-1.98922200	0.64287500
O	-3.60765200	-2.96373400	-0.08294100
O	-4.47873400	-1.30733700	1.17713700
C	4.29585700	-1.71772900	0.32332500
O	4.45479700	-2.60777800	-0.50126000
O	5.31986500	-1.06066700	0.89269800
C	-5.78772500	-1.75439500	0.81847900
H	-5.92246000	-2.80414800	1.08720400
H	-6.47550600	-1.12693300	1.38297000
H	-5.94940200	-1.62549600	-0.25440600
C	6.63008200	-1.44878600	0.47336700
H	7.31406900	-0.78378100	0.99822300
H	6.82521000	-2.48842700	0.74593900
H	6.73653800	-1.33211200	-0.60744100

S_1Min

E = -1680.651923

G_{corr} = 0.380967

C	-1.81006900	-0.10697500	-1.67805200
C	-1.37945400	-1.15592600	-2.50901500
C	-0.00057400	-1.07021100	-2.58164700
C	0.38258300	0.03332300	-1.79807800
O	-0.73495600	0.61517800	-1.27202900
H	-2.02937300	-1.88653600	-2.96775900
H	0.68037400	-1.71677100	-3.11553000
C	1.64397700	0.57602800	-1.51334500
H	2.48999700	0.08430600	-1.98202900
C	1.85288600	1.63167500	-0.66017100
H	1.02502000	2.14812700	-0.18800200
C	-3.11419300	0.34126500	-1.32946600
H	-3.93478500	-0.26507000	-1.70033500
C	-3.35817500	1.41698800	-0.54407300
H	-2.55316400	2.03428700	-0.16057300
C	3.17656800	2.18141800	-0.38017300
O	3.38671800	3.11082700	0.38923200
O	4.16140200	1.54989800	-1.04404900
C	-4.70303900	1.79991700	-0.10345600
O	-4.92338800	2.71212200	0.67840300
O	-5.67692900	1.04390500	-0.63387300
C	5.49556700	1.96761000	-0.75094600
H	5.73403100	1.76421200	0.29596900
H	6.13605700	1.37527400	-1.40236000
H	5.61978300	3.03273400	-0.95692900
C	-7.01136300	1.33878700	-0.21342100
H	-7.64342100	0.61019000	-0.71845200
H	-7.10141800	1.23617000	0.87030500
H	-7.28943700	2.35328600	-0.50699300
C	1.81935300	0.08785900	1.70055800
C	1.38863100	1.13677900	2.53154500
C	0.00975500	1.05105600	2.60396700
C	-0.37330900	-0.05238100	1.82019200
O	0.74433000	-0.63424000	1.29433600
H	2.03854500	1.86737400	2.99032100
H	-0.67131100	1.69753900	3.13779600
C	-1.63462100	-0.59513000	1.53521700
H	-2.48076600	-0.10330200	2.00356700
C	-1.84330900	-1.65088900	0.68212700
H	-1.01530000	-2.16748100	0.21030900
C	3.12354700	-0.36014800	1.35189700
H	3.94403300	0.24613300	1.72306000
C	3.36755000	-1.43541000	0.56584200
H	2.56252000	-2.05248000	0.18201600
C	-3.16688900	-2.20068500	0.40174900
O	-3.37666900	-3.13047800	-0.36728900
O	-4.15200100	-1.56879700	1.06483100
C	4.71237400	-1.81825500	0.12514600
O	4.93271000	-2.73045300	-0.65676500
O	5.68627400	-1.06218300	0.65542700
C	-5.48607700	-1.98652600	0.77128700
H	-5.61036500	-3.05163900	0.97725900
H	-6.12677800	-1.39414700	1.42245000
H	-5.72418000	-1.78317000	-0.27572200
C	7.02072300	-1.35721800	0.23508300
H	7.65276400	-0.62845200	0.73988900
H	7.29876600	-2.37162100	0.52901800
H	7.11078600	-1.25497000	-0.84867300

S_1TS

E = -1680.625789

G_{corr} = 0.379776

C	-1.26298400	1.09006700	-1.69385800
C	-0.97141900	0.95036400	-3.04597800
C	0.42344900	1.06397300	-3.16694400
C	0.91752000	1.27053600	-1.89219000
O	-0.10438400	1.28948700	-1.00419300
H	-1.69524500	0.77710500	-3.82890300
H	1.01648000	1.00516600	-4.06805200
C	2.25033900	1.45224500	-1.39785200
H	3.04445100	1.39889800	-2.13621100
C	2.53457400	1.65156800	-0.09631400
H	1.74958300	1.69833200	0.65079700
C	-2.46135600	1.00056200	-0.96397200
H	-3.37991000	0.93942800	-1.53581600
C	-2.51691200	1.26598900	0.43646400
H	-1.61299800	1.53550000	0.96816900
C	3.89565400	1.78587100	0.44312800
O	4.12745000	1.97388900	1.62585400
O	4.85801700	1.67208600	-0.48163100
C	-3.75940800	1.54328200	1.13780700
O	-3.83238500	1.79402700	2.33588000
O	-4.84197400	1.46077500	0.34305600
C	6.20252800	1.81152600	-0.01137200
H	6.41203700	1.07375900	0.76588600
H	6.83486500	1.64319500	-0.88132400
H	6.36250700	2.81537800	0.38792200
C	-6.11257400	1.58423700	0.98264200
H	-6.85046000	1.48601300	0.18790400
H	-6.24358200	0.79074200	1.72250900
H	-6.20599500	2.55666700	1.47101000
C	0.99587900	-1.20643200	1.88067300
C	0.49994400	-0.97322900	3.14645000
C	-0.90002200	-0.87460100	3.01965500
C	-1.18474800	-1.05170000	1.67670300
O	-0.02629800	-1.25365800	0.99155200
H	1.09016200	-0.88840200	4.04731700
H	-1.62695500	-0.68976400	3.79696000
C	-2.39636800	-1.00271500	0.94097200
H	-3.30891300	-0.99215900	1.52587700
C	-2.45163600	-1.38444500	-0.42918600
H	-1.53995600	-1.60507600	-0.96988700
C	2.33249000	-1.39634100	1.39459000
H	3.12120900	-1.32952300	2.13776000
C	2.62816700	-1.62084200	0.10063700
H	1.85077800	-1.68023600	-0.65337000
C	-3.68750700	-1.68217300	-1.12197900
O	-3.76125200	-1.98877300	-2.30874400
O	-4.77402300	-1.56211700	-0.33293300
C	3.99445100	-1.77554100	-0.42105800
O	4.23801400	-1.98911000	-1.59691500
O	4.94783700	-1.65385700	0.51234500
C	-6.04266600	-1.70458800	-0.97042300
H	-6.13814500	-2.69264300	-1.42612600
H	-6.78259700	-1.57770800	-0.18151700
H	-6.17103800	-0.93632000	-1.73716700
C	6.29559300	-1.82581300	0.06230800
H	6.91994300	-1.64799300	0.93617900
H	6.44360000	-2.84111200	-0.31186000
H	6.52798700	-1.10989300	-0.72875000

S_1Min2

E = -1680.67073

G_{corr} = 0.386201

C	-1.84849200	1.78715100	-1.61241300
C	-1.88312200	2.09116400	-2.98537600
C	-0.55994400	2.05652400	-3.43870200
C	0.23615900	1.74889100	-2.34844800
O	-0.54191900	1.60001100	-1.24657100
H	-2.77611400	2.29011700	-3.55818800
H	-0.20401300	2.22941700	-4.44394100
C	1.65407200	1.61596900	-2.18751400
H	2.24634400	1.67871100	-3.09522200
C	2.25185100	1.44170600	-0.99156000
H	1.67591400	1.40710000	-0.07314800
C	-2.81830800	1.50641300	-0.67568000
H	-3.86021000	1.62180700	-0.94456400
C	-2.42413300	0.94907700	0.65766300
H	-1.45025000	1.35189400	0.94252000
C	3.70898100	1.35063000	-0.80280400
O	4.24064700	1.40295200	0.29405700
O	4.39405000	1.20974800	-1.94273400
C	-3.38669200	1.35427500	1.75724300
O	-3.07594000	2.00561000	2.73231900
O	-4.62240100	0.90627200	1.52866200
C	5.81459800	1.07655500	-1.81250000
H	6.04929800	0.19077500	-1.21854100
H	6.18981800	0.96815500	-2.82869400
H	6.23878500	1.96619000	-1.34200000
C	-5.61610700	1.23379700	2.50981000
H	-6.53980800	0.78628400	2.14859800
H	-5.33991400	0.81504400	3.47941300
H	-5.72085100	2.31712300	2.59315100
C	0.72181900	-1.44104800	2.39933500
C	-0.11757600	-1.62662700	3.46325700
C	-1.44292300	-1.40286100	2.97895600
C	-1.31690900	-1.08754600	1.65861100
O	-0.01055400	-1.10515200	1.30102000
H	0.17936000	-1.90423000	4.46433200
H	-2.36728700	-1.46232100	3.53488600
C	-2.27755500	-0.63910400	0.60385800
H	-3.26221200	-1.06511200	0.81365000
C	-1.83670200	-1.06678100	-0.75461100
H	-0.78640100	-1.18847000	-0.97462600
C	2.14787500	-1.57562100	2.23514600
H	2.71689800	-1.73607300	3.14614600
C	2.77054800	-1.52744900	1.04531300
H	2.21601200	-1.39473700	0.12223500
C	-2.73529500	-1.38627800	-1.82879100
O	-2.36276300	-1.65573000	-2.96640900
O	-4.03598900	-1.34888800	-1.47685400
C	4.22397400	-1.67114500	0.86702300
O	4.75373300	-1.78557100	-0.22607000
O	4.91270100	-1.66849600	2.01472900
C	-4.97655000	-1.59787600	-2.52201000
H	-4.82373100	-2.59177500	-2.94825500
H	-5.95789400	-1.53165800	-2.05546000
H	-4.87784900	-0.84671900	-3.30926300
C	6.33419200	-1.78291800	1.89858400
H	6.71331400	-1.75329200	2.91856200
H	6.60410600	-2.72544000	1.41751400
H	6.73375600	-0.94803900	1.31883800

1-2TS

E = -1680.662304

G_{corr} = 0.380844

C	-0.80381600	0.84611300	-1.56727000
C	-0.33065900	0.72805200	-2.89822400
C	0.99126100	1.11444100	-2.89819200
C	1.29656100	1.44041400	-1.56787800
O	0.24109500	1.29996200	-0.78206600
H	-0.92686500	0.38636500	-3.73291700
H	1.68622300	1.14105200	-3.72339600
C	2.56935600	1.79903900	-0.99218300
H	3.38173300	1.96643000	-1.69053700
C	2.76822300	1.81865700	0.33329100
H	1.96532900	1.62405200	1.03697200
C	-2.02196700	0.56793500	-1.04340400
H	-2.76220800	0.18526000	-1.73857500
C	-2.42252300	0.67402400	0.37063800
H	-1.69076100	1.21924500	0.97013900
C	4.09625100	2.05538000	0.95891000
O	4.24753800	2.10461700	2.16266600
O	5.07718800	2.18451000	0.06940300
C	-3.75443100	1.37917700	0.54680400
O	-4.17071900	1.72017800	1.63248200
O	-4.39553300	1.58102000	-0.60234400
C	6.39585600	2.38623200	0.60035700
H	6.67813900	1.54041900	1.23013200
H	7.04894500	2.45499300	-0.26693900
H	6.42953900	3.30854500	1.18273300
C	-5.71269600	2.14013500	-0.50583800
H	-6.08120100	2.19325100	-1.52819900
H	-6.34526000	1.48730500	0.09765700
H	-5.66927100	3.13492800	-0.05879200
C	0.89906700	-1.56353200	1.57921300
C	0.50537400	-1.63020500	2.88950700
C	-0.89662500	-1.38524300	2.90417400
C	-1.26754400	-1.18924000	1.60309100
O	-0.18781600	-1.30216900	0.79358300
H	1.15266700	-1.81915800	3.73407400
H	-1.55279500	-1.34623600	3.76101500
C	-2.56532900	-0.83830200	0.96678900
H	-3.28535200	-0.72511400	1.78236100
C	-3.02660400	-1.85987900	-0.02218500
H	-2.28709500	-2.41578900	-0.58757100
C	2.18835400	-1.61468200	0.95452500
H	3.03626500	-1.69130100	1.62945100
C	2.38848100	-1.50763300	-0.37291200
H	1.55318500	-1.44721800	-1.06274700
C	-4.36644100	-2.05975500	-0.32636800
O	-4.87002000	-2.84519000	-1.16260700
O	-5.22458100	-1.25115500	0.42903600
C	3.70529000	-1.34853700	-0.99651700
O	3.85513800	-1.11012300	-2.18576500
O	4.73036700	-1.43517200	-0.13771100
C	-6.60958800	-1.41798100	0.19115800
H	-6.92596600	-2.45267200	0.35398100
H	-7.11724300	-0.76455700	0.90437400
H	-6.87960400	-1.12898800	-0.83046400
C	6.03537900	-1.21612100	-0.68283600

H	6.72074300	-1.30887500	0.15785300
H	6.26260800	-1.96484300	-1.44433000
H	6.09911600	-0.21731400	-1.12058500

T_1Min

E = -1680.669808

G_{corr} = 0.381015

C	-1.82537900	-0.08737900	-1.67136800
C	-1.38761400	-1.13959600	-2.50333800
C	-0.01469000	-1.05558500	-2.57047700
C	0.37148700	0.05110000	-1.78002400
O	-0.74920300	0.63291500	-1.25777100
H	-2.03669700	-1.86860700	-2.96623500
H	0.66816100	-1.70196800	-3.10241300
C	1.63053300	0.58198600	-1.50048300
H	2.47113000	0.08388100	-1.97243800
C	1.85670500	1.63970900	-0.64763200
H	1.03728100	2.16435300	-0.17031500
C	-3.12888600	0.35667600	-1.33441100
H	-3.94543800	-0.24832400	-1.71633800
C	-3.38383900	1.42517200	-0.53920000
H	-2.58421800	2.04105400	-0.14282700
C	3.18690800	2.18106900	-0.38061200
O	3.40679200	3.10950900	0.38600500
O	4.16205800	1.54386700	-1.05085900
C	-4.73344100	1.80076100	-0.10695100
O	-4.96255700	2.70962500	0.67590400
O	-5.70032700	1.04164300	-0.64501500
C	5.50096400	1.95694500	-0.77093900
H	5.74556000	1.76139200	0.27588500
H	6.13318400	1.35549900	-1.42199000
H	5.62908000	3.01945300	-0.98756000
C	-7.03908000	1.33136900	-0.23442800
H	-7.66453100	0.60053900	-0.74438500
H	-7.13691800	1.22819000	0.84852700
H	-7.31867400	2.34486000	-0.52995600
C	1.83463600	0.06814100	1.69371600
C	1.39667100	1.12015000	2.52578900
C	0.02372300	1.03607800	2.59261200
C	-0.36224100	-0.07050000	1.80189400
O	0.75859300	-0.65214200	1.27975200
H	2.04562500	1.84909900	2.98896900
H	-0.65926700	1.68231000	3.12455400
C	-1.62119100	-0.60138100	1.52206400
H	-2.46188800	-0.10323200	1.99379500
C	-1.84725000	-1.65911400	0.66913200
H	-1.02773900	-2.18399200	0.19222400
C	3.13823500	-0.37555400	1.35661500
H	3.95466200	0.22936400	1.73894300
C	3.39341000	-1.44367200	0.56096000
H	2.59389500	-2.05943100	0.16418000
C	-3.17745400	-2.20035500	0.40185700
O	-3.39733000	-3.12849500	-0.36512700
O	-4.15261900	-1.56337500	1.07230900
C	4.74306700	-1.81884300	0.12856900
O	4.97231000	-2.72709000	-0.65497400
O	5.70986300	-1.05998400	0.66717200
C	-5.49158200	-1.97589300	0.79180100
H	-5.61951400	-3.03901700	1.00541600

H	-6.12361800	-1.37630900	1.44474500
H	-5.73657500	-1.77723900	-0.25435300
C	7.04865900	-1.34941500	0.25653400
H	7.67408400	-0.61917000	0.76735600
H	7.32811200	-2.36324300	0.55102600
H	7.14668200	-1.24508400	-0.82629300

T_1TS

E = -1680.663162

G_{corr} = 0.373947

C	-0.95299400	1.08571300	-1.65094000
C	-0.50055800	0.87281300	-2.94957500
C	0.89644500	0.99719100	-2.91463200
C	1.23796400	1.28135600	-1.60514300
O	0.11571300	1.33481400	-0.84028300
H	-1.12703000	0.64974400	-3.80094100
H	1.59276600	0.88990200	-3.73377500
C	2.51274500	1.48893900	-0.99466200
H	3.36542400	1.41325900	-1.66312300
C	2.70371800	1.71837000	0.32087100
H	1.87173000	1.77619600	1.01434200
C	-2.25198000	1.08192700	-1.10915300
H	-3.06425700	0.93009100	-1.81148400
C	-2.52743500	1.23210800	0.24909900
H	-1.74046400	1.53588400	0.92893600
C	4.03171000	1.81500400	0.94032200
O	4.20417100	1.90267400	2.14568600
O	5.04245900	1.77949600	0.06043400
C	-3.88583900	1.52444500	0.74318000
O	-4.12920800	1.86093100	1.89031900
O	-4.83121600	1.36647300	-0.19078800
C	6.36206400	1.81854100	0.61049100
H	6.51709300	0.97488900	1.28665900
H	7.03560900	1.75245700	-0.24227900
H	6.52313900	2.75322200	1.15178400
C	-6.18166700	1.56498700	0.23705200
H	-6.79633200	1.38122900	-0.64235900
H	-6.43160900	0.86128400	1.03408500
H	-6.32212500	2.58711000	0.59417000
C	0.99514400	-1.18343900	1.62847300
C	0.65939300	-0.76828800	2.90743900
C	-0.73377600	-0.63241400	2.93783600
C	-1.19522900	-0.97455700	1.66653400
O	-0.13291700	-1.31575500	0.88244400
H	1.36096400	-0.58307000	3.70815700
H	-1.35422600	-0.31671000	3.76386900
C	-2.47201500	-0.91815500	1.08373100
H	-3.30146600	-0.78397900	1.76832300
C	-2.72510700	-1.53652400	-0.20217100
H	-1.89974000	-1.77465500	-0.86180100
C	2.26142000	-1.41805200	1.01771300
H	3.12473400	-1.26030800	1.65729000
C	2.43276800	-1.74659000	-0.28140500
H	1.58984100	-1.88886100	-0.94882300
C	-4.04863700	-1.85579000	-0.69610600
O	-4.27095500	-2.26281500	-1.83084800
O	-5.01632000	-1.66898000	0.21941000
C	3.74996700	-1.81841600	-0.92367200
O	3.90543300	-1.98765600	-2.12299400
O	4.77343700	-1.64956300	-0.07359200
C	-6.35062700	-1.94660900	-0.21123900
H	-6.44385500	-2.99063000	-0.51784500
H	-6.98451500	-1.74576000	0.65084300
H	-6.62338900	-1.29711400	-1.04634200
C	6.08015400	-1.62296400	-0.65332500
H	6.76779800	-1.50415600	0.18234000
H	6.28408800	-2.55472000	-1.18476100
H	6.16939300	-0.78061900	-1.34329500

T_1Min2

E = -1680.700312

G_{corr} = 0.378717

C	-0.57568300	1.12215000	-1.56901700
C	0.14056900	1.10654100	-2.79923600
C	1.45427600	1.42986700	-2.50734300
C	1.53344100	1.63231500	-1.12630900
O	0.30111500	1.46727000	-0.56896600
H	-0.28948700	0.86867300	-3.76128000
H	2.28800600	1.49492800	-3.19201600
C	2.64760200	1.87858400	-0.28632300
H	3.60210500	1.99343600	-0.79100000
C	2.58168900	1.93181200	1.06849100
H	1.64097200	1.80025400	1.59149900
C	-1.87967200	0.83752900	-1.28543200
H	-2.51325300	0.54043600	-2.11300900
C	-2.46365200	0.88246300	0.09335200
H	-1.90036900	1.57291800	0.72587400
C	3.74923100	2.11563500	1.93484800
O	3.68836000	2.11853400	3.15495900
O	4.90166400	2.27181700	1.26447000
C	-3.90088000	1.38014300	0.11349300
O	-4.35823100	2.06334600	1.00587100
O	-4.61279900	0.94374200	-0.92459100
C	6.07958300	2.44210200	2.05801200
H	6.23748400	1.57175500	2.69858300
H	6.89751600	2.54417200	1.34685900
H	5.99777400	3.33897300	2.67577700
C	-6.00450700	1.28886500	-0.93493000
H	-6.41980900	0.79566700	-1.81140000
H	-6.48584400	0.92827200	-0.02388900
H	-6.12314300	2.37133200	-1.00979800
C	1.06862200	-1.33260800	1.01935500
C	0.92428200	-1.03597600	2.34699100
C	-0.44723000	-0.70100200	2.53897800
C	-1.04056700	-0.81813200	1.31588100
O	-0.13612200	-1.21668900	0.39247000
H	1.71409500	-1.03574500	3.08444200
H	-0.93309200	-0.39890300	3.45512600
C	-2.42802900	-0.51580400	0.85117700
H	-3.03688700	-0.39086700	1.75117700
C	-2.99035200	-1.61791600	0.01393600
H	-2.37120400	-2.09584400	-0.73696500
C	2.23046800	-1.62836200	0.22601700
H	3.16187400	-1.70972500	0.77869200
C	2.22693400	-1.74815400	-1.11272600
H	1.31350000	-1.64792000	-1.68916900
C	-4.36119600	-2.08023300	0.09485900
O	-4.82346600	-2.96028000	-0.62011800

O	-5.08026300	-1.44558900	1.03745600
C	3.44063400	-1.95765300	-1.91491400
O	3.43883400	-2.00422100	-3.13437200
O	4.55771100	-2.08022100	-1.18378600
C	-6.44498700	-1.85225200	1.17504700
H	-6.50050700	-2.90108500	1.47435000
H	-6.86266600	-1.21206400	1.95005800
H	-6.98055700	-1.71449300	0.23299300
C	5.77587900	-2.26618900	-1.91093100
H	6.55631400	-2.34431400	-1.15608400
H	5.72895300	-3.17990400	-2.50710700
H	5.96276300	-1.41249000	-2.56590200

1 (B3LYP-D3(BJ))

E = -840.695356

G_{corr} = 0.175332

C	1.10674100	1.41422300	-0.00010500
C	0.70813900	2.73746200	-0.00019100
C	-0.70918000	2.73683200	-0.00016800
C	-1.10654500	1.41329800	-0.00008800
O	0.00040700	0.60575100	-0.00004300
H	1.36720900	3.59365500	-0.00025200
H	-1.36927300	3.59224300	-0.00021100
C	-2.40775600	0.81511900	-0.00004100
H	-3.23775400	1.51501000	-0.00007700
C	-2.65186200	-0.51493200	0.00004400
H	-1.84486300	-1.23906700	0.00008200
C	2.40828700	0.81645400	-0.00008700
H	3.23850000	1.51619300	-0.00013100
C	2.65213800	-0.51365400	-0.00002700
H	1.84481500	-1.23743500	0.00001900
C	-4.00047400	-1.09777100	0.00009300
O	-4.21395300	-2.30448200	0.00014700
O	-4.98950200	-0.17809700	0.00001500
C	4.00039800	-1.09735000	-0.00002600
O	4.21276900	-2.30426600	0.00011500
O	4.99011100	-0.17843100	0.00005300
C	-6.33605600	-0.69157800	0.00002700
H	-6.51115600	-1.29679000	0.89228800
H	-6.97986500	0.18678500	-0.00003600
H	-6.51113100	-1.29690000	-0.89216400
C	6.33643600	-0.69263100	0.00017000
H	6.98064800	0.18543500	0.00014600
H	6.51125500	-1.29781100	0.89250900
H	6.51136100	-1.29794300	-0.89205800

1_{Dim} (B3LYP-D3(BJ))

E = -1681.41090265

G_{corr} = 0.370277

C	-1.10491500	0.56458400	-1.69457700
C	-0.70719900	-0.45457600	-2.53868300
C	0.70997500	-0.45466400	-2.53857200
C	1.10768100	0.56439000	-1.69433500
O	0.00138400	1.18640000	-1.17683200
H	-1.36838700	-1.11822500	-3.07707600
H	1.37116800	-1.11844200	-3.07680000
C	2.40918300	1.00185800	-1.29498200
H	3.23387100	0.46347300	-1.75058300
C	2.66279400	1.97460100	-0.39172400
H	1.86210500	2.51418400	0.10088700
C	-2.40642100	1.00250800	-1.29574600
H	-3.23108400	0.46341700	-1.75054400
C	-2.66006300	1.97633800	-0.39366900
H	-1.85938100	2.51661900	0.09818000
C	4.01523900	2.32768300	0.05510200
O	4.24250800	3.12693000	0.95684700
O	4.99229100	1.67378600	-0.60888600
C	-4.01250900	2.32966700	0.05294500
O	-4.23984500	3.13017400	0.95355500
O	-4.98948200	1.67450100	-0.60991000
C	6.34079500	1.92840300	-0.17317500
H	6.46231400	1.63337400	0.87160900
H	6.97248500	1.31993900	-0.81863200
H	6.58403400	2.98730400	-0.28474000
C	-6.33798900	1.92932300	-0.17439200
H	-6.96962500	1.32025300	-0.81933100
H	-6.45946500	1.63510400	0.87063000
H	-6.58137200	2.98810100	-0.28679400
C	1.10495700	-0.56547400	1.69543900
C	0.70725600	0.45261200	2.54084000
C	-0.70991900	0.45261900	2.54086600
C	-1.10764800	-0.56541200	1.69539700
O	-0.00135500	-1.18669000	1.17699500
H	1.36845200	1.11569400	3.07991700
H	-1.37107800	1.11560400	3.08011500
C	-2.40916700	-1.00256500	1.29574800
H	-3.23382900	-0.46438600	1.75164000
C	-2.66282200	-1.97455400	0.39169100
H	-1.86213700	-2.51365100	-0.10144800
C	2.40646300	-1.00297500	1.29611800
H	3.23117500	-0.46505800	1.75222400
C	2.66001600	-1.97530800	0.39240300
H	1.85926800	-2.51428800	-0.10075500
C	-4.01526300	-2.32725600	-0.05542100
O	-4.24254500	-3.12598300	-0.95762600
O	-4.99228600	-1.67356400	0.60881000
C	4.01244800	-2.32844600	-0.05438200
O	4.23970500	-3.12732200	-0.95646200
O	4.98950900	-1.67491200	0.60994700
C	-6.34078600	-1.92782800	0.17293700
H	-6.58421700	-2.98672400	0.28416400
H	-6.97242500	-1.31945900	0.81853700
H	-6.46220600	-1.63246700	-0.87176500
C	6.33802100	-1.92941900	0.17420600
H	6.96972700	-1.32147100	0.82013400
H	6.58111300	-2.98842700	0.28507000
H	6.45967600	-1.63367900	-0.87036200

1 (CAMB3LYP-D3(BJ))

E = -840.2819783

G_{corr} = 0.178322

C	1.09917100	1.42757900	-0.00021800
C	0.70955400	2.74151400	-0.00020900
C	-0.70955100	2.74151600	-0.00010200
C	-1.09916700	1.42758000	-0.00004200
O	-0.00000300	0.62432700	-0.00011600
H	1.36976000	3.59654900	-0.00027600

H	-1.36975300	3.59655400	-0.00006800
C	-2.40191800	0.81845900	0.00007500
H	-3.23904300	1.50919200	0.00013000
C	-2.62683300	-0.50442700	0.00012200
H	-1.81394400	-1.22138100	0.00006600
C	2.40191500	0.81844700	-0.00030700
H	3.23904300	1.50917700	-0.00038800
C	2.62682800	-0.50443900	-0.00031700
H	1.81394200	-1.22139600	-0.00023100
C	-3.97280000	-1.09731300	0.00025400
O	-4.17603400	-2.29918800	0.00017400
O	-4.96232400	-0.19287800	0.00017500
C	3.97280200	-1.09731500	-0.00046500
O	4.17604400	-2.29919000	-0.00000600
O	4.96232000	-0.19287500	0.00001300
C	-6.29804800	-0.71315100	0.00014400
H	-6.46838700	-1.31943100	0.89138700
H	-6.95018900	0.15787400	0.00008300
H	-6.46831900	-1.31950400	-0.89106300
C	6.29804500	-0.71314300	0.00049500
H	6.95017700	0.15788800	0.00081600
H	6.46800600	-1.31952900	0.89173900
H	6.46869400	-1.31939100	-0.89071000

1_{Dim} (CAMB3LYP-D3(BJ))

E = -1680.582904

G_{corr} = 0.375697

C	-1.09999800	0.64457200	-1.70206200
C	-0.71058800	-0.32808200	-2.58540900
C	0.70818500	-0.32810600	-2.58557100
C	1.09783700	0.64456600	-1.70235400
O	-0.00101700	1.23873200	-1.15996500
H	-1.37194900	-0.96517900	-3.15443100
H	1.36938900	-0.96523900	-3.15473600
C	2.40136300	1.07541900	-1.28111400
H	3.23251300	0.56084700	-1.75186700
C	2.63790700	2.01478700	-0.35333700
H	1.83274500	2.53638400	0.15070900
C	-2.40342600	1.07537500	-1.28044400
H	-3.23472500	0.56130200	-1.75148800
C	-2.63972000	2.01449000	-0.35234300
H	-1.83442700	2.53558600	0.15200000
C	3.98927500	2.36823900	0.10213600
O	4.20872400	3.15049000	1.01177300
O	4.96592200	1.73686500	-0.56456300
C	-3.99100100	2.36818800	0.10322100
O	-4.21025800	3.15046100	1.01288200
O	-4.96780600	1.73696900	-0.56339700
C	6.30564800	1.99865500	-0.12997100
H	6.43164500	1.69255400	0.91012300
H	6.94478800	1.40669500	-0.78206700
H	6.53675700	3.06059000	-0.22801900
C	-6.30746100	1.99903000	-0.12874800
H	-6.94674300	1.40715800	-0.78078400
H	-6.43345900	1.69298800	0.91136400
H	-6.53837400	3.06100500	-0.22681700
C	1.09961100	-0.64597800	1.70367500
C	0.70982600	0.32519700	2.58848000
C	-0.70894100	0.32475600	2.58857200
C	-1.09823200	-0.64662700	1.70376200
O	0.00084100	-1.23956500	1.16048900
H	1.37097900	0.96150600	3.15861800
H	-1.37040300	0.96071200	3.15874900
C	-2.40161500	-1.07727600	1.28183900
H	-3.23302500	-0.56501100	1.75464100
C	-2.63776200	-2.01402900	0.35132200
H	-1.83236200	-2.53284000	-0.15519200
C	2.40323000	-1.07590200	1.28175700
H	3.23429500	-0.56248100	1.75392000
C	2.63993800	-2.01314100	0.35187200
H	1.83484500	-2.53312600	-0.15392800
C	-3.98901600	-2.36733200	-0.10459300
O	-4.20816700	-3.14708400	-1.01644800
O	-4.96593500	-1.73884000	0.56442000
C	3.99137600	-2.36562400	-0.10412200
O	4.21092300	-3.14501500	-1.01619200
O	4.96796500	-1.73679800	0.56505000
C	-6.30562300	-2.00057500	0.12967500
H	-6.53571200	-3.06300800	0.22466900
H	-6.94499600	-1.41111900	0.78380900
H	-6.43237000	-1.69150500	-0.90945100
C	6.30776500	-1.99754900	0.13005600
H	6.94683200	-1.40764100	0.78408000
H	6.53864100	-3.05981600	0.22501500
H	6.43409900	-1.68840600	-0.909097

Supplementary references

[1] F. Byrne, B. Forier, G. Bossaert, C. Hoebers, T. J. Farmer, J. H. Clark, A. J. Hunt, *Green Chem.* **2017**, *19*, 3671–3678.

[2] A. Pellis, L. Corici, L. Sinigoi, N. D'Amelio, D. Fattor, V. Ferrario, C. Ebert, L. Gardossi, *Green Chem.* **2015**, *17*, 1756–1766.

[3] E. M. Serum, S. Selvakumar, N. Zimmermann, M. P. Sibi, *Green Chem.* **2018**, *20*, 1448–1454.

[4] W. F. Cooper, W. H. Nuttall, *J. Chem. Soc., Trans.* **1914**, *105*, 2218–2226.

[5] A. Pellis, J. W. Comerford, S. Weinberger, G. M. Guebitz, J. H. Clark, T. J. Farmer, *Nat. Commun.* **2019**, *10*, 1762.

[6] M. J. Frisch, G. W. Trucks, H. B. Schlegel, G. E. Scuseria, M. A. Robb, J. R. Cheeseman, G. Scalmani, V. Barone, G. A. Petersson, H. Nakatsuji, et al., **2016**.

[7] J. Da Chai, M. Head-Gordon, *Phys. Chem. Chem. Phys.* **2008**, *10*, 6615–6620.

[8] M. Cossi, N. Rega, G. Scalmani, V. Barone, *J. Comput. Chem.* **2003**, *24*, 669–681.

[9] S. Raza, L. Fransson, K. Hult, *Protein Sci.* **2001**, *10*, 329–338.

[10] A. Pellis, A. Guarneri, M. Brandauer, E. H. Acero, H. Peerlings, L. Gardossi, G. M. Guebitz, *Biotechnol. J.* **2016**, *11*, 642–647.

[11] Y. Jiang, A. J. J. Woortman, G. O. R. Alberda van Ekenstein, K. Loos, *Polym. Chem.* **2015**, *6*, 5198–5211.

[12] A. Pellis, J. W. Comerford, A. J. Maneffa, M. H. Sipponen, J. H. Clark, T. J. Farmer, *Eur. Polym. J.* **2018**, *106*, 79–84.
